# Zinc (II)-Mediated Selective *O*-Benzylation of 2-Oxo-1,2-Dihydropyridines Systems

**DOI:** 10.3390/molecules23071784

**Published:** 2018-07-20

**Authors:** Qifan Zhou, Fangyu Du, Xinjie Liang, Wenqiang Liu, Ting Fang, Guoliang Chen

**Affiliations:** Key Laboratory of Structure-Based Drug Design & Discovery of Ministry of Education, Shenyang Pharmaceutical University, Shenyang 110016, China; zhouqifan921@126.com (Q.Z.); dufangyu1993@163.com (F.D.); renashdol@outlook.com (X.L.); liuwenqiang125@163.com (W.L.); yzftdyx@126.com (T.F.)

**Keywords:** *O*-benzylation, *N*-benzylation, 2-oxo-1,2-dihydropyridines, zinc (II), selectivity

## Abstract

The selective *O*-benzylation of 2-oxo-1,2-dihydropyridines plays a critical role in organic synthesis of natural products and biological active molecules. Herein we report a novel ternary system of ZnO, ZnCl_2_ and *N*,*N*-diisopropylethylamine (DIEA), that is highly effective for selective *O*-benzylation of 2-oxo-1,2-dihydropyridines using abundant substituted benzyl halides and related substituted 2-oxo-1,2-dihydropyridines compounds. This process allows access to a variety of *O*-benzyl products under mild reaction conditions, which are important synthetic intermediates in the protection of functional groups, and represents a new method toward the development for the *O*-benzylation of 2-oxo-1,2-dihydropyridines.

## 1. Introduction

**Scheme 1:**
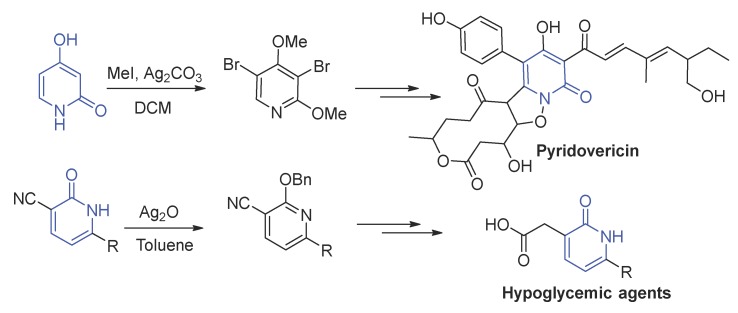
The synthetic routes of pyridomacrolidin and hypoglycemic agents.

2-Oxo-1,2-dihydropyridines fragments are ubiquitous reagents in organic synthesis, and some natural compounds with this structure have emerged as potent antitumor antiviral and attention [1]. Meanwhile, they are widely used as peptide mimics, which can generate similar physiological activity with peptides, such as protease [2,3], thrombin inhibitor [4,5], elastase [6,7], and caspase [8,9]. There is no doubt that the selective *O*-alkylation of 2-oxo-1,2-dihydropyridines fragments plays a critical role in organic synthesis of natural product and active molecule. For example, total synthesis of pyridomacrolidin [10], the pyridone building block was successfully constructed by selective bis-*O*-methylation protection and then deprotection (Scheme 1). Youngdale et al. [11] synthesized a class of oral hypoglycemic agents via sequences reaction, in which selective *O*-benzylation reaction was the critical step (Scheme 1).

The 2-oxo-1,2-dihydropyridines system is identified as an ambident anion under alkaline atmosphere and can form two reaction sites through tautomerism. It is well-known that this reaction can give both nitrogen and oxygen alkylation. The alkylation reaction is very sensitive to the reaction conditions, such as the type of counterions and leaving groups, solvents and alkylating agents, and temperature [12,13,14,15]. As selective *O*-alkylation and *N*-alkylation play vital roles in organic synthesis, more and more methods for selective alkylation have been reported.

**Scheme 2:**
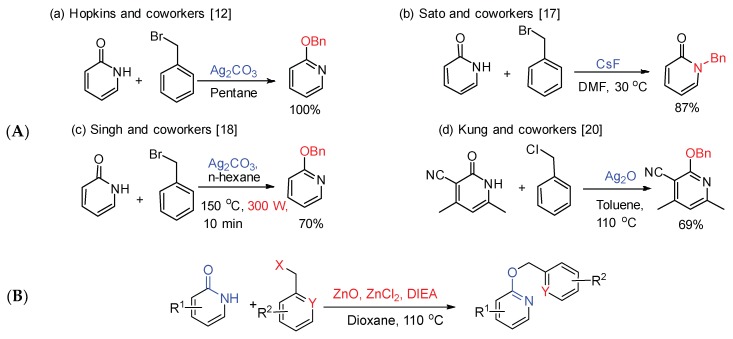
Metal-mediated benzylation of ambident anions. (**A**) Previous work about benzylation of ambident anions; (**B**) In this work.

According to reports [16], silver salts promoted *O*-alkylation of 2-pyridones, and alkaline metal salts contributed to *N*-alkylation. Sato et al. [17] utilized cesium fluoride as an efficient catalyst to obtain *N*-alkylation in suitable yield, and the selectivity of alkylation mainly depended on the kind of alkyl halide (Scheme 2). Singh et al. [18] reported a microwave-assisted protocol for the selective *O*-alkylation of aromatic imidate using silver carbonate as base (Scheme 2). Meanwhile, they also used other silver salts such as silver nitrate, silver oxide, silver acetate, and silver sulfate, etc., which could not afford desired compounds. Vavilina et al. [19] disclosed that ionic liquids accelerated the *O*-alkylation reaction of ambident 2-hydroxypyridine anion in comparison with molecular liquids using silver salt. Kung et al. [20] conducted a process of selective *O*-benzylation using silver oxide in the synthesis of 2-pyridone derivatives, which used in the treatment of leukemia (Scheme 2). Apart from Ag_2_CO_3_, and Ag_2_O, there is another related work by Kumar et al. in the literature using NaI as heterogeneous promoter [21]. Although the selective *O*-benzylation product of 2-pyridone can be obtained in a moderate yield using silver oxide as base, the large consumption of expensive silver salts limits the large-scale synthesis of products.

Herein (Scheme 2), we describe our efforts to prepare *O*-benzylation of 2-oxo-1,2-dihydropyridines from the substituted 2-oxo-1,2-dihydropyridines and substituted benzyl halides using ZnO, ZnCl_2_ and *N*,*N*-diisopropylethylamine (DIEA). These reactions occur at 110 °C, under argon atmosphere and afford appropriate yields. This process is general with respect to both the substituted 2-oxo-1,2-dihydropyridines and substituted benzyl halides, which allows preparation of many complex of *O*-benzylation of 2-oxo-1,2-dihydropyridines.

## 2. Results and Discussion

### 2.1. Optimization of the Reaction Conditions

According to literature [20], 2-(benzyloxy)-4,6-dimethylnicotinonitrile was obtained using silver oxide as a base in 65% yield. It is considerate that silver oxide is too expensive, so we are committed to finding a cheaper alternative catalytic system as a replacement.

Initially, we tried to use zinc (II), a subgroup element, which was also expected to catalyze selective *O*-benzylation. Unfortunately, *O*-benzylation product was not detected by TLC when catalyzed by zinc oxide alone (Table 1, entry 1). Similar results were observed using binary system of zinc oxide and zinc chloride (Table 1, entry 2). Surprisingly, the desired *O*-benzylation product was acquired in 71% yield through adding additional triethylamine to this catalytic system (Table 1, entry 3). We reasoned that alkaline environment contributed to the *O*-benzylation reaction. From the perspective of yield, ternary system of ZnCl_2_/ZnO/Et_3_N has better catalytic performance than silver oxide. Afterwards, in order to identify which component was indispensable in the ternary system, the reaction was carried out utilizing zinc chloride and triethylamine, but the benzylation reaction did not occur (Table 1, entry 4). Interestingly, *N*-benzylation of product **5** was obtained in 15% yield, when using triethylamine as base without zinc oxide and zinc chloride (Table 1, entry 5). This clearly highlighted the importance of the application of ternary system of zinc oxide, zinc chloride and triethylamine which collaboratively promoted selective *O*-benzylation. Attempts to reduce the amount of catalyst (0.1 equivalent (equiv)) proved unsuccessful, leading to an incomplete reaction with a yield of only 25% (Table 1, entry 6). Meanwhile, the reaction was carried out in DIEA (1.1 equiv) and zinc salts (0.1 equiv), and experimental result displayed that the desired compound was obtained in 13% yield. The result proved that zinc salts also need to be stoichiometric (Table 1, entry 8). However, it was feasible to change the base with DIEA proving to be optimal in terms of yield and ease of use (Table 1, entries 7, 9). Efforts to optimize the reaction through modulation of the solvent proved successful, with 1,4-dioxane being the most effective in the screening reaction (Table 1, entries 9–12). Meanwhile, the benzylation product was not observed in polar solvents such as ethylene glycol, *N,N*-dimethylformamide or dimethyl sulfoxide, on the contrary, nonpolar solvents were favored. When potassium carbonate was used as a base, the main product was confirmed as *N*-benzylation of product **5** without any selectivity, along with unreacted starting material and desired compound (Table 1, entry 13). The same result was obtained using potassium carbonate and DMF (*N*-benzylation product **5** was characterized by ^1^H NMR, seeing supporting information page 46). Simultaneously, some conventional Lewis acids, such as FeCl_3_, AlCl_3_, CuCl_2_ were also examined in the standard conditions, and no satisfactory results were obtained (Table 1, entries 14–16). Based on optimized experimental data the optimal reaction conditions was determined (Table 1, entry 8) which used ZnO (1.1 equiv), ZnCl_2_ (1.1 equiv), DIEA (1.1 equiv) and dioxane. Under optimized conditions, the desired product **3a** was isolated in 90% yield on a 20 g scale (Table 1, entry 17). 

**Scheme 3:**
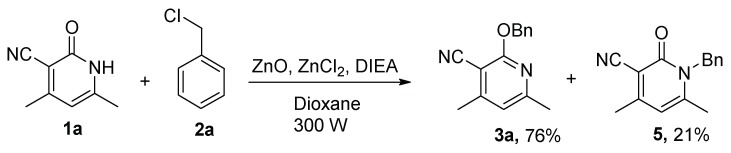
Microwave-assisted benzylation of **1a** and **2a**.

Microwave-assisted organic synthesis, a growing area in synthetic organic chemistry, is based on the empirical observation that some reactions proceed faster and result in higher yields under microwave irradiation than under conventional heating. To our knowledge there are only a few examples dealing with a comparison of the selective alkylation of the 2-pyridone system under conventional heating and microwave irradiation [22,23]. Therefore, we investigated the use of microwave irradiation to promote and activate *O*-benzylation of **1a** and **2a** (Scheme 3). Experimental data displayed that the reaction time was shortened from 24 h to 60 min under microwave irradiation. However, *N*-benzylation of product **5** and *O*-benzylation of product **3a** were obtained in 21% and 76% yield respectively, and without selectivity. Hence, microwaves were not used in the study of substrate suitability.

### 2.2. Scope with Respect to Substituted 2-Oxo-1,2-Dihydropyridines

With the optimal conditions in hand, we chose various of substituted 2-oxo-1,2-dihydropyridiness as substrates to investigate the scope and toleration of functional groups for this method (Table 2).

The reaction enjoys wide substrate scope with respect to substituted 2-oxo-1,2-dihydropyridines (Table 2). A range of functional groups on the 2-oxo-1,2-dihydropyridines proved to be compatible including methyl, ethyl, isopropyl, disubstituted alkyl. Interestingly, substrates with 4-methyl substitution afforded better yield than without 4-methy substitution. Although a more sterically encumbered 2-oxo-1,2-dihydropyridines substrate also reacted without incident, desired compounds were obtained in lower yield than other substrates. Not surprisingly, this reaction proceeded more slowly than those no-ring alkyl substitutions. We postulate that the lower yields of **3e** and **3f** may be caused the steric hindrance of the double ring was not conducive to the combination of benzyl groups. The efficiency for the monoalkyl substitution of 2-oxo-1,2-dihydropyridines was not as high as that for dialkyl substitution, and we predicted that because of the electron-donating nature of the alkyl, intermediates of transition with higher electron density would rapidly form under the reaction conditions. Strangely, **3j** was obtained in lower yield (62%). We conducted experiments again which displayed the same results, but *N*-benzylation of product was not observed by TLC. At the same time, longer reaction time did not increase yield of **3j**. The main reason was that the optimal condition was not suitable for this substrate. However, the suitability with respect to substrates will be further enlarged in subsequent experiments. Considering that electron-withdrawing effect of cyano on 2-oxo-1,2-dihydropyridine, the two substrates without cyano group of pyridin-2(*1H*)-one and 5-bromopyridin-2(*1H*)-one were tested under the standard reaction conditions. The results displayed that both substrates provided in high yield (**3l** and **3m**) and there was no clear relevance of cyano group to *O*-benzylation. This transformation allows direct construction of a substituted 2-oxo-1,2-dihydropyridines bearing *O*-benzylation substituent, which remains a significant role in organic synthesis.

### 2.3. Scope with Respect to Substituted Benzyl Halides

The ability of benzyl chloride to participate in the reaction opens the possibility for sequential benzylation. The scope of the reaction with respect to substituted benzyl halides is broad (Table 3).

A wide range of functional groups are tolerated, including methyl, chlorides, bromides, nitriles, nitro, methoxy and *tert*-butyl groups (Table 3). To our delight, most of the selected benzyl halides gave satisfactory results under the standard conditions. Both electron-rich and electron-poor benzyl halide participated differently well in the reaction. Benzyl halides with electron-withdrawing groups, such as nitro, nitrile, provided the *O*-benzylation product under the standard reaction conditions leading to a modest increase in the yield of target compound (92%). On the contrary, benzyl halides with electron-donating groups, such as methyl and *tert*-butyl, provided the *O*-benzylation product in slightly lower yield. At the same time, it was found that the position of the substituent has not much effect on the yield of *O*-benzylation. Heteroaromatic compounds of chloromethyl pyridines, bearing functional groups, also can be used in the reaction.

However, when iodomethane and iodoethane were used, the temperature had to be reduced to boiling point. It is regretful that desired compounds were not obtained. Meanwhile, alkylation reaction was also preceded in autoclave by standard reaction condition, leading to the same result. In all cases, *N*-benzylation product were not observed, which was identified by TLC (*N*-benzylation and *O*-benzylation products show large difference about R_f_). Certainly, the major byproducts of *N*-benzylation product were formed using potassium carbonate as base in *N*,*N*-dimethylformamide.

**Scheme 4:**
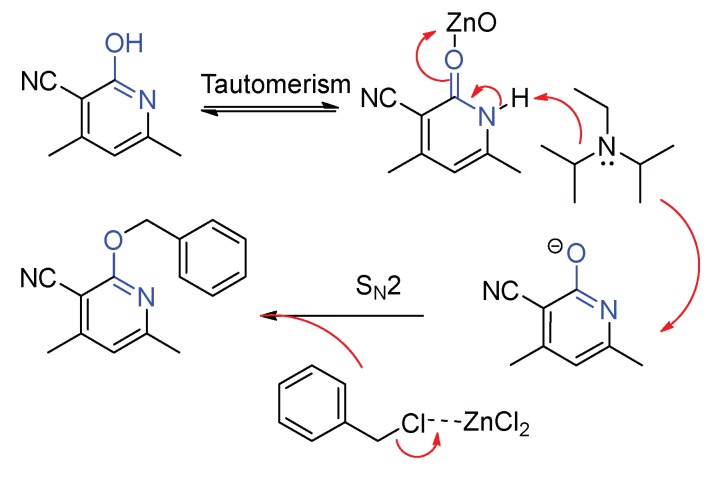
Possible mechanistic pathway.

According to the poor reaction performance in polar solvent, we postulate that these reactions proceed via a S_N_2 mechanism involving nucleophilic substitution from the electron-rich 2-oxo-1,2-dihydropyridines to the benzyl chloride (Scheme 4). Upon loss of hydrogen, this process generates an oxygen anion, which undergoes coupling with the transition state of benzylic cation to afford the desired compound.

## 3. Materials and Methods

### 3.1. Materials

All of the starting materials, reagents, and solvents are commercially available and used without further purification. The microwave-assisted reactions were performed using a CEM Discover System 908010 microwave apparatus (Charlotte, NC, USA). Melting points were determined with a X-4 apparatus and were uncorrected. The nuclear magnetic resonance (NMR) spectra were recorded on a Bruker 600 MHz spectrometer in CDCl_3_ or DMSO-*d*_6_ using tetramethylsilane (TMS) as an internal standard. Electrospray ionization mass spectrometry (ESI-MS) analyses were recorded in an Agilent 1100 Series MSD Trap SL (Santa Clara, CA, USA). The reactions were monitored by thin-layer chromatography (TLC: HG/T2354-92, GF254), and compounds were visualized on TLC with UV light.

### 3.2. Microwave-Assisted Synthesis of ***3a***

To a solution of **1a** (0.20 g, 1.35 mmol), zinc oxide (0.12 g, 1.48 mmol), zinc chloride (0.20 g, 1.48 mmol), *N*,*N*-diisopropylethylamine (0.19 g, 1.48 mmol), 1,4-dioxane (3 mL) was added benzyl chloride (0.2 g, 1.61 mmol). The mixture was irradiated at 110 °C for 60 min in a dedicated CEM Discover system, operating at a frequency of 2.45 GHz with continuous irradiation power from 0 to 300 W. After completion of the reaction, the insoluble residue was filtered off through celite, and the cake was washed with ethyl acetate (30 mL). The filtrate was washed with water (10 mL × 2), once with brine (10 mL), dried over magnesium sulfate, filtered, and concentrated in vacuo to afford crude product. The product was purified by column chromatography on silica gel (ethyl acetate: petroleum ether = 1:20) to afford *O*-benzylation in 65% yield and *N*-benzylation in 21% yield. 1-Benzyl-4,6-dimethyl-2-oxo-1,2-dihydropyridine-3-carbonitrile (**5**): Yellow solid, m.p. 104–107 °C, R_f_ = 0.30 (EtOAc:PE = 1:1). ^1^H-NMR (CDCl_3_, 600 MHz): δ 7.32 (t, *J* = 7.0 Hz, 2H), 7.28 (d, *J* = 7.3 Hz, 1H), 7.16 (d, *J* = 7.3 Hz, 2H), 6.01 (s, 1H), 5.32 (s, 2H), 2.41 (s, 3H), 2.33 (s, 3H).

### 3.3. General Procedures for the Synthesis of Substituted 2-Oxo-1,2-dihydropyridines of ***1a**–**1k***

A substituted alkyl methyl ketone or cyclic ketone (1 equiv) and ethyl formate or ethyl acetic (1 equiv) was added dropwise to absolute ether solution of sodium metal (1 equiv) for 1 h while maintained below 20 °C. After the addition, the reaction was allowed to stir in an ice bath until the sodium metal had disappeared. The precipitate was filtered, washed with absolute ether and dried to give the corresponding compound which was directly used for the next step without further purification.

To a solution of previous product (1 equiv), and cyanoacetamide (1.05 equiv) in water was stirred 6 min at room temperature. The mixture was added dropwise piperidine acetate solution (0.3 equiv), which was prepared from piperidine (1 equiv), acetic acid (1 equiv) and water (5 equiv). The solution was heated to reflux for 2 h. Then, the reactor was cooled to room temperature, and adjusted to pH 4 by 4 N hydrochloric acid. The resulting solid was filtered, respectively washed with water and ether, and dried to give the corresponding compound which was purified by recrystallizing using menthol as solvent.

*4,6-Dimethyl-2-oxo-1,2-dihydropyridine-3-carbonitrile* (**1a**): 58% yield**,** white solid, m.p. 264–266 °C (lit. [24] 294 °C), ^1^H-NMR (DMSO-*d*_6_, 600 MHz): δ 12.31 (br, 1H), 6.16 (s, 1H), 2.30 (s, 3H), 2.22 (s, 3H).

*6-Methyl-2-oxo-1,2-dihydropyridine-3-carbonitrile* (**1b**): 58% yield, yellow solid, m.p. 295–297 °C (lit. [25] 293–294 °C), ^1^H-NMR (DMSO-*d*_6_, 600 MHz): δ 12.56 (br, 1H), 8.01 (d, *J* = 6.1 Hz, 1H), 6.21 (d, *J* = 5.7 Hz, 1H), 2.27 (s, 3H).

*5,6-Dimethyl-2-oxo-1,2-dihydropyridine-3-carbonitrile* (**1c**): 54% yield, yellow solid, m.p. 223–225 °C (lit. [26] 223–225 °C), ^1^H-NMR (DMSO-*d*_6_, 600 MHz): δ 12.46 (br, 1H), 7.94 (s, 1H), 2.23 (s, 3H), 1.98 (s, 3H).

*6-Isobutyl-2-oxo-1,2-dihydropyridine-3-carbonitrile* (**1d**): 33% yield, white solid, m.p. 140–142 °C (lit. [27] 149–150 °C), ^1^H-NMR (DMSO-*d*_6_, 600 MHz): δ 12.52 (br, 1H), 8.04 (d, *J* = 7.3 Hz, 1H), 6.21 (d, *J* = 7.4 Hz, 1H), 2.41 (d, *J* = 7.4 Hz, 2H), 1.98–1.91 (m, 1H), 0.87 (d, *J* = 6.6 Hz, 6H).

*2-Oxo-2,5,6,7-tetrahydro-1H-cyclopenta[b]pyridine-3-carbonitrile* (**1e**): 21% yield, yellow solid, m.p. > 300 °C, ^1^H-NMR (DMSO-*d*_6_, 600 MHz): δ 12.75 (br, 1H), 8.01 (s, 1H), 2.80 (t, *J* = 7.6 Hz, 2H), 2.64 (t, *J* = 7.3 Hz, 2H), 2.06–2.01 (m, 2H).

*2-Oxo-1,2,5,6,7,8-hexahydroquinoline-3-carbonitrile* (**1f**): 25% yield, yellow solid, m.p. 248–249 °C (lit. [28] 245–248 °C), ^1^H-NMR (DMSO-*d*_6_, 600 MHz): δ 11.64 (br, 1H), 7.89 (s, 1H), 2.56 (t, *J* = 6.1 Hz, 2H), 2.42 (t, *J* = 6.2 Hz, 2H), 1.70–1.60 (m, 4H).

*6-Ethyl-4-methyl-2-oxo-1,2-dihydropyridine-3-carbonitrile* (**1g**): 24% yield, white solid, m.p. 230–233 °C (lit. [29] 243–244 °C), ^1^H-NMR (DMSO-*d*_6_, 600 MHz): δ 12.27 (br, 1H), 6.19 (s, 1H), 2.52–2.48 (q, 4H), 2.32 (s, 3H), 1.15 (t, *J* = 7.6 Hz, 3H).

*6-Isobutyl-4-methyl-2-oxo-1,2-dihydropyridine-3-carbonitrile* (**1h**): 42% yield, white solid, m.p. 169–172 °C, ^1^H-NMR (DMSO-*d*_6_, 600 MHz): δ 12.27 (br, 1H), 6.17 (s, 1H), 2.36 (d, *J* = 7.4 Hz, 2H), 2.32 (s, 3H), 1.97–1.90 (m, 1H), 0.87 (d, *J* = 6.6 Hz, 6H).

*4-Methyl-2-oxo-1,2,5,6,7,8-hexahydroquinoline-3-carbonitrile* (**1i**): 31% yield, white solid, m.p. 247–250 °C (lit. [30] 308–310 °C), ^1^H-NMR (DMSO-*d*_6_, 600 MHz): δ 12.32 (br, 1H), 2.72 (t, *J* = 5.9 Hz, 2H), 2.38 (t, *J* = 6.3 Hz, 2H), 2.21 (s, 3H), 1.68–1.66 (m, 4H).

*2-Oxo-6-propyl-1,2-dihydropyridine-3-carbonitrile* (**1j**): 41% yield, yellow solid, m.p. 141–143 °C (lit. [28] 151–152 °C), ^1^H-NMR (DMSO-*d*_6_, 600 MHz): δ 12.54 (br, 1H), 8.04 (d, *J* = 7.4 Hz, 1H), 6.23 (d, *J* = 7.3 Hz, 1H), 2.52–2.49 (t, 2H), 1.63–1.57 (m, 2H), 0.88 (t, *J* = 7.4 Hz, 3H).

*4-Methyl-2-oxo-6-propyl-1,2-dihydropyridine-3-carbonitrile* (**1k**): 25% yield, white solid, m.p. 154–157 °C (lit. [31] 210–211 °C), ^1^H-NMR (DMSO-*d*_6_, 600 MHz): δ 12.29 (br, 1H), 6.19 (s, 1H), 2.46 (t, *J* = 7.4 Hz, 2H), 2.32 (s, 3H), 1.64–1.56 (m, 2H), 0.88 (t, 7.4 Hz, 3H).

### 3.4. General Procedures for the Synthesis of ***3a**–**3m***

To a solution of substituted 2-oxo-1,2-dihydropyridines (3.36 mmol), zinc oxide (0.30 g, 3.70 mmol), zinc chloride (0.50 g, 3.70 mmol), *N*,*N*-diisopropylethylamine (0.48 g, 3.70 mmol), 1,4-dioxane (15 mL) was added benzyl chloride (0.58 g, 4.04 mmol) under argon atmosphere. The mixture was heated in 110 °C oil bath with rapid stirring for the indicated time. The reactor was cooled to room temperature, and the insoluble residue was filtered off through celite, and the cake was wash with ethyl acetate (30 mL). The filtrate was washed with water (10 mL × 2), once with brine (10 mL), dried over magnesium sulfate, filtered, and concentrated in vacuo to afford crude product. The product was purified by column chromatography on silica gel (ethyl acetate: petroleum ether = 1:20) to yield the corresponding compounds.

*2-(Benzyloxy)-4,6-dimethylnicotinonitrile* (**3a**): Yield: 0.72 g (90%), white solid, m.p. 86–87 °C, R_f_ = 0.70 (EtOAc:PE = 1:10). ^1^H-NMR (CDCl_3_, 600 MHz): δ 7.49 (d, *J* = 7.5 Hz, 2H), 7.37 (t, *J* = 7.3 Hz, 2H), 7.31 (t, *J* = 7.4 Hz, 1H), 6.69 (s, 1H), 5.48 (s, 2H), 2.45 (s, 3H), 2.44 (s, 3H). ^13^C-NMR (CDCl_3_, 150 MHz): δ 163.66, 160.58, 154.48, 136.55, 128.45, 129.91, 127.85, 117.71, 114.97, 94.15, 68.14, 24.55, 20.08. HRMS (ESI): *m*/*z* [M + Na]^+^ calculated for C_15_H_14_N_2_ONa: 261.1004, found: 261.1010.

*2-(Benzyloxy)-6-methylnicotinonitrile* (**3b**): Yield: 0.55 g (73%), white solid, m.p. 80–81 °C (lit. [32] 106 °C), R_f_ = 0.75 (EtOAc:PE = 1:10). ^1^H-NMR (CDCl_3_, 600 MHz): δ 7.74 (d, *J* = 7.6 Hz, 1H), 7.37 (t, *J* = 7.3 Hz, 2H), 7.32 (t, *J* = 7.4 Hz, 1H), 6.82 (d, *J* = 7.7 Hz, 1H), 5.50 (s, 2H). ^13^C-NMR (CDCl_3_, 150 MHz): δ 163.04, 162.00, 142.91, 136.31, 128.45, 127.99, 127.93, 116.06, 115.63, 93.54, 68.21, 24.76. HRMS (ESI): *m*/*z* [M + Na]^+^ calculated for C_14_H_12_N_2_ONa: 247.0847, found: 247.0851.

*2-(Benzyloxy)-5,6-dimethylnicotinonitrile* (**3c**): Yield: 0.70 g (88%), white solid, m.p. 79–81 °C, R_f_ = 0.72 (EtOAc:PE = 1:10). ^1^H-NMR (CDCl_3_, 600 MHz): δ 7.60 (s, 1H), 7.49 (d, *J* = 7.3 Hz, 2H), 7.37 (t, *J* = 7.3 Hz, 2H), 7.30 (t, *J* = 7.3 Hz, 1H), 5.48 (s, 2H), 2.45 (s, 2H), 2.21 (s, 3H). ^1^H-NMR (CDCl_3_, 150 MHz): δ 163.06, 162.04, 142.93, 136.35, 128.48, 128.02, 127.95, 116.11, 115.66, 93.55, 68.24, 24.79. HRMS (ESI): *m*/*z* [M + Na]^+^ calculated for C_15_H_14_N_2_ONa: 261.1004, found: 261.1008.

*2-(Benzyloxy)-6-isobutylnicotinonitrile* (**3d**): Yield: 0.74 g (83%), light yellow liquid, R_f_ = 0.74 (EtOAc:PE = 1:10). ^1^H-NMR (CDCl_3_, 600 MHz): δ 7.74 (d, *J* = 7.7 Hz, 1H), 7.48 (d, *J* = 7.4 Hz, 2H), 7.36 (t, *J* = 7.3 Hz, 2H), 7.30 (t, *J* = 7.4 Hz, 1H), 6.77 (d, *J* = 7.7 Hz, 1H), 5.50 (s, 2H), 2.59 (d, *J* = 7.2 Hz, 2H), 2.16–2.09 (m, 1H), 0.91 (d, *J* = 6.7 Hz, 6H). ^13^C-NMR (CDCl_3_, 150 MHz): δ 165.12, 163.02, 142.71, 136.44, 128.42, 127.93, 127.88, 116.31, 115.68, 93.64, 68.17, 47.40, 28.59, 22.39. HRMS (ESI): *m*/*z* [M + Na]^+^ calculated for C_17_H_18_N_2_ONa: 289.1317, found: 289.1323.

*2-(Benzyloxy)-6,7-dihydro-5H-cyclopenta[b]pyridine-3-carbonitrile* (**3e**): Yield: 0.51 g (61%), light yellow solid, m.p. 68–71 °C, R_f_ = 0.76 (EtOAc:PE = 1:10). ^1^H-NMR (CDCl_3_, 600 MHz): δ 7.65 (s, 1H), 7.49 (d, *J* = 7.4 Hz, 2H), 7.37 (t, *J* = 7.3 Hz, 2H), 7.31 (t, *J* = 7.4 Hz, 1H), 5.49 (s, 2H), 2.97 (t, *J* = 7.7 Hz, 2H), 2.88 (t, *J* = 7.5 Hz, 2H), 2.18–2.13 (m, 2H). ^13^C-NMR (CDCl_3_, 150 MHz): δ 169.05, 163.67, 138.17, 136.40, 129.59, 128.45, 127.92, 127.72, 116.20, 93.46, 68.29, 34.74, 29.55, 23.06. HRMS (ESI): *m*/*z* [M + Na]^+^ calculated for C_16_H_14_N_2_ONa: 273.1004, found: 273.1014.

*2-(Benzyloxy)-5,6,7,8-tetrahydroquinoline-3-carbonitrile* (**3f**): Yield: 0.58 g (66%), white solid, m.p. 67–68 °C, R_f_ = 0.74 (EtOAc:PE = 1:10). ^1^H-NMR (CDCl_3_, 600 MHz): δ 7.54 (s, 1H), 7.49 (d, *J* = 7.4 Hz, 2H), 7.37 (t, *J* = 7.3 Hz, 2H), 7.31 (t, *J* = 7.3 Hz, 1H), 5.46 (s, 2H), 2.83 (t, *J* = 6.3 Hz, 2H), 2.67 (t, *J* = 6.3 Hz, 2H), 1.88–1.84 (m, 2H), 1.81–1.77 (m, 2H). ^13^C-NMR (CDCl_3_, 150 MHz): δ 161.01, 160.43, 143.35, 136.60, 128.41, 127.91, 127.88, 125.20, 115.77, 93.70, 68.02, 32.74, 27.46, 22.44. HRMS (ESI): *m*/*z* [M + Na]^+^ calculated for C_17_H_16_N_2_ONa: 287.1160, found: 273.1173. 

*2-(Benzyloxy)-6-ethyl-4-methylnicotinonitrile* (**3g**): Yield: 0.73 g (86%), light yellow liquid, R_f_ = 0.78 (EtOAc:PE = 1:10). ^1^H-NMR (CDCl_3_, 600 MHz): δ 7.48 (dd, *J* = 1.3 Hz, 8.2 Hz, 2H), 7.36 (t, *J* = 7.3 Hz, 2H), 7.30 (t, *J* = 7.3 Hz, 1H), 6.68 (s, 1H), 5.50 (s, 2H), 2.71 (q, *J* = 7.6 Hz, 15.1 Hz, 2H), 2.45 (s, 3H), 1.26 (t, *J* = 7.8 Hz, 3H). ^13^C-NMR (CDCl_3_, 150 MHz): δ 165.49, 163.71, 154.55, 136.66, 129.39, 127.89, 127.86, 116.48, 115.01, 94.22, 68.02, 31.21, 20.13, 13.00. HRMS (ESI): *m*/*z* [M + Na]^+^ calculated for C_17_H_16_N_2_ONa: 287.1160, found: 273.1173.

*2-(Benzyloxy)-6-isobutyl-4-methylnicotinonitrile* (**3h**): Yield: 0.76 g (80%), Colorless liquid, R_f_ = 0.77 (EtOAc:PE = 1:10). ^1^H-NMR (CDCl_3_, 600 MHz): δ 7.48 (d, *J* = 7.4 Hz, 2H), 7.36 (t, *J* = 7.4 Hz, 2H), 7.29 (t, *J* = 7.3 Hz, 1H), 6.64 (s, 1H), 5.49 (s, 2H), 2.53 (d, *J* = 7.2 Hz, 2H), 2.45 (s, 3H), 2.15–2.08 (m, 1H), 0.90 (d, *J* = 6.6 Hz, 6H). ^13^C-NMR (CDCl_3_, 150 MHz): δ 163.69, 163.62, 154.22, 136.67, 128.39, 127.83, 127.81, 117.92, 115.01, 94.23, 68.08, 47.25, 28.50, 22.43, 20.12. HRMS (ESI): *m*/*z* [M + Na]^+^ calculated for C_18_H_20_N_2_ONa: 303.1473, found: 303.1479.

*2-(Benzyloxy)-4-methyl-5,6,7,8-tetrahydroquinoline-3-carbonitrile* (**3i**): Yield: 0.66 g (71%), white solid, m.p. 90–92 °C, R_f_ = 0.75 (EtOAc:PE = 1:10). ^1^H-NMR (CDCl_3_, 600 MHz): δ 7.49 (d, *J* = 7.4 Hz, 2H), 7.36 (t, *J* = 7.3 Hz, 2H), 7.29 (t, *J* = 7.4 Hz, 1H), 5.49 (s, 2H), 2.87 (t, *J* = 6.2 Hz, 2H), 2.55 (t, *J* = 5.9Hz, 2H), 2.41 (s, 3H), 1.84–1.76 (m, 4H). ^13^C-NMR (CDCl_3_, 150 MHz): δ 161.32, 159.29, 152.65, 136.90, 128.37, 127.80, 127.76, 124.23, 115.08, 93.42, 67.71, 28.51, 25.35, 22.53, 22.50, 21.49. HRMS (ESI): *m*/*z* [M + Na]^+^ calculated for C_18_H_18_N_2_ONa: 301.1317, found: 301.1360.

*2-(Benzyloxy)-6-propylnicotinonitrile* (**3j**): Yield: 0.46 g (62%), light yellow liquid, R_f_ = 0.73 (EtOAc:PE = 1:10). ^1^H-NMR (CDCl_3_, 600 MHz): δ 7.75 (d, *J* = 7.6 Hz, 1H), 7.49 (d, *J* = 7.2 Hz, 2H), 7.37 (t, *J* = 7.3 Hz, 2H), 7.31 (t, *J* = 7.3 Hz, 1H), 6.79 (d, *J* = 7.6 Hz, 1H), 5.51 (s, 2H), 2.71 (t, *J* = 7.4 Hz, 2H), 1.78–1.71 (m, 2H), 0.94 (t, *J* = 7.4 Hz, 3H). ^13^C-NMR (CDCl_3_, 150 MHz): δ 165.80, 163.13, 142.88, 136.46, 128.45, 127.97, 127.95, 115.92, 115.64, 93.67, 68.18, 40.25, 22.12, 13.76. HRMS (ESI): *m*/*z* [M + Na]^+^ calculated for C_16_H_16_N_2_ONa: 275.1160, found: 275.1159.

*2-(Benzyloxy)-4-methyl-6-propylnicotinonitrile* (**3k**): Yield: 0.78 g (82%), Colorless liquid, R_f_ = 0.72 (EtOAc:PE = 1:10). ^1^H-NMR (CDCl_3_, 600 MHz): δ 7.48 (d, *J* = 7.3 Hz, 2H), 7.36 (t, *J* = 7.1 Hz, 2H), 7.30 (t, *J* = 6.3 Hz, 1H), 6.67 (s, 1H), 5.49 (s, 2H), 2.65 (t, *J* = 7.4 Hz, 2H), 2.45 (s, 3H), 1.76–1.68 (m, 2H), 0.93 (t, *J* = 7.3 Hz, 3H). ^13^C-NMR (CDCl_3_, 150 MHz): δ 164.33, 163.70, 154.38, 136.66, 128.39, 127.84, 117.23, 115.01, 94.22, 68.06, 40.05, 22.09, 13.76. HRMS (ESI): *m*/*z* [M + Na]^+^ calculated for C_17_H_18_N_2_ONa: 289.1317, found: 289.1315.

*2-(Benzyloxy)pyridine* (**3l**)*:* Yield: 0.90 g (93%), Colorless liquid, R_f_ = 0.75 (EtOAc:PE = 1:10). ^1^H-NMR (CDCl_3_, 600 MHz): 8.18 (dd, *J* = 1.4, 5.0 Hz, 1H), 7.60–7.57 (m, 1H), 7.46 (d, *J* = 7.4 Hz, 2H), 7.37 (t, *J* = 5.7 Hz, 2H), 7.31 (t, *J* = 7.3 Hz, 1H), 6.89–6.87 (m, 1H), 6.81 (d, *J* = 8.3 Hz, 1H), 5.38 (s, 2H). ^13^C-NMR (CDCl_3_, 150 MHz): δ 163.6, 146.8, 138.6, 137.3, 128.4, 127.9, 127.8, 116.9, 111.3, 67.5. MS (ESI) *m*/*z* [M + H]^+^ 186.1.

*2-(Benzyloxy)-5-bromopyridine* (**3m**): Yield: (92%), White solid, R_f_ = 0.71 (EtOAc:PE = 1:10), m.p. 54–55 °C (lit.^33^ 56–58 °C), ^1^H-NMR (CDCl_3_, 600 MHz): 8.20 (d, *J* = 2.2 Hz, 1H), 7.61 (dd, *J* = 2.4, 8.8 Hz, 1H), 7.42 (d, *J* = 7.5 Hz, 2H), 7.35 (t, *J* = 7.3 Hz, 2H), 7.30 (t, *J* = 7.3 Hz, 1H), 6.69 (d, *J* = 8.8 Hz, 1H), 5.33 (s, 2H). ^13^C-NMR (CDCl_3_, 150 MHz): δ 162.4, 147.5, 141.2, 136.9, 128.6, 128.1, 128.0, 113.0, 112.0, 68.1. MS (ESI) *m*/*z* [M + H]^+^ 264.0, 265.9.

### 3.5. General Procedures for the Synthesis of ***4a**–**4k***

To a solution of **1a** (0.5 g, 3.36 mmol), zinc oxide (0.30 g, 3.70 mmol), zinc chloride (0.50 g, 3.70 mmol), *N*,*N*-diisopropylethylamine (0.48 g, 3.70 mmol), 1,4-dioxane (15 mL) was added substituted benzyl halides (4.04 mmol) under argon atmosphere. The mixture was heated in 110 °C oil bath with rapid stirring for the indicated time. The reactor was cooled to room temperature, and the insoluble residue was filtered off through celite, and the cake was wash with ethyl acetate (30 mL). The filtrate was washed with water (10 mL × 2), once with brine (10 mL), dried over magnesium sulfate, filtered, and concentrated in vacuo. The product was purified by column chromatography on silica gel (ethyl acetate: petroleum ether = 1:20) to yield the corresponding compounds.

*4,6-Dimethyl-2-((4-methylbenzyl)oxy)nicotinonitrile* (**4a**): Yield: 0.68 g (80%), white solid, m.p. 93–95 °C, R_f_ = 0.71 (EtOAc:PE = 1:10). ^1^H-NMR (CDCl_3_, 600 MHz): δ 7.38 (d, *J* = 7.9 Hz, 2H), 7.17 (d, *J* = 7.8 Hz, 2H), 6.68 (s, 1H), 5.44 (s, 2H), 2.45 (s, 3H), 2.43 (s, 3H), 2.35 (s, 3H). ^13^C-NMR (CDCl_3_, 150 MHz): δ 163.72, 160.52, 154.41, 137.68, 133.50, 129.09, 128.06, 117.59, 114.98, 94.13, 68.10, 24.53, 21.21, 20.04. HRMS (ESI): *m*/*z* [M + Na]^+^ calculated for C_16_H_16_N_2_ONa: 275.1160, found: 275.1169.

*2-((4-Chlorobenzyl)oxy)-4,6-dimethylnicotinonitrile* (**4b**): Yield: 0.78 g (85%), white solid, m.p. 119–121 °C, R_f_ = 0.72 (EtOAc:PE = 1:10). ^1^H-NMR (CDCl_3_, 600 MHz): δ 7.42 (d, *J* = 8.3 Hz, 2H), 7.33 (d, *J* = 8.3 Hz, 2H), 6.70 (s, 2H), 5.44 (s, 2H), 2.45 (s, 6H). ^13^C-NMR (CDCl_3_, 150 MHz): δ 163.39, 160.55, 154.56, 135.04, 133.71, 129.22, 128.61, 117.87, 114.85, 94.14, 67.33, 24.51, 20.06. HRMS (ESI): *m*/*z* [M + Na]^+^ calculated for C_15_H_13_ClN_2_ONa: 295.0614, found: 295.0616.

*2-(Benzyloxy)-4,6-dimethylnicotinonitrile* (**4c = 3a**): Yield: 0.72 g (91%), white solid, m.p. 86–87 °C, R_f_ = 0.70 (EtOAc:PE = 1:10). ^1^H-NMR (CDCl_3_, 600 MHz): δ 7.48 (d, *J* = 7.3 Hz, 2H), 7.36 (t, *J* = 7.3 Hz, 2H), 7.30 (t, *J* = 7.3 Hz, 1H), 6.68 (s, 1H), 5.48 (s, 2H), 2.45 (s, 3H), 2.43 (s, 3H). ^13^C-NMR (CDCl_3_, 150 MHz): δ 163.66, 160.59, 154.48, 136.55, 128.45, 127.91, 127.87, 127.85, 117.74, 114.97, 94.14, 68.14, 24.56, 20.07. HRMS (ESI): *m*/*z* [M + Na]^+^ calculated for C_15_H_14_N_2_ONa: 261.1004, found: 261.1010.

*2-((3,4-Dichlorobenzyl)oxy)-4,6-dimethylnicotinonitrile* (**4d**): Yield: 0.90 g (88%), white solid, m.p. 104–106 °C, R_f_ = 0.72 (EtOAc:PE = 1:10). ^1^H-NMR (CDCl_3_, 600 MHz): δ 7.57 (d, *J* = 1.6 Hz, 1H), 7.43 (d, *J* = 8.2 Hz, 1H), 7.34 (t, *J* = 1.7 Hz, 1H), 6.72 (s, 1H), 5.42 (s, 2H), 2.45 (s, 3H), 2.44 (s, 3H). ^13^C-NMR (CDCl_3_, 150 MHz): δ 163.10, 160.57, 154.68, 136.80, 132.47, 131.93, 130.47, 129.84, 127.18, 118.08, 114.72, 94.15, 66.62, 24.49, 20.07. HRMS (ESI): *m*/*z* [M + Na]^+^ calculated for C_15_H_12_Cl_2_N_2_ONa: 329.0224, found: 329.0222.

*2-((2,3-Dichlorobenzyl)oxy)-4,6-dimethylnicotinonitrile* (**4e**): Yield: 0.84 g (82%), white solid, m.p. 167–169 °C, R_f_ = 0.73 (EtOAc:PE = 1:10). ^1^H-NMR (CDCl_3_, 600 MHz): δ 7.55 (d, *J* = 7.7 Hz, 1H), 7.43 (d, *J* = 6.8 Hz, 1H), 7.24 (t, *J* = 7.9 Hz, 1H), 5.56 (s, 2H), 2.47 (s, 3H), 2.46 (s, 3H). ^13^C-NMR (CDCl_3_, 150 MHz): δ 163.14, 160.83, 154.62, 136.71, 132.98, 130.73, 129.56, 127.48, 126.65, 118.16, 114.79, 94.08, 65.59, 24.55, 20.10. HRMS (ESI): *m*/*z* [M + Na]^+^ calculated for C_15_H_12_Cl_2_N_2_ONa: 329.0224, found: 329.0232.

*2-((2,4-Dichlorobenzyl)oxy)-4,6-dimethylnicotinonitrile* (**4f**): Yield: 0.89 g (86%), white solid, m.p. 146–148 °C, R_f_ = 0.72 (EtOAc:PE = 1:10). ^1^H-NMR (CDCl_3_, 600 MHz): δ 7.56 (d, *J* = 8.3 Hz, 1H), 7.40 (d, *J* = 2.0 Hz, 1H), 7.28 (t, *J* = 2.0 Hz, 1H), 6.73 (s, 1H), 5.51 (s, 2H), 2.47 (s, 3H), 2.46 (s, 3H). ^13^C-NMR (CDCl_3_, 150 MHz): δ 163.11, 160.75, 154.60, 134.08, 133.47, 132.99, 130.91, 129.80, 127.24, 118.11, 114.74, 94.07, 64.88, 24.51, 20.07. HRMS (ESI): *m*/*z* [M + Na]^+^ calculated for C_15_H_12_Cl_2_N_2_O Na: 329.0224, found: 329.0219.

*4,6-Dimethyl-2-((4-nitrobenzyl)oxy)nicotinonitrile* (**4g**): Yield: 0.87 g (92%), white yellow solid, m.p. 152–154 °C, R_f_ = 0.50 (EtOAc:PE = 1:10). ^1^H-NMR (CDCl_3_, 600 MHz): δ 8.24 (d, *J* = 8.7 Hz, 2H), 7.66 (d, *J* = 8.7 Hz, 2H), 6.75 (s, 1H), 5.58 (s, 2H), 2.48 (s, 3H), 2.45 (s, 3H). ^13^C-NMR (CDCl_3_, 150 MHz): δ 162.98, 160.68, 154.81, 147.5, 144.01, 127.94, 123.74, 118.31, 114.67, 94.20, 66.74, 24.49, 20.09. HRMS (ESI): *m*/*z* [M + Na]^+^ calculated for C_15_H_13_N_3_O_3_Na : 306.0855, found: 306.0852.

*2-((4-Bromobenzyl)oxy)-4,6-dimethylnicotinonitrile* (**4h**): Yield: 0.97 g (92%), white solid, m.p. 122–124 °C, R_f_ = 0.68 (EtOAc:PE = 1:10). ^1^H-NMR (CDCl_3_, 600 MHz): δ 7.49 (d, *J* = 8.3 Hz, 2H), 7.36 (d, *J* = 8.3 Hz, 2H), 6.70 (s, 1H), 5.42 (s, 2H), 2.45 (s, 3H), 2.44 (s, 3H). ^13^C-NMR (CDCl_3_, 150 MHz): δ 163.37, 160.55, 154.57, 135.56, 131.57, 129.53, 121.86, 117.89, 114.83, 94.16, 67.35, 24.51, 20.07. HRMS (ESI): *m*/*z* [M + Na]^+^ calculated for C_15_H_12_Cl_2_N_2_ONa: 329.0224, found: 329.0219.

*2-((4-(tert-Butyl)benzyl)oxy)-4,6-dimethylnicotinonitrile* (**4i**): Yield: 0.81 g (82%), white solid, m.p. 80–82 °C, R_f_ = 0.76 (EtOAc:PE = 1:10). ^1^H-NMR (CDCl_3_, 600 MHz): δ 7.42 (d, *J* = 8.2 Hz, 2H), 7.39 (d, *J* = 8.2 Hz, 2H), 6.68 (s, 2H), 5.44 (s, 2H), 2.45 (s, 3H), 2.43 (s, 3H), 1.32 (s, 9H). ^13^C-NMR (CDCl_3_, 150 MHz): δ 163.79, 160.58, 154.44, 150.93, 133.51, 127.85, 125.38, 117.63, 115.02, 94.14, 68.09, 34.60, 31.37, 24.58, 20.07. HRMS (ESI): *m*/*z* [M + Na]^+^ calculated for C_19_H_22_N_2_ONa: 317.1630, found: 317.1642.

*2-((4-Cyanobenzyl)oxy)-4,6-dimethylnicotinonitrile* (**4j**): Yield: 0.81 g (92%), white solid, m.p. 173–175 °C, R_f_ = 0.53 (EtOAc:PE = 1:10). ^1^H-NMR (CDCl_3_, 600 MHz): δ 7.66 (d, *J* = 8.1 Hz, 2H), 7.59 (d, *J* = 8.2 Hz, 2H), 6.74 (s, 1H), 5.53 (s, 2H), 2.47 (s, 3H), 2.44 (s, 3H). ^13^C-NMR (CDCl_3_, 150 MHz): δ 163.05, 160.67, 154.76, 142.01, 132.32, 127.87, 118.75, 118.25, 114.70, 111.63, 94.17, 66.97, 24.50, 20.09. HRMS (ESI): *m*/*z* [M + Na]^+^ calculated for C_16_H_13_N_3_ONa: 286.0956, found: 286.0949.

*2-((3,4-Dimethoxypyridin-2-yl)methoxy)-4,6-dimethylnicotinonitrile* (**4k**): Yield: 0.81 g (80%), white solid, m.p. 137–139 °C, R_f_ = 0.50 (EtOAc:PE = 1:5). ^1^H-NMR (CDCl_3_, 600 MHz): δ 8.26 (d, *J* = 5.5 Hz, 1H), 6.87 (d, *J* = 5.5 Hz, 1H), 6.70 (s, 1H), 5.56 (s, 2H), 3.95 (s, 3H), 3.94 (s, 3H), 2.46 (s, 3H), 2.44 (s, 3H). ^13^C-NMR (CDCl_3_, 150 MHz): δ 163.74, 160.69, 159.01, 154.38, 149.69, 145.81, 145.17, 117.68, 115.08, 107.96, 93.88, 66.04, 61.75, 55.76, 24.56, 20.07. HRMS (ESI): *m*/*z* [M + Na]^+^ calculated for C_16_H_17_N_3_O_3_Na: 322.1168, found: 322.1183.

Supplementary Materials contain all obtained ^1^H-NMR and ^13^C-NMR spectra of compounds presented in the manuscript.

## 4. Conclusions

In summary, we have developed a ternary system for the selective *O*-benzylation of 2-oxo-1,2-dihydropyridines systems that utilizes readily available ZnO, ZnCl_2_, DIEA, substituted benzyl halides and related 2-oxo-1,2-dihydropyridines compounds. This protocol proposes a novel method in C-O bond construction and provides some examples of selective *O*-benzylation of 2-oxo-1,2-dihydropyridines using readily available starting materials under mild reaction conditions. This reaction allows the conversion of simple starting materials to complex *O*-benzyl products, which are important synthetic intermediates in the protection of functional groups. Efforts to apply our Zn-based system to other 2-oxo-1,2-dihydropyridines and to expand the scope of the benzylation reactions to other classes of alkylation are currently underway in our laboratory.

## Figures and Tables

**Table 1:**
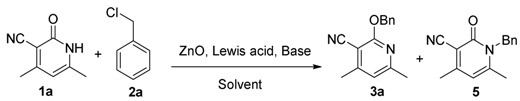
Optimization of the reaction conditions for the *O*-benzylation of **1a** and **2a** ^1^.

Entry	ZnO (Equiv)	Lewis Acid (Equiv)	Base (Equiv)	Solvent	Time (h)	Yield (%) ^2^	
						3a	5
Entry 1	1.1	--	--	Toluene	24	-- ^3^	-- ^3^
Entry 2	1.1	ZnCl_2_ (1.1)	--	Toluene	24	-- ^3^	-- ^3^
Entry 3	1.1	ZnCl_2_ (1.1)	TEA (1.1)	Toluene	24	71	-- ^3^
Entry 4	--	ZnCl_2_ (1.1)	TEA (1.1)	Toluene	24	-- ^3^	-- ^3^
Entry 5	--	--	TEA (1.1)	Toluene	24	-- ^4^	15
Entry 6	0.1	ZnCl_2_ (0.1)	TEA (0.1)	Toluene	36	25	-- ^3^
Entry 7	1.1	ZnCl_2_ (1.1)	TEA (1.1)	Dioxane	24	64	-- ^3^
Entry 8	0.1	ZnCl_2_ (0.1)	DIEA (1.1)	Dioxane	24	13	-- ^3^
Entry 9	1.1	ZnCl_2_(1.1)	DIEA (1.1)	Dioxane	24	90	-- ^3^
Entry 10	1.1	ZnCl_2_ (1.1)	DIEA (1.1)	Ethylene glycol	24	-- ^3^	-- ^3^
Entry 11	1.1	ZnCl_2_ (1.1)	DIEA (1.1)	DMF	24	-- ^3^	-- ^3^
Entry 12	1.1	ZnCl_2_ (1.1)	DIEA (1.1)	DMSO	24	-- ^3^	-- ^3^
Entry 13	1.1	ZnCl_2_ (1.1)	K_2_CO_3_ (1.1)	DMF	24	21	73
Entry 14	1.1	AlCl_3_ (1.1)	DIEA (1.1)	Dioxane	24	-- ^3^	-- ^3^
Entry 15	1.1	FeCl_3_ (1.1)	DIEA (1.1)	Dioxane	24	-- ^3^	-- ^3^
Entry 16	1.1	CuCl_2_ (1.1)	DIEA (1.1))	Dioxane	24	-- ^3^	-- ^3^
Entry 17 ^5^	1.1	ZnCl_2_ (1.1)	DIEA (1.1)	Dioxane	24	86	-- ^3^

^1^ Unless otherwise noted, the reactions were performed with **1a** (3.36 mmol), **2a** (4.04 mmol), zinc oxide (3.70 mmol), zinc chloride (3.70 mmol), *N*,*N*-diisopropylethylamine (3.70 mmol) in 1,4-dioxane (15 mL) under argon atmosphere at 110 °C. ^2^ Isolated yield after column chromatography. ^3^ No reaction was detected by thin-layer chromatography (TLC). ^4^ *N*-benzylation of product **5** was obtained in 15% yield. ^5^ The load of **1a** was 20 g.

**Table 2:**
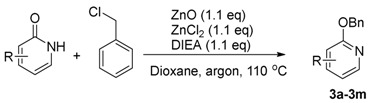
Scope with respect to *O*-benzylation of substituted 2-oxo-1,2-dihydropyridines with benzyl chloride ^1^.

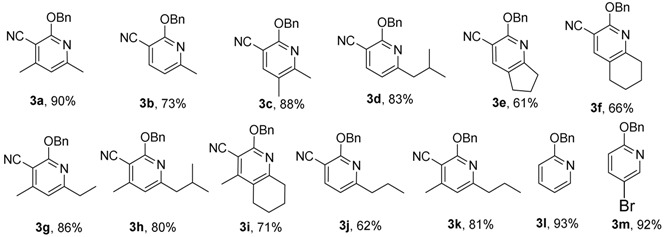

^1^ Unless noted otherwise, the reactions were performed with 2-oxo-1,2-dihydropyridines (3.36 mmol), benzyl chloride (4.04 mmol), zinc oxide (3.70 mmol), zinc chloride (3.70 mmol), *N*,*N*-diisopropylethylamine (3.70 mmol) in 1,4-dioxane (15 mL) and isolated after column chromatography.

**Table 3:**
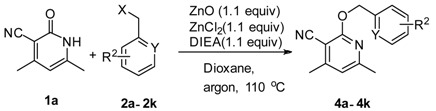
Scope with respect to *O*-benzylation of **1a** with substituted benzyl halides ^1^.

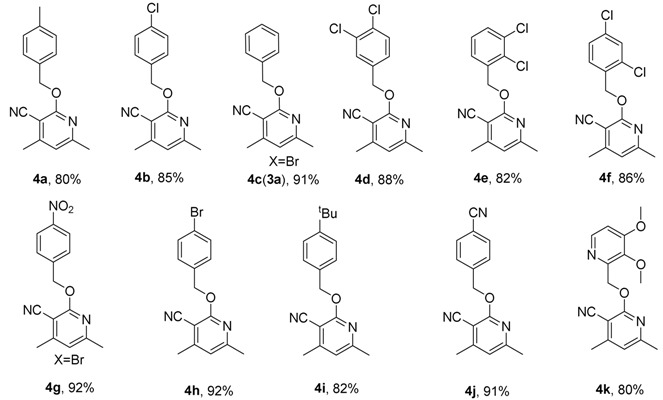

^1^ Unless noted otherwise, the reactions were performed with **1a** (3.36 mmol), zinc oxide (3.70 mmol), zinc chloride (3.70 mmol), *N*,*N*-diisopropylethylamine (3.70 mmol), substituted benzyl halides (4.04 mmol) in 1,4-dioxane (15 mL) and isolated after column chromatography.

## Supplementary Materials

The following are available online.
