# Supplementary material for: Zinc (II)-Mediated Selective O-Benzylation of 2-Oxo-1,2-Dihydropyridines Systems
Source: Molecules. 2018 Jul 20;23(7):1784. doi: 10.3390/molecules23071784 (PMC6100589; doi:10.3390/molecules23071784)

Supporting Information

Zinc (II)-Mediated Selective *O*-Benzylation of 2-Oxo-1,2-dihydropyridines Systems

Qifan Zhou, Fangyu Du, Xinjie Liang, Wenqiang Liu, Ting Fang, Guoliang Chen*

Key Laboratory of Structure-Based Drug Design & Discovery, Ministry of Education, Shenyang Pharmaceutical University, Shenyang 110016, China

*Correspond author: Guoliang Chen (E-mail: chenguoliang@syphu.edu.cn)

Table of Contents

1. General procedures ... 1
2. Microwave-assisted synthesis of 3a ... 1
3. General procedures for the synthesis of substituted aromatic lactam of 1a-1k ... 1
4. General procedures for the synthesis of 3a-3m ... 2
5. General procedures for the synthesis of 4a-4k ... 2
6. ^{1}H NMR spectra of 1a-1k ... 3
7. ^{1}H and ^{13}C NMR spectra and HRMS spectra of 3a-3m ... 9
8. ^{1}H and ^{13}C NMR spectra and HRMS spectra of 4a-4k ... 30
9. ^{1}H NMR spectra of *N*-benzylation product 5 ... 46

1. General procedures

All of the starting materials, reagents, and solvents are commercially available and used without further purification. The microwave-assisted reactions were performed using a CEM Discover System 908010 microwave apparatus (Matthews, NC, USA). Melting points were determined with a X-4 apparatus and were uncorrected. The nuclear magnetic resonance (NMR) spectra were recorded on a Bruker 600 MHz spectrometer in $CDCl_3$ or DMSO-d_6 using tetramethylsilane (TMS) as an internal standard. Electrospray ionization mass spectrometry (ESI-MS) analyses were recorded in an Agilent 1100 Series MSD Trap SL (Santa Clara, CA, USA). The reactions were monitored by thin-layer chromatography (TLC: HG/T2354-92, GF254), and compounds were visualized on TLC with UV light.

2. Microwave-assisted synthesis of 3a

To a solution of **1a** (0.20 g, 1.35 mmol), zinc oxide (0.12 g, 1.48 mmol), zinc chloride (0.20 g, 1.48 mmol), *N*,*N*-diisopropylethylamine (0.19 g, 1.48 mmol), 1,4-dioxane (3 mL) was added benzyl chloride (0.2 g, 1.61 mmol). The mixture was irradiated at 110 °C for 60 min in a dedicated CEM-Discover system, operating at a frequency of 2.45 GHz with continuous irradiation power from 0 to 300 W. After completion of the reaction, the insoluble residue was filtered off through celite, and the cake was wash with ethyl acetate (30 mL). The filtrate was washed with water (10 mL×2), once with brine (10 mL), dried over magnesium sulfate, filtered, and concentrated *in vacuo* to afford crude product. The product was purified by column chromatography on silica gel (ethyl acetate: petroleum ether=1:20) to afford *O*-benzylation in 65% yield and *N*-benzylation in 21% yield.

3. General procedures for the synthesis of substituted aromatic lactam of 1a-1k

A substituted alkyl methy ketone or cyclic ketone (1 equiv) and ethyl formate or

ethyl acetic (1 equiv) was added dropwise to absolute ether solution of sodium metal (1 equiv) for 1 hour while maintained below 20 ºC. After the addition, the reaction was allowed to stir at ice bath until the sodium metal was disappeared. The precipitate was filtered, washed with absolute ether and dried to give the corresponding compound which was directly used next step without purification.

To a solution of previous product (1 equiv), and cyanoacetamide (1.05 equiv) in water was stirred 6 minutes at room temperature. The mixture was added dropwise piperidine acetate solution (0.3 equiv), which was prepared from piperidine (1 equiv), acetic acid (1 equiv) and water (5 equiv). The solution was heated to reflux for 2 hours. Then, the reactor was cooled to room temperature, and adjusted to pH 4 by 4 N hydrochloric acid. The resulting solid was filtered, respectively washed with water and ether, and dried to give the corresponding compound which was purified by recrystallizing using menthol as solvent.

4. General procedures for the synthesis of 3a-3m

To a solution of substituted aromatic lactam (3.36 mmol), zinc oxide (0.30 g, 3.70 mmol), zinc chloride (0.50 g, 3.70 mmol), *N*,*N*-diisopropylethylamine (0.48 g, 3.70 mmol), 1,4-dioxane (15 mL) was added benzyl chloride (0.58 g, 4.04 mmol) under argon atmosphere. The mixture was heated in 110ºC oil bath with rapid stirring for the indicated time. The reactor was cooled to room temperature, and the insoluble residue was filtered off through celite, and the cake was wash with ethyl acetate (30 mL). The filtrate was washed with water (10 mL×2), once with brine (10 mL), dried over magnesium sulfate, filtered, and concentrated *in vacuo* to afford crude product. The product was purified by column chromatography on silica gel (ethyl acetate: petroleum ether=1:20) to yield the corresponding compounds.

5. General procedures for the synthesis of 4a-4k

To a solution of **1a** (0.5 g, 3.36 mmol), zinc oxide (0.30 g, 3.70 mmol), zinc chloride (0.50 g, 3.70 mmol), *N*,*N*-diisopropylethylamine (0.48 g, 3.70 mmol),

1,4-dioxane (15 mL) was added substituted benzyl halides (4.04 mmol) under argon atmosphere. The mixture was heated in 110 ºC oil bath with rapid stirring for the indicated time. The reactor was cooled to room temperature, and the insoluble residue was filtered off through celite, and the cake was wash with ethyl acetate (30 mL). The filtrate was washed with water (10 mL×2), once with brine (10 mL), dried over magnesium sulfate, filtered, and concentrated *in vacuo*. The product was purified by column chromatography on silica gel (ethyl acetate: petroleum ether=1:20) to yield the corresponding compounds.

6. ^{1}H NMR spectra of 1a-1k

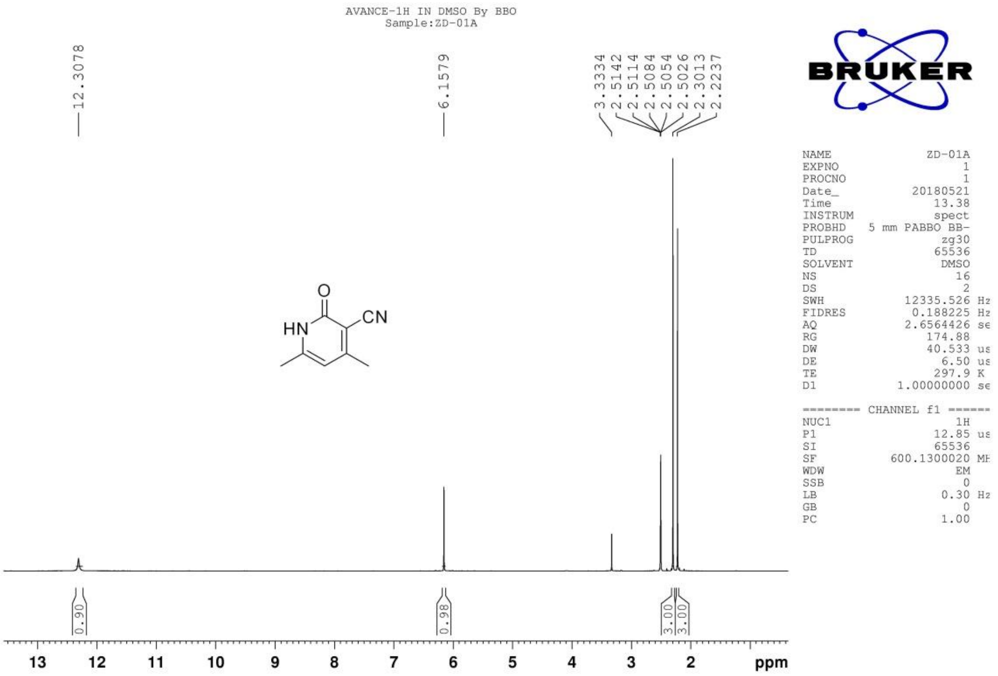

^{1}H NMR spectra of **1a**

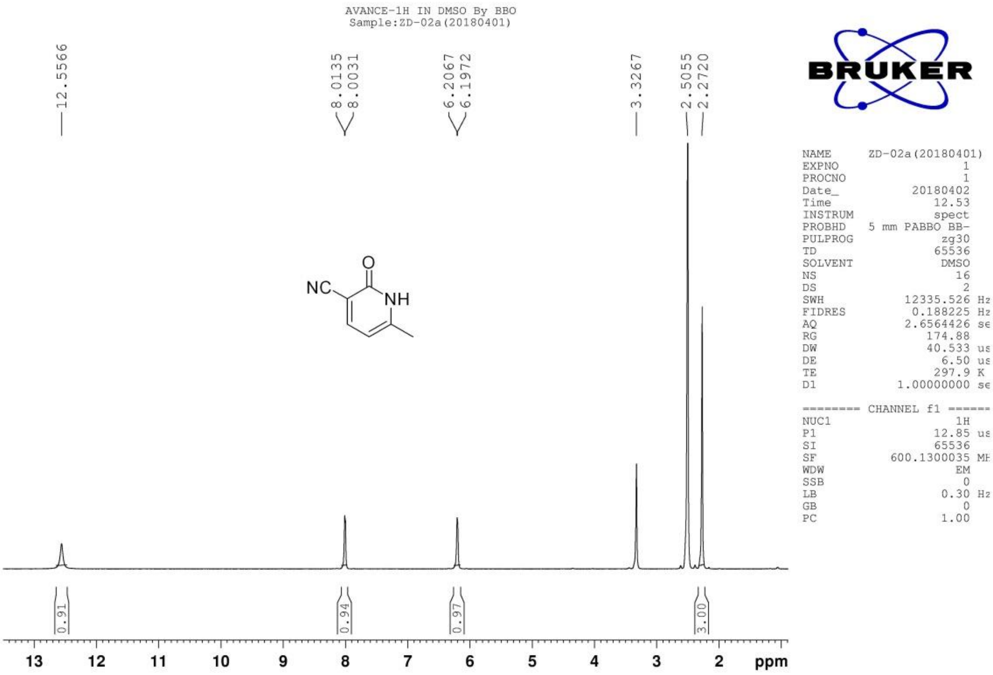

^{1}H NMR spectra of **1b**

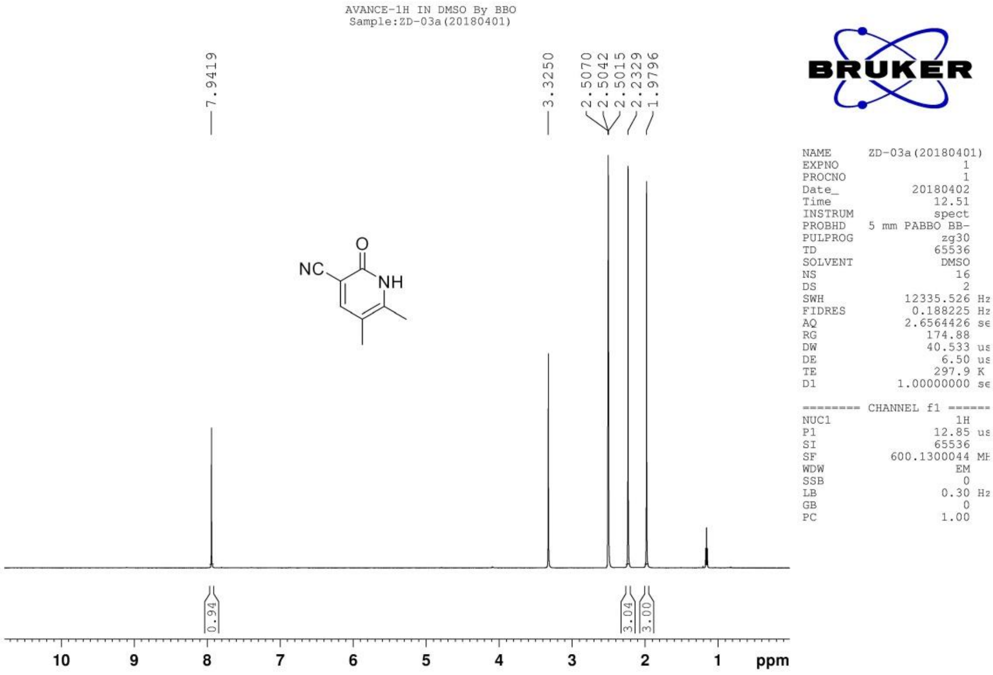

^{1}H NMR spectra of **1c**

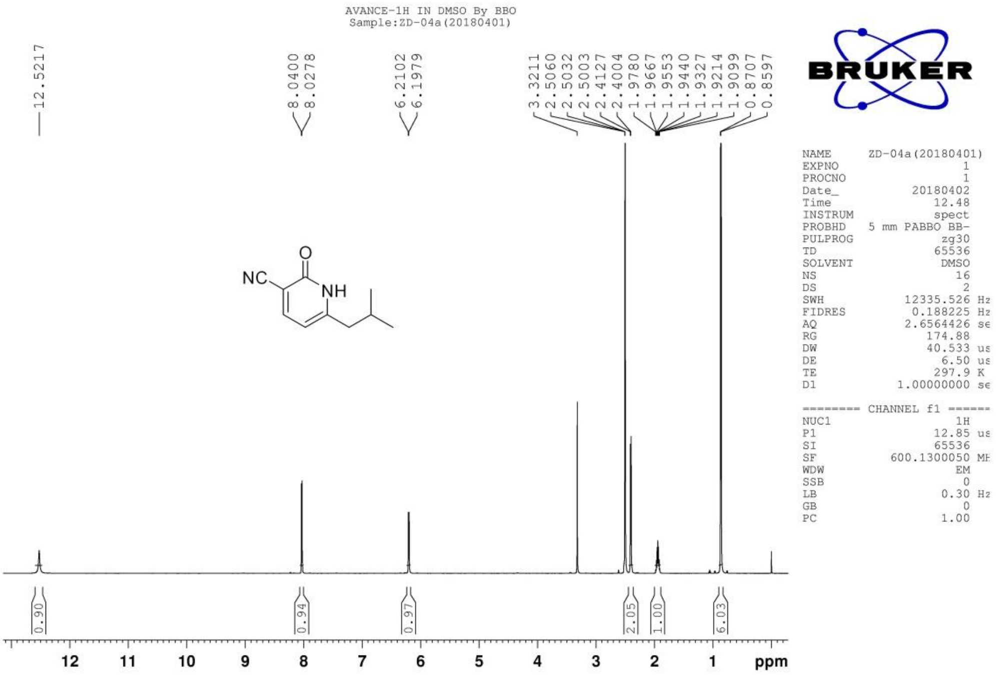

[1] H NMR spectra of **1d**

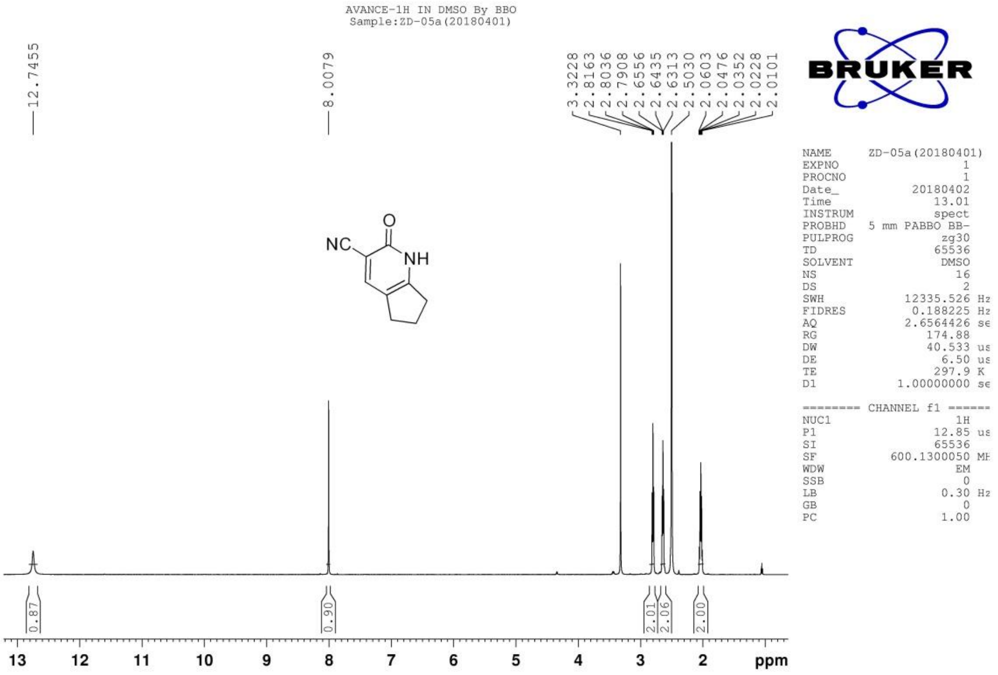

[1] H NMR spectra of **1e**

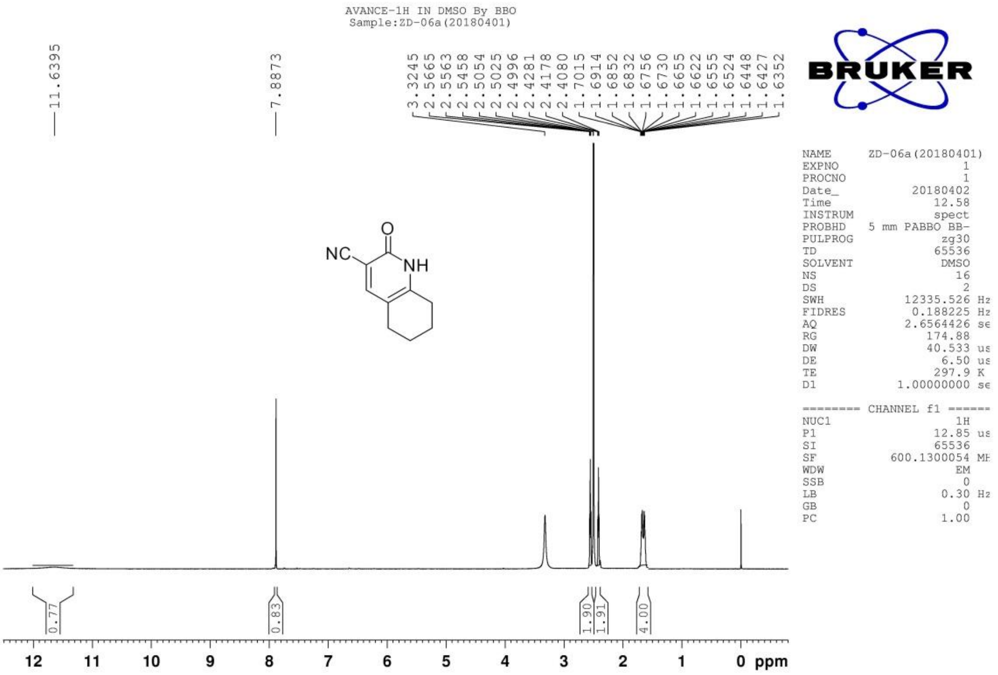

^{1}H NMR spectra of **1f**

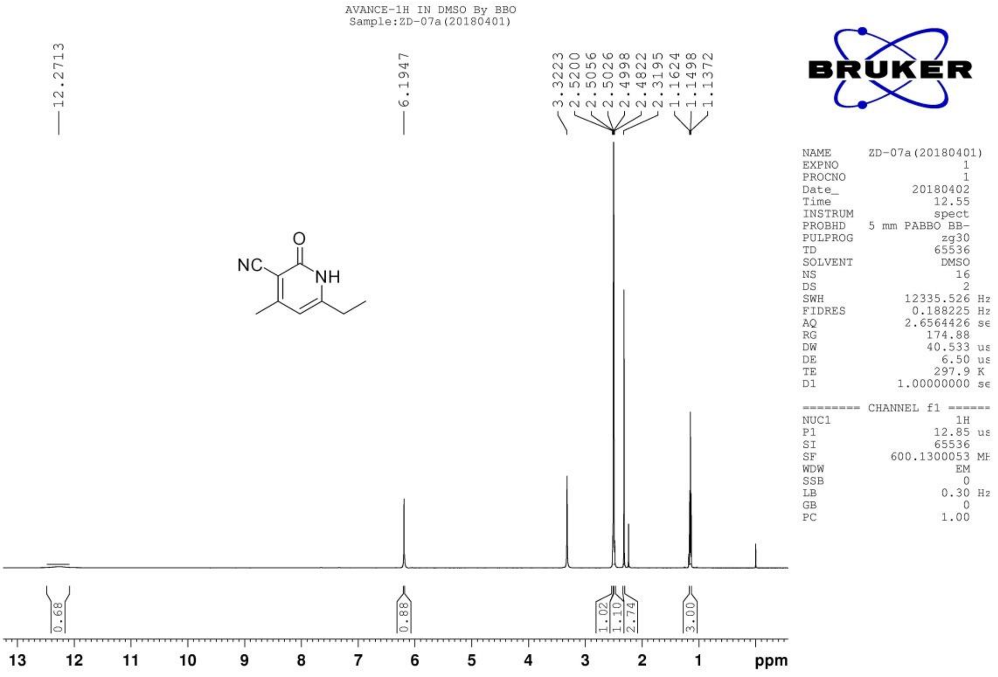

^{1}H NMR spectra of **1g**

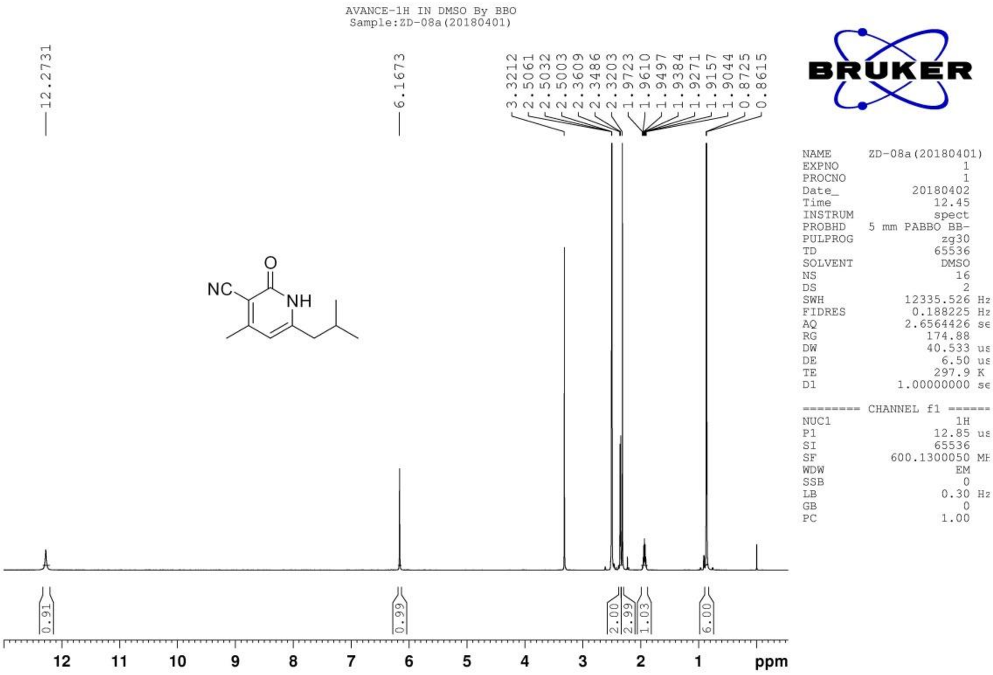

^{1}H NMR spectra of **1h**

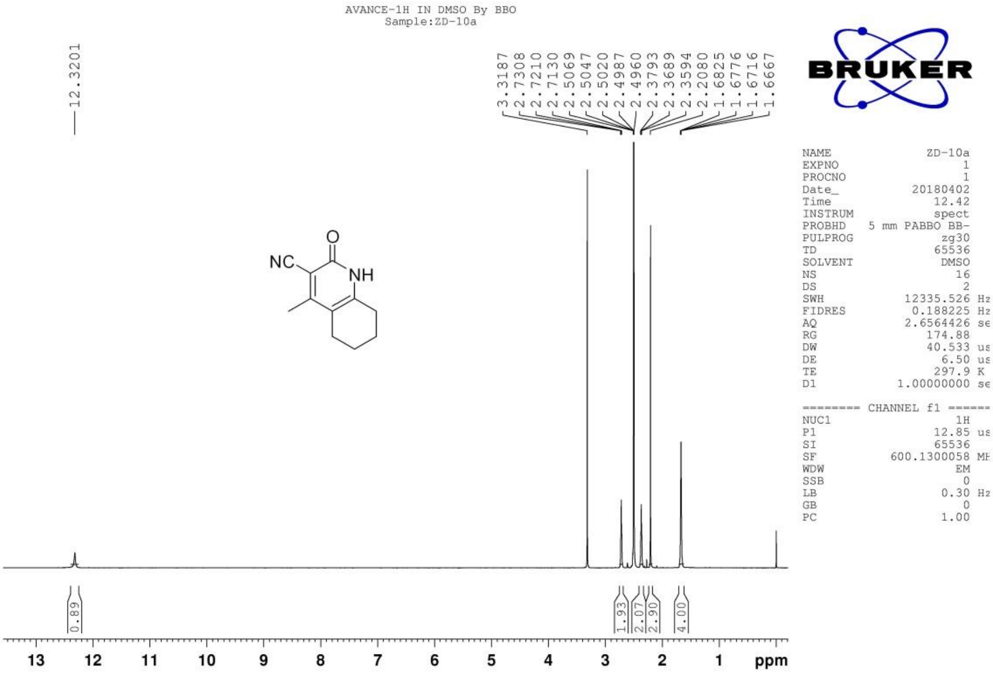

^{1}H NMR spectra of **1i**

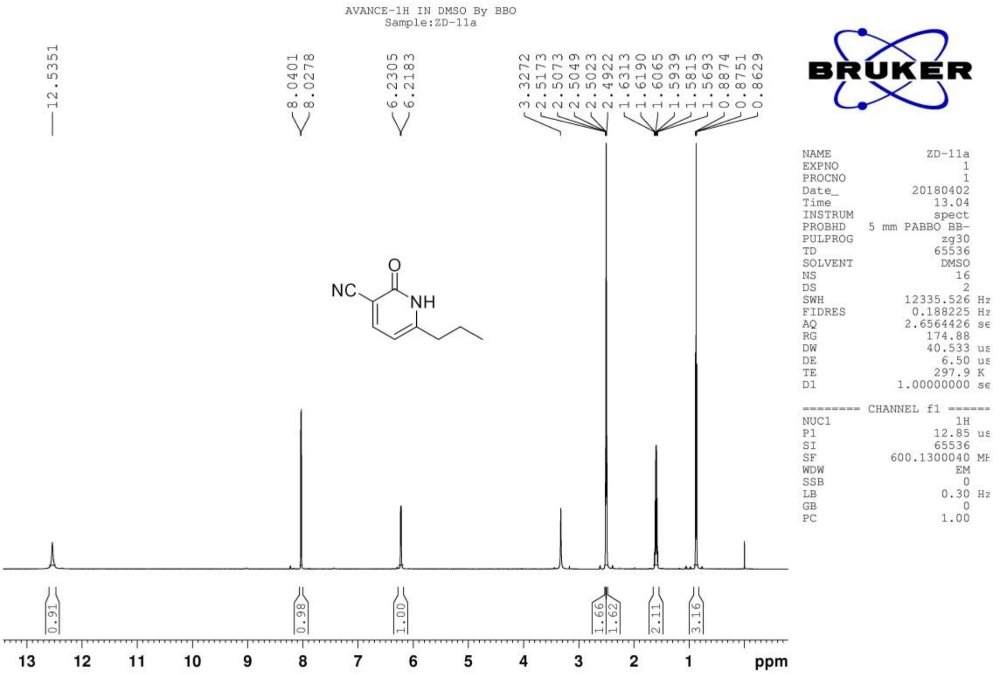

^{1}H NMR spectra of **1j**

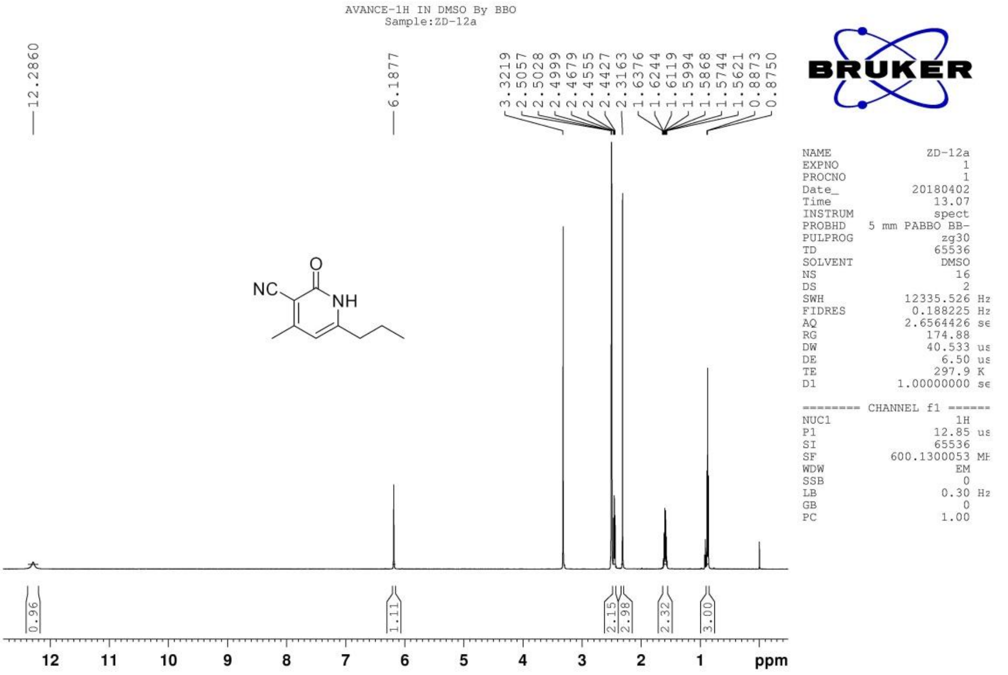

^{1}H NMR spectra of **1k**

7. ^{1}H and ^{13}C NMR spectra and HRMS spectra of 3a-3m

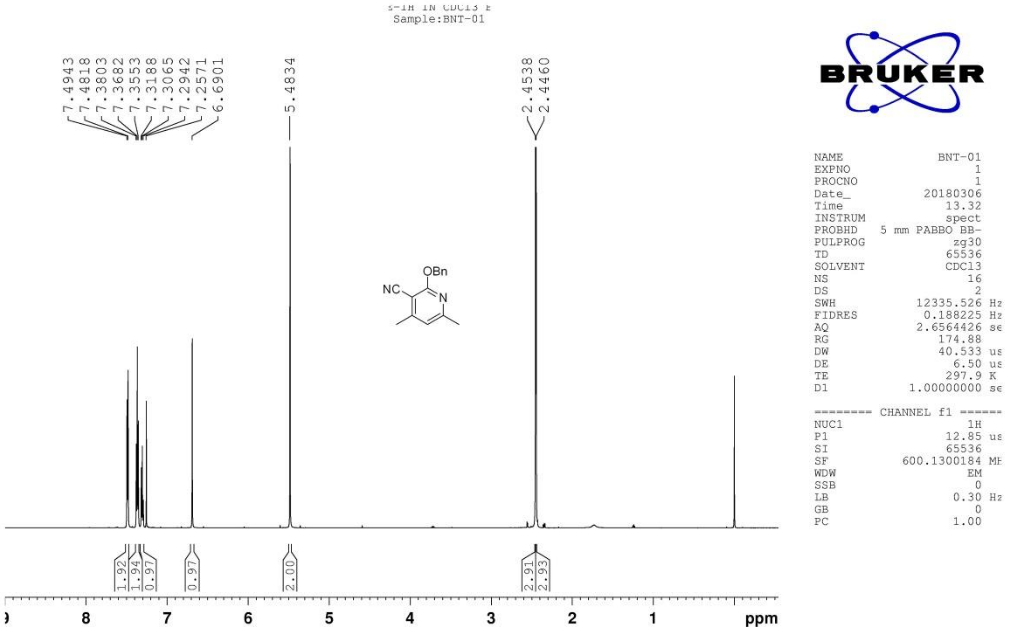

^{1}H NMR spectra of **3a**

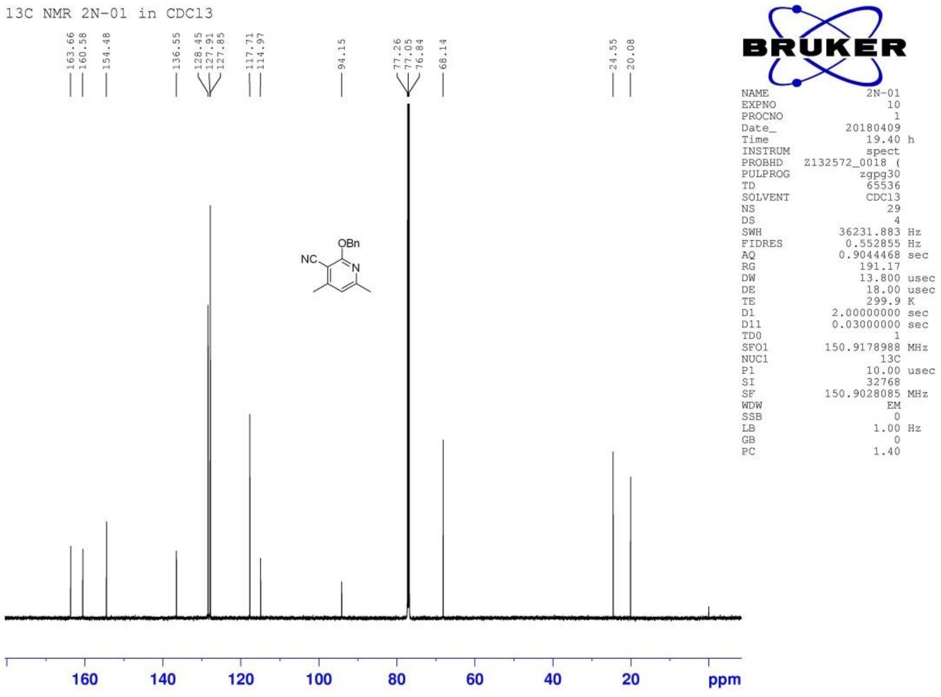

^{13}C NMR spectra of **3a**

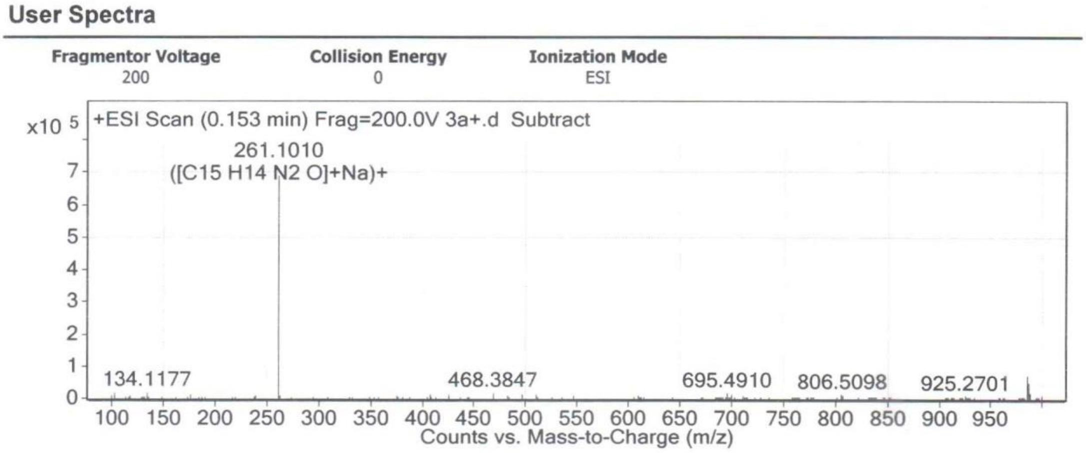

HRMS spectra of **3a**

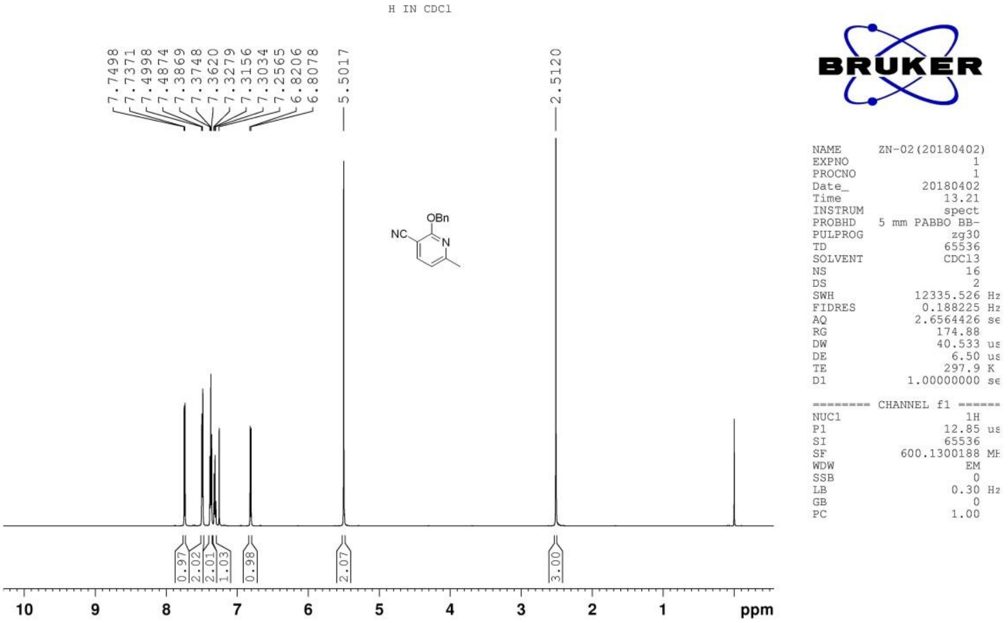

^{1}H NMR spectra of **3b**

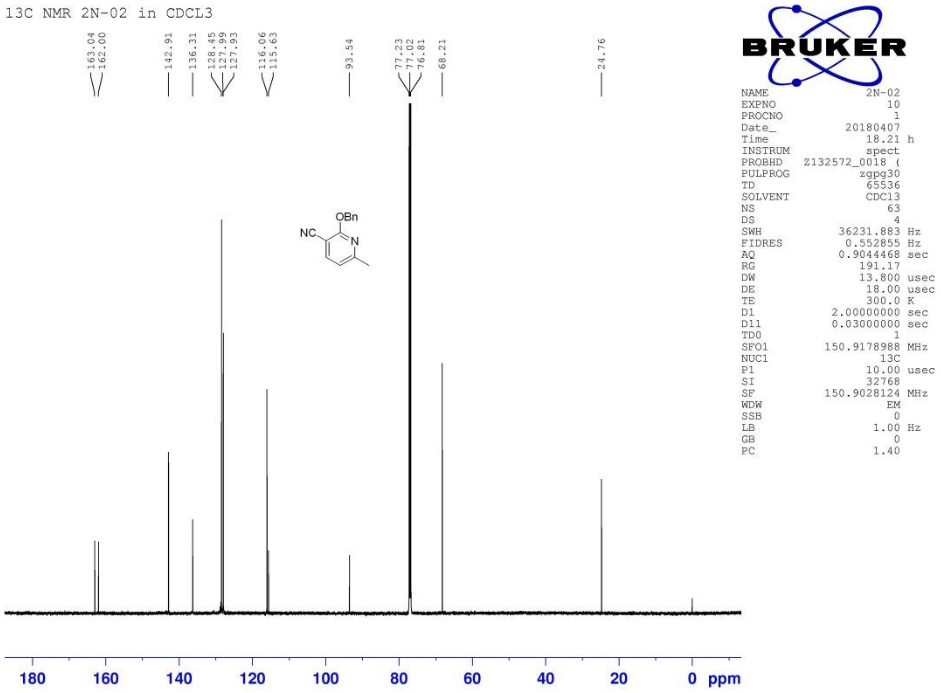

^{13}C NMR spectra of **3b**

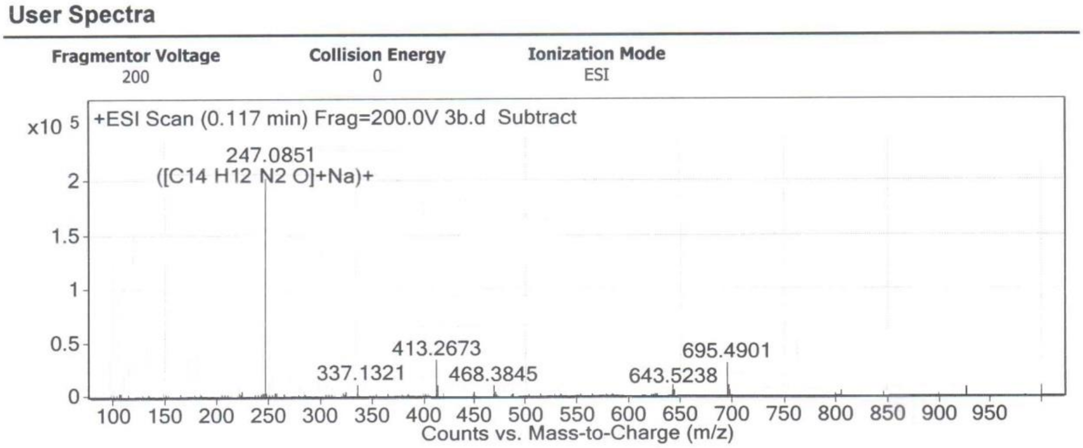

HRMS spectra of **3b**

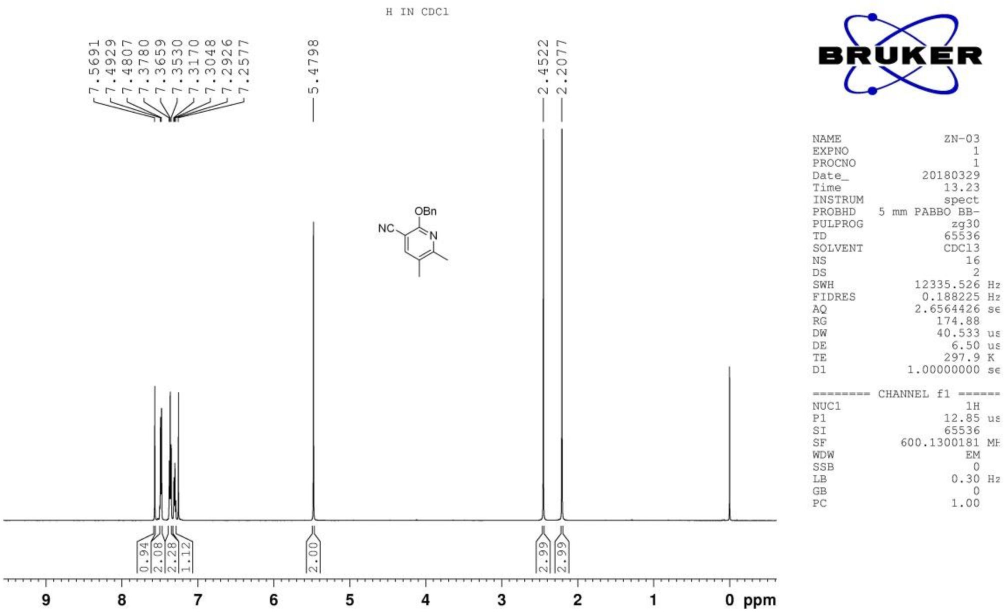

^{1}H NMR spectra of **3c**

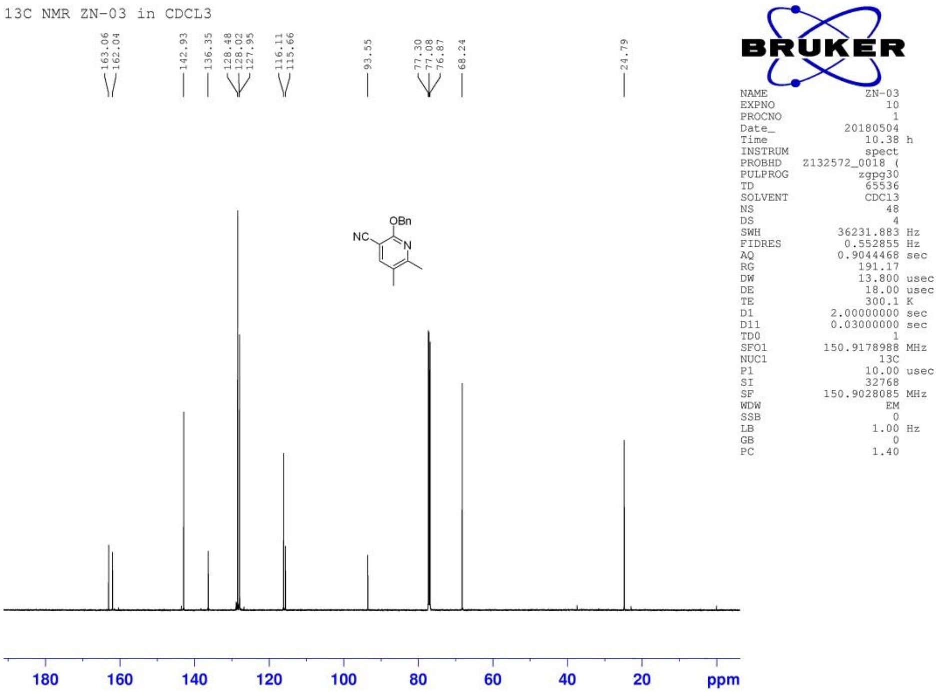

^{13}C NMR spectra of **3c**

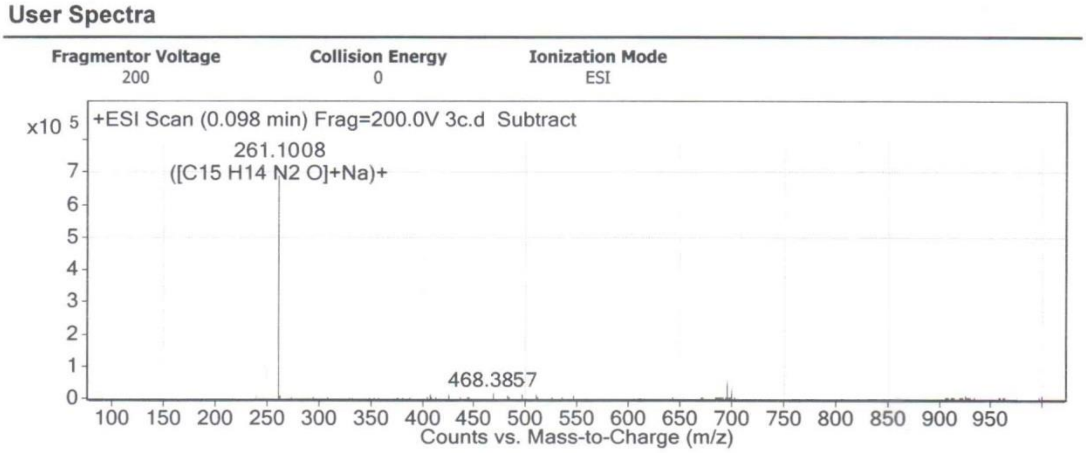

HRMS spectra of **3c**

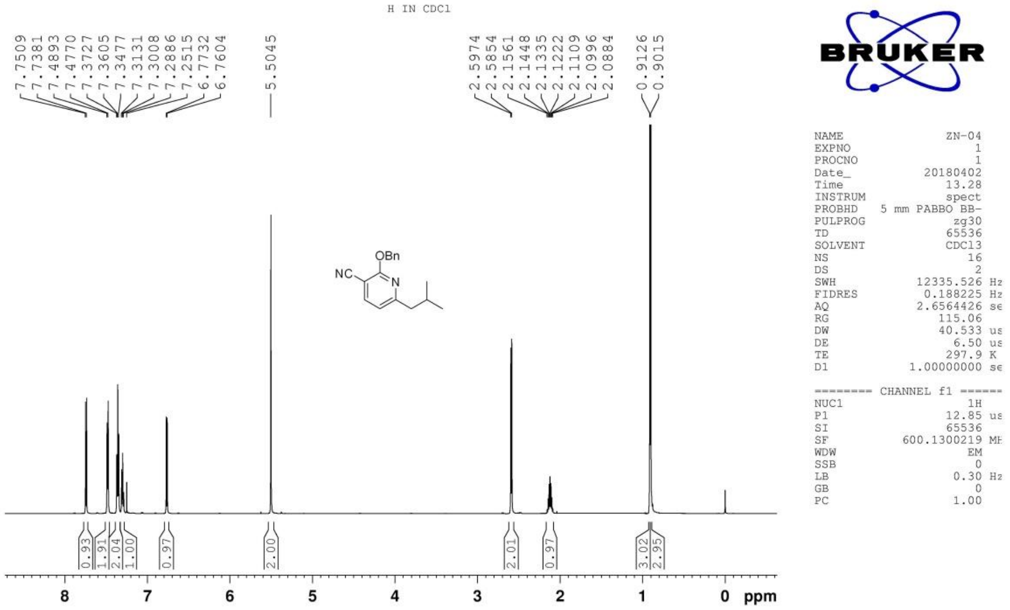

^{1}H NMR spectra of **3d**

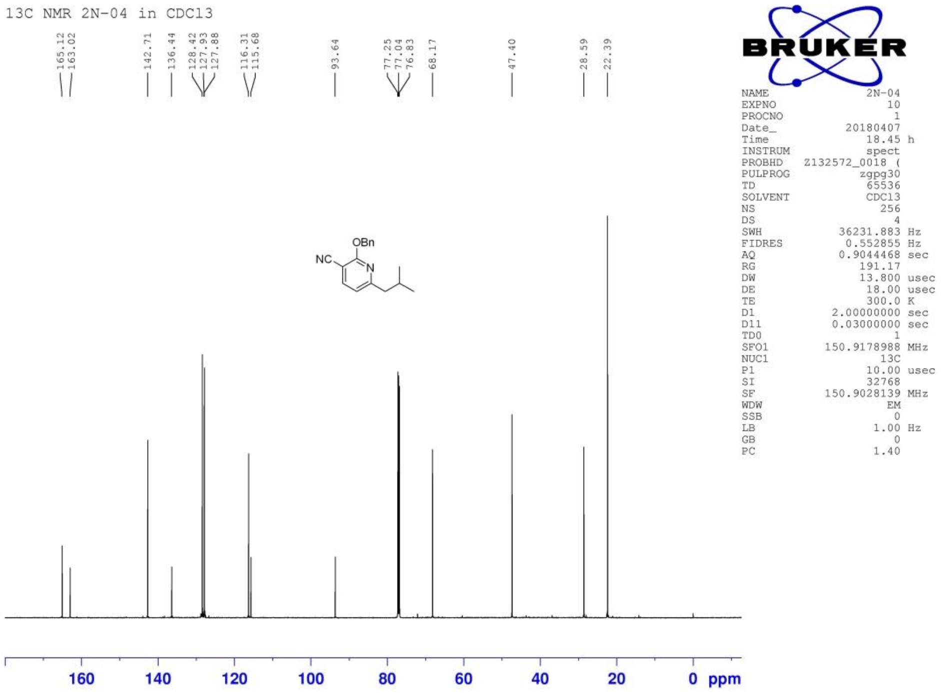

^{13}C NMR spectra of **3d**

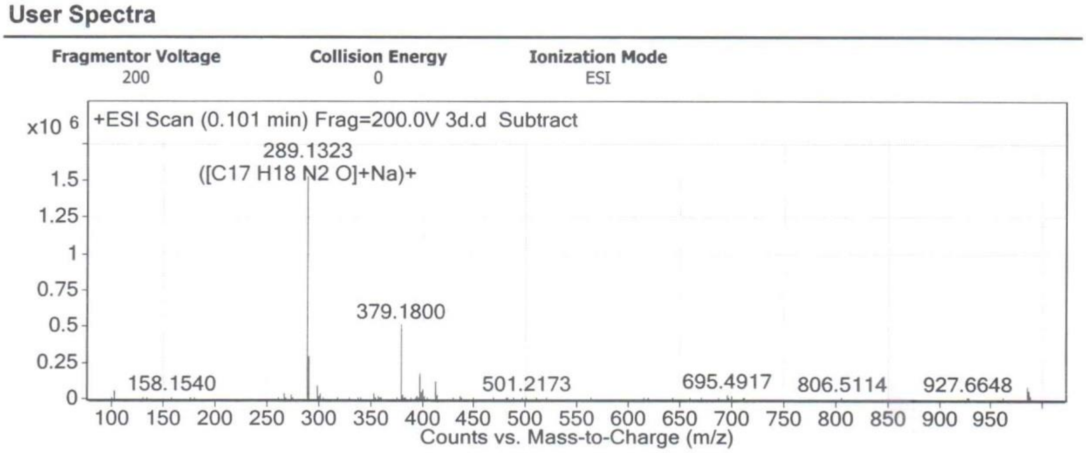

HRMS spectra of **3d**

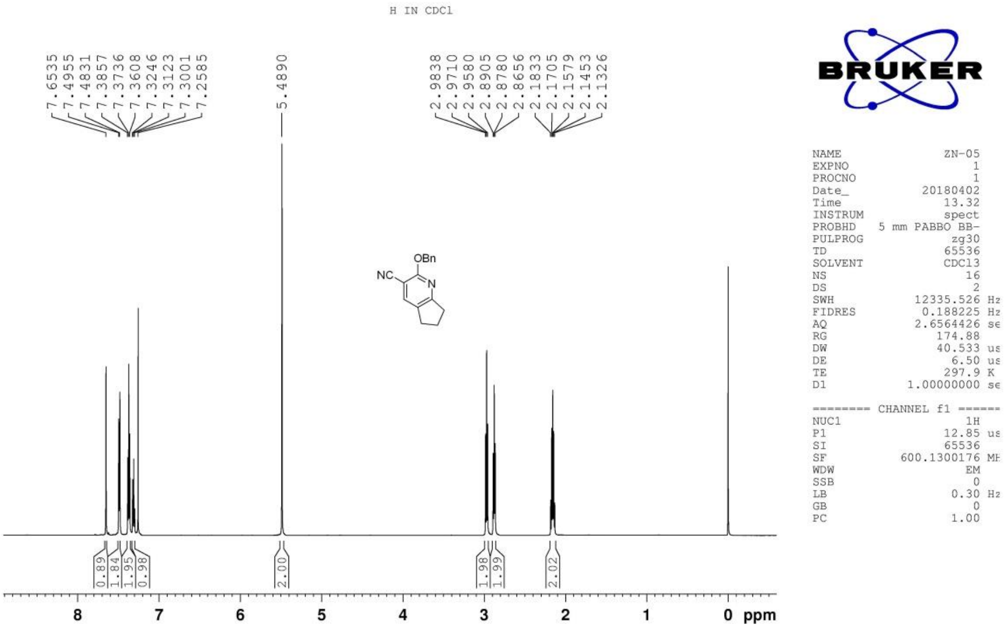

^{1}H NMR spectra of **3e**

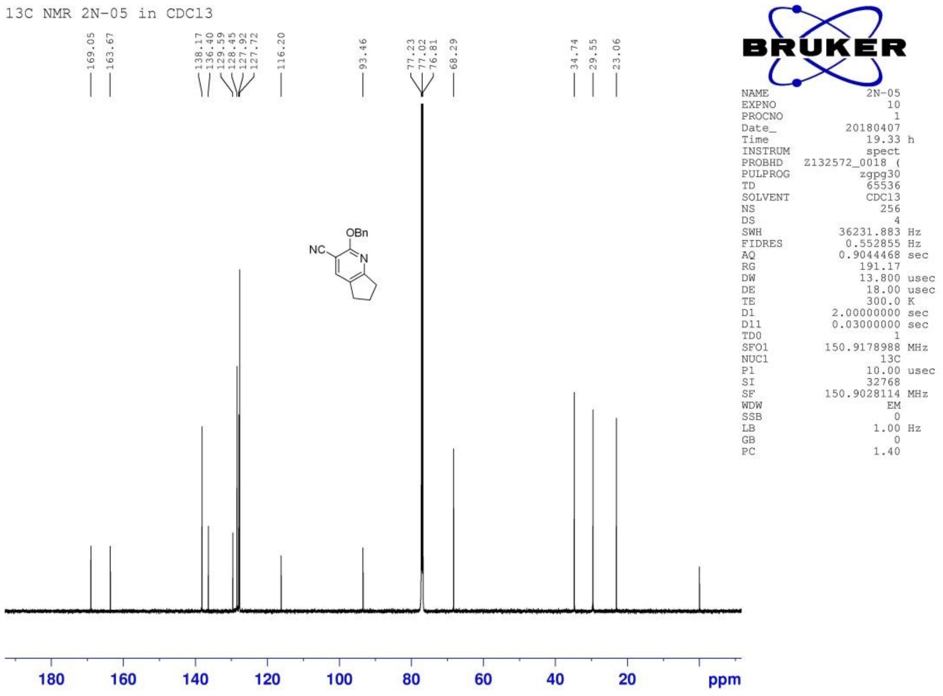

^{13}C NMR spectra of **3e**

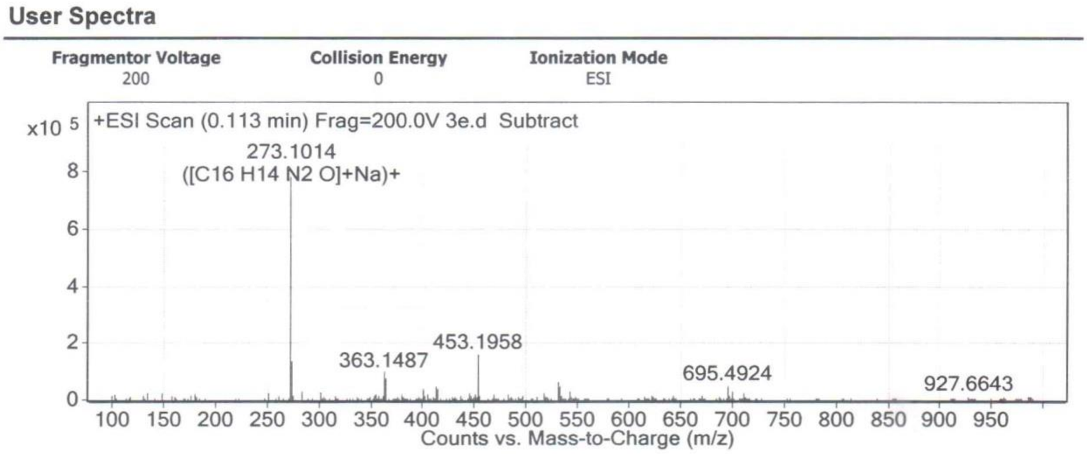

HRMS spectra of **3e**

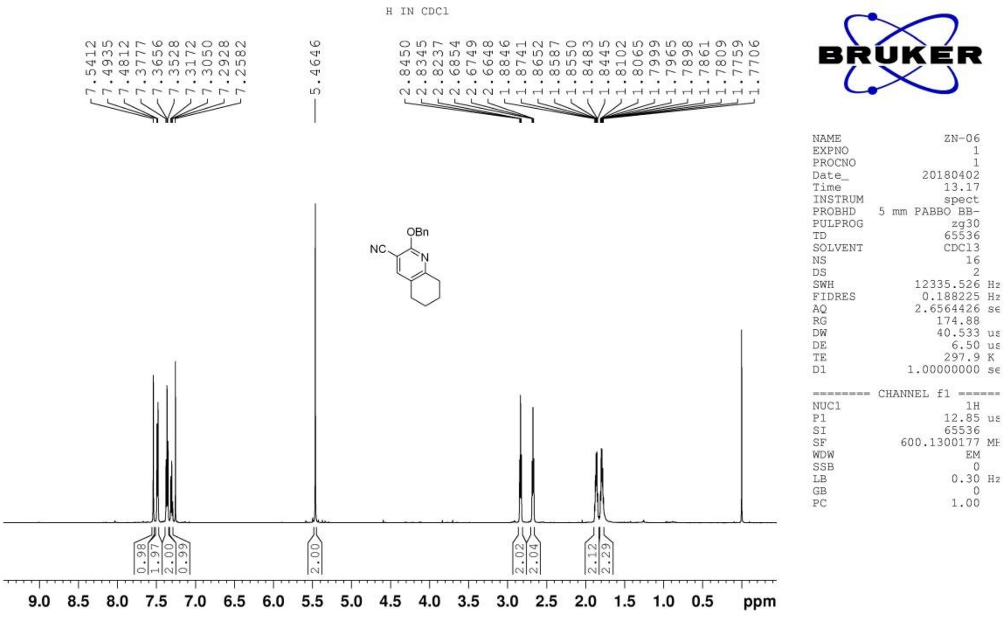

^{1}H NMR spectra of **3f**

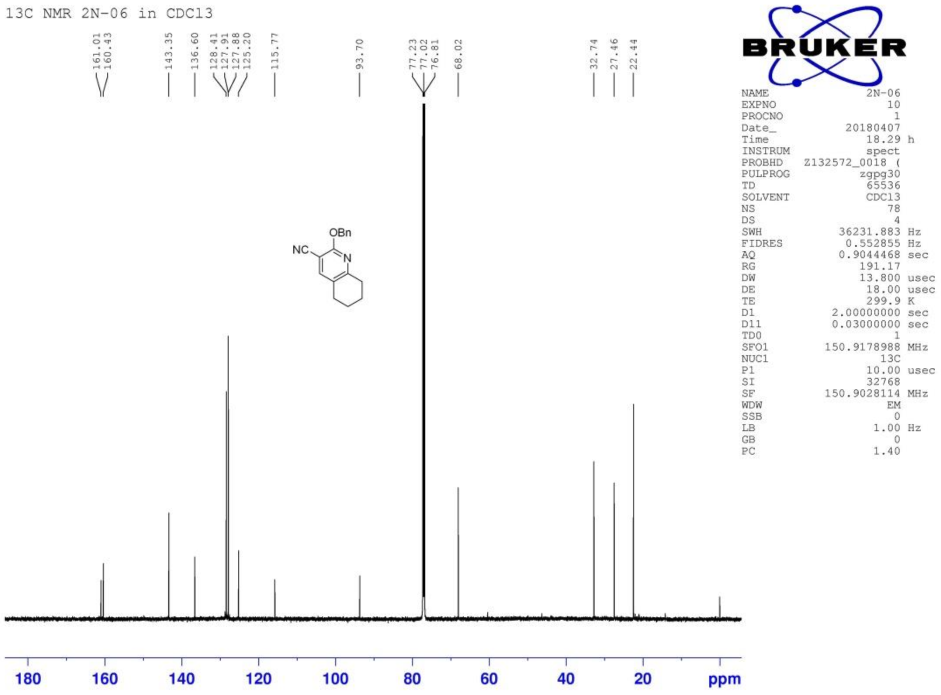

^{13}C NMR spectra of **3f**

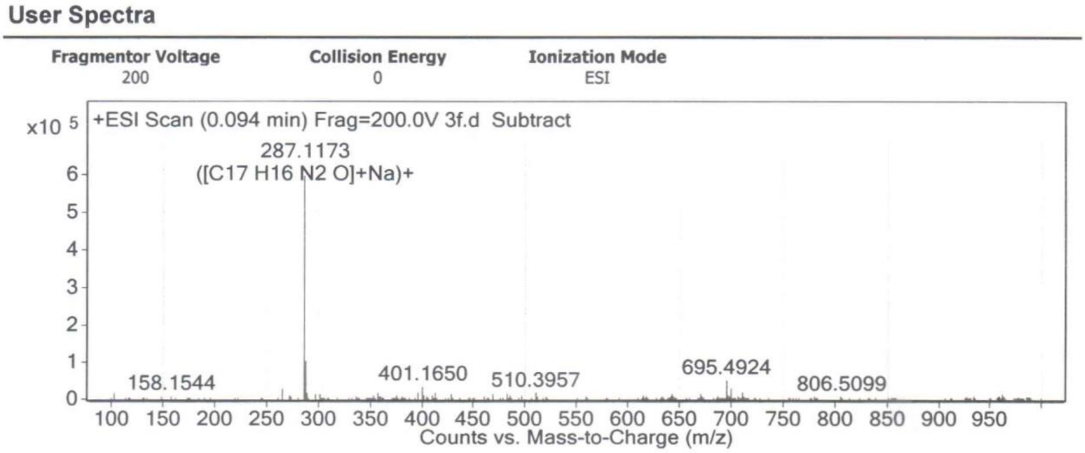

HRMS spectra of **3f**

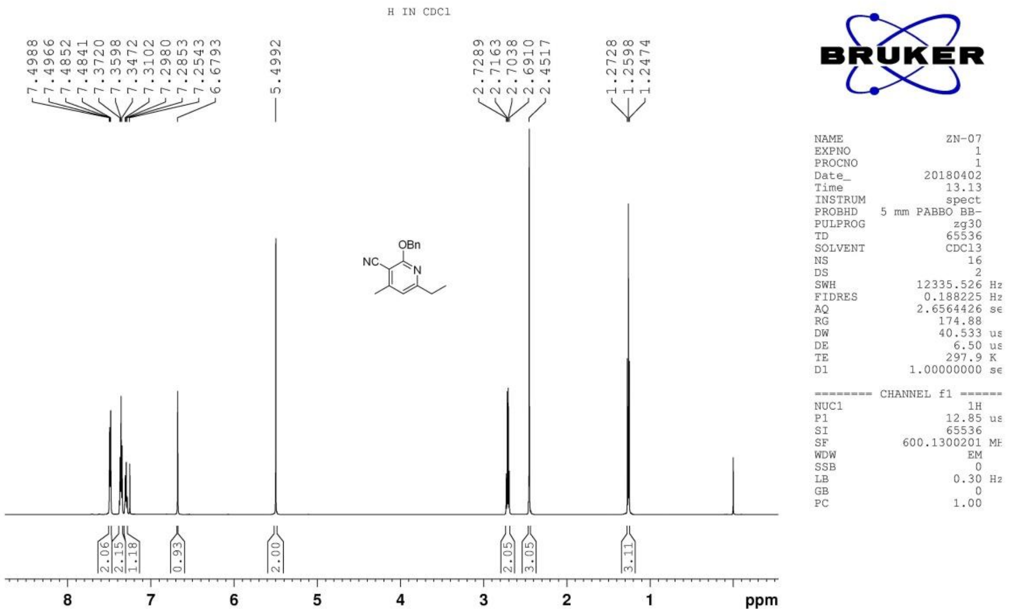

^{1}H NMR spectra of **3g**

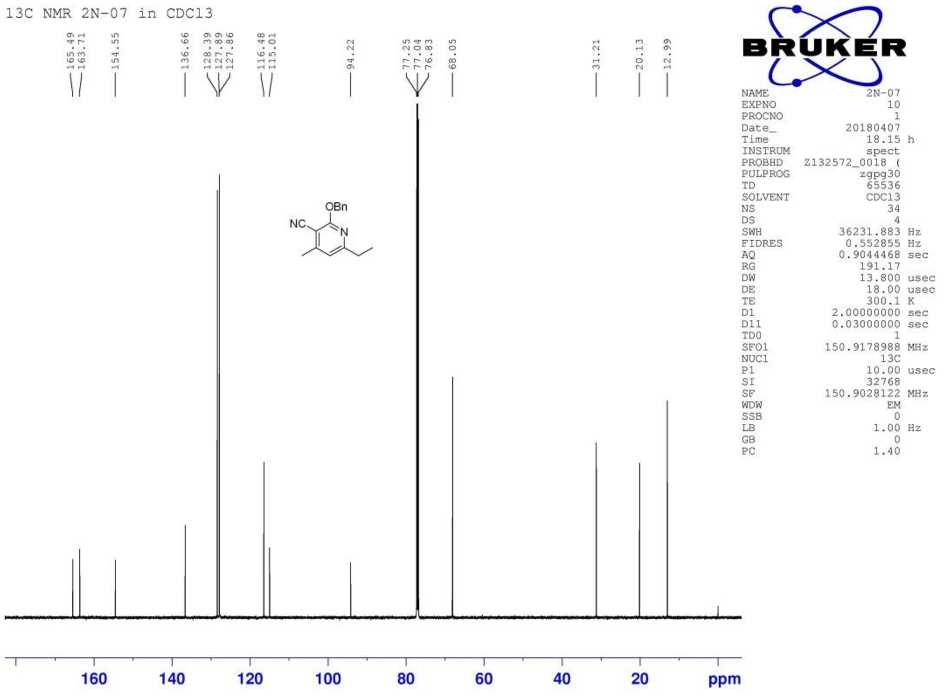

^{13}C NMR spectra of **3g**

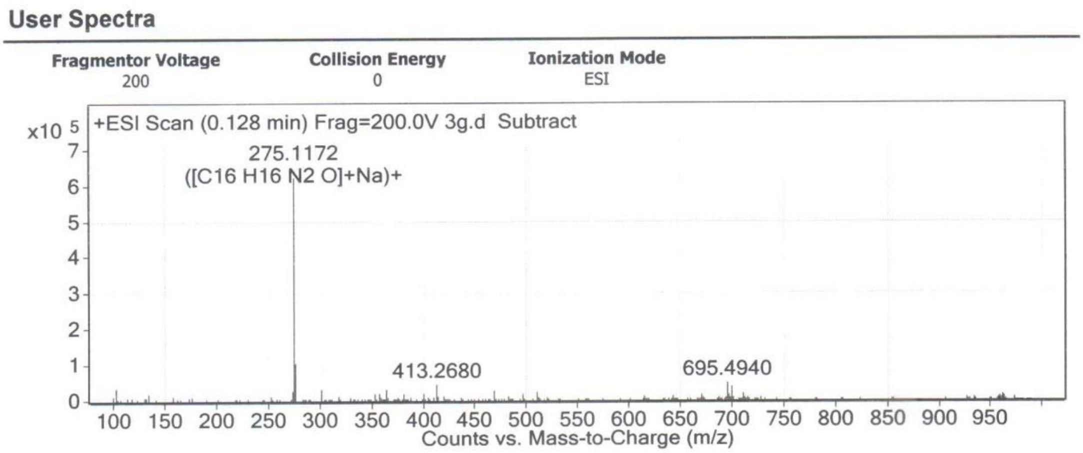

HRMS spectra of **3g**

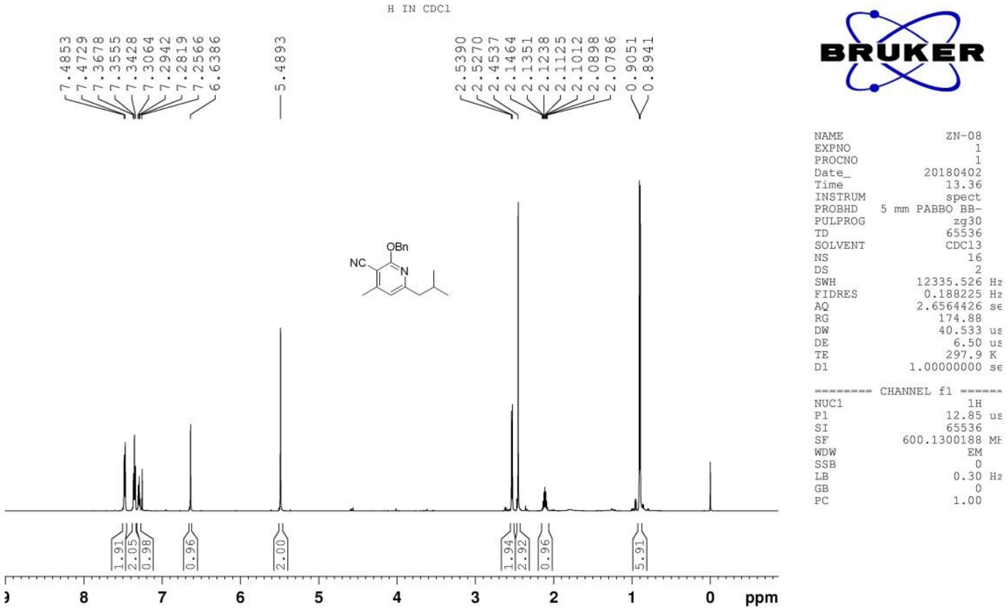

^{1}H NMR spectra of **3h**

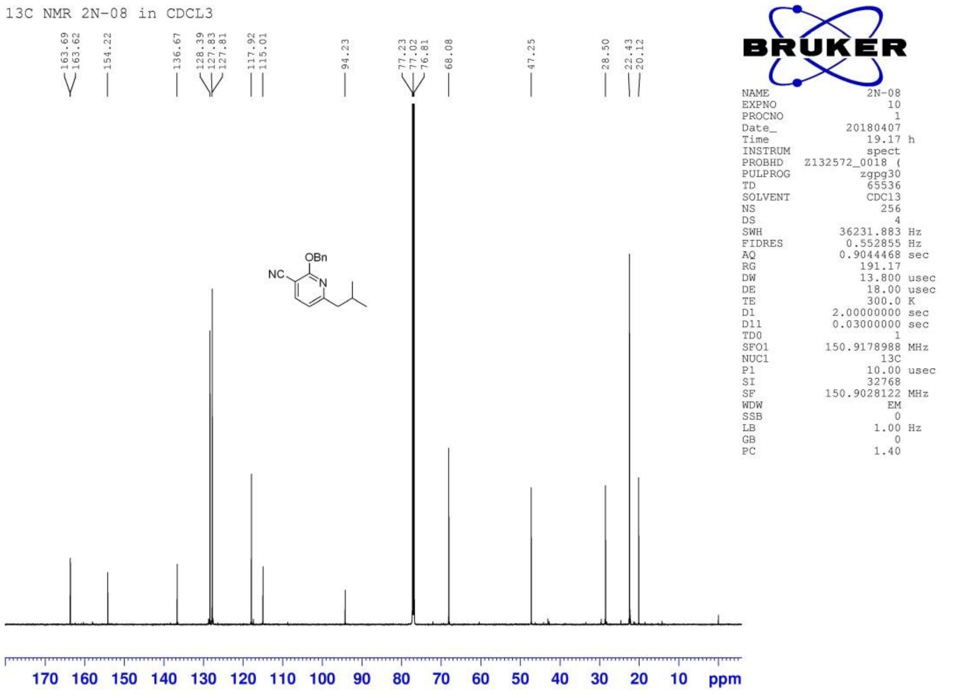

^{13}C NMR spectra of **3h**

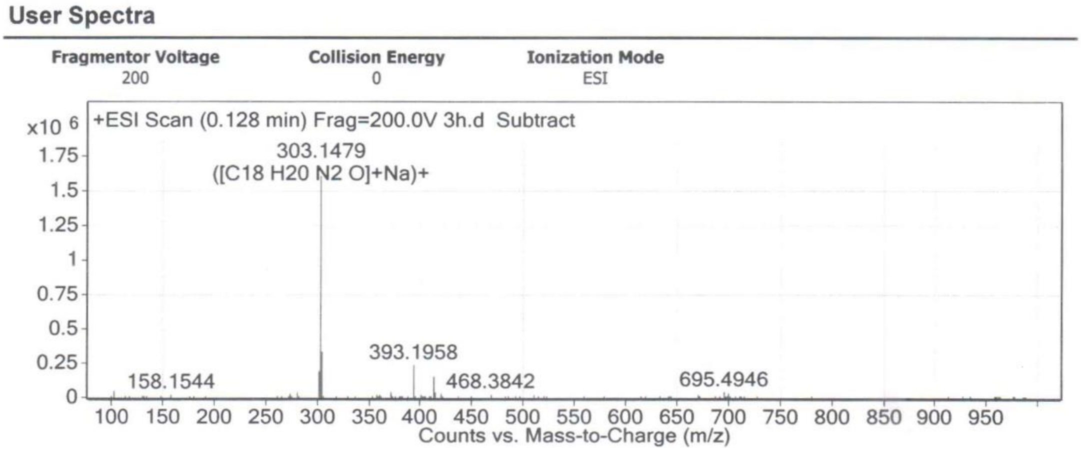

HRMS spectra of **3h**

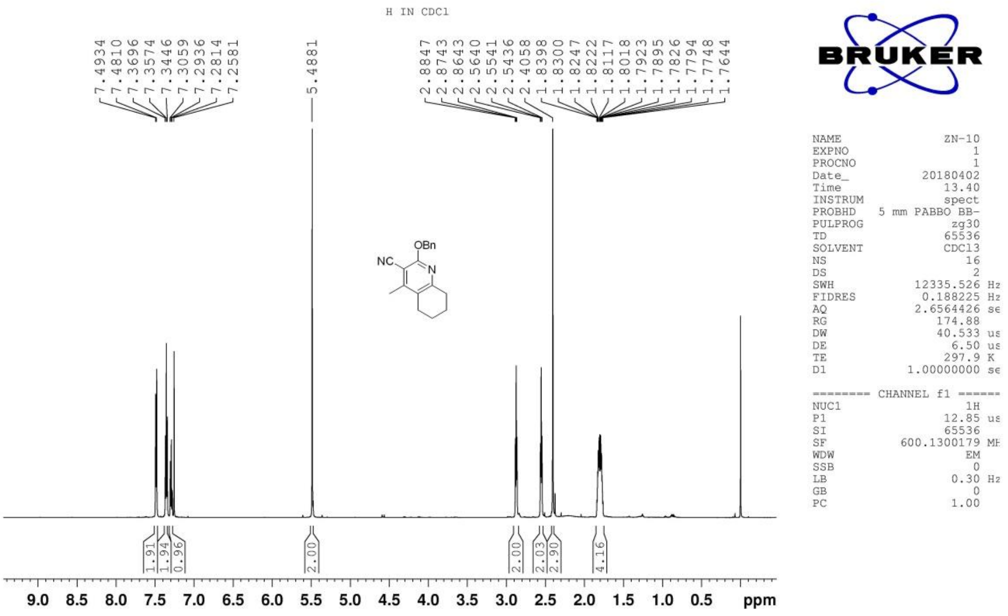

^{1}H NMR spectra of **3i**

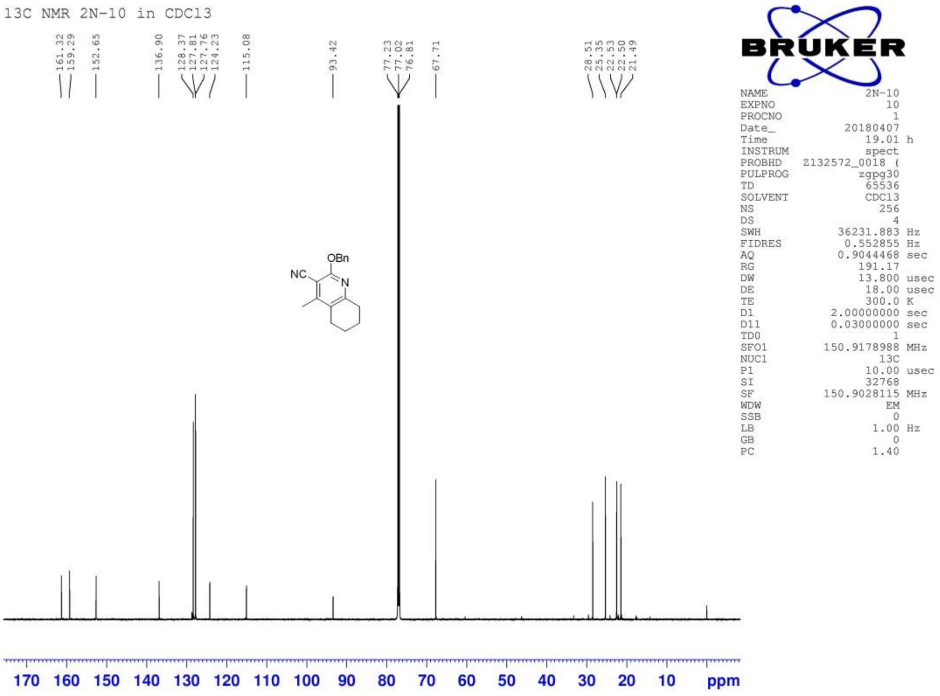

^{13}C NMR spectra of **3i**

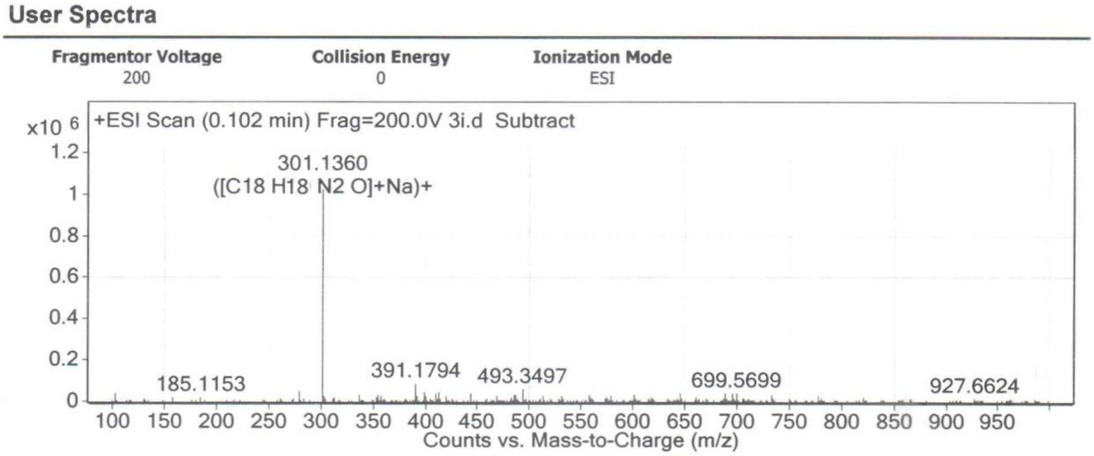

HRMS spectra of **3i**

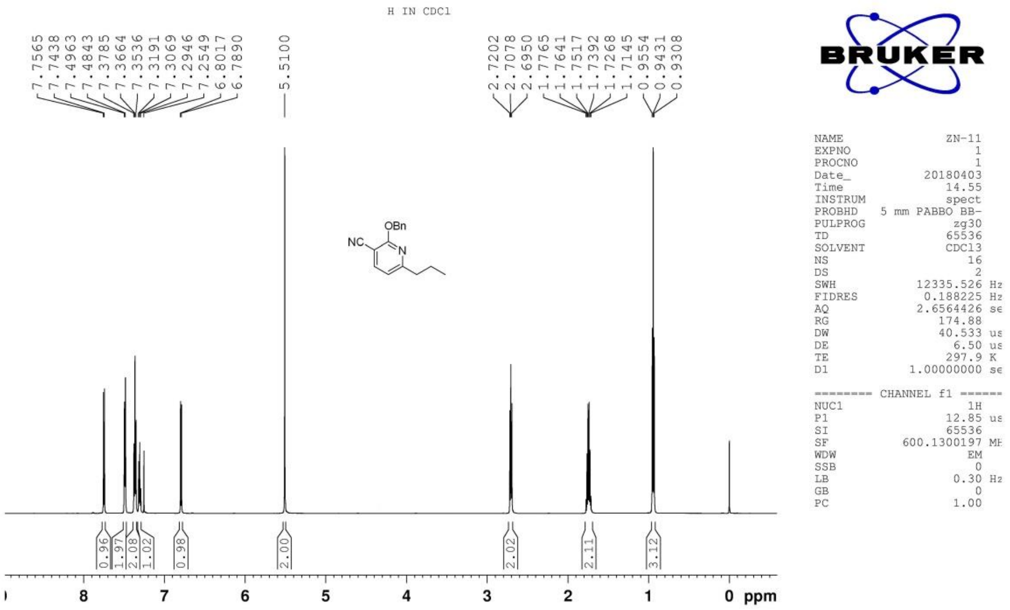

^{1}H NMR spectra of **3j**

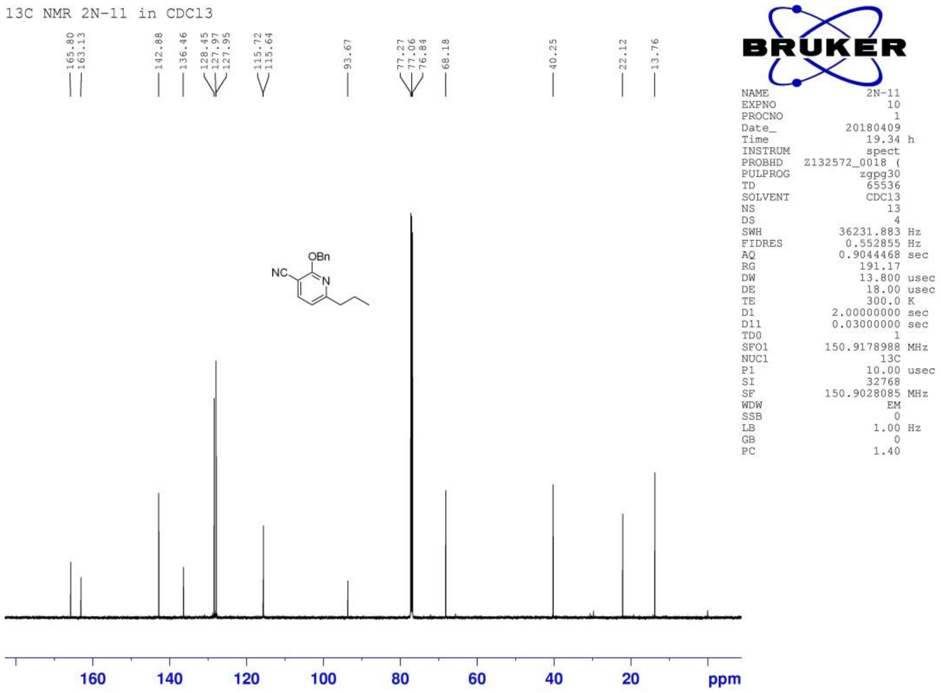

^{13}C NMR spectra of **3j**

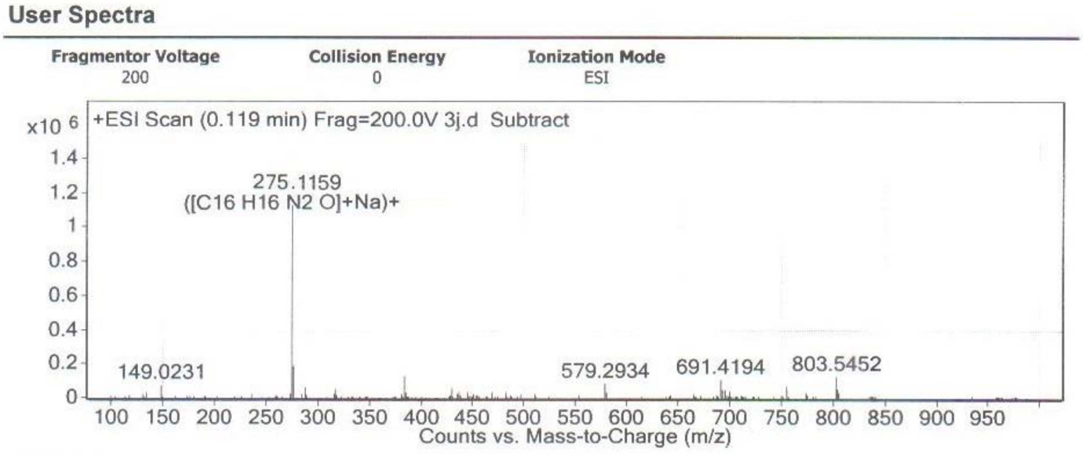

HRMS spectra of **3j**

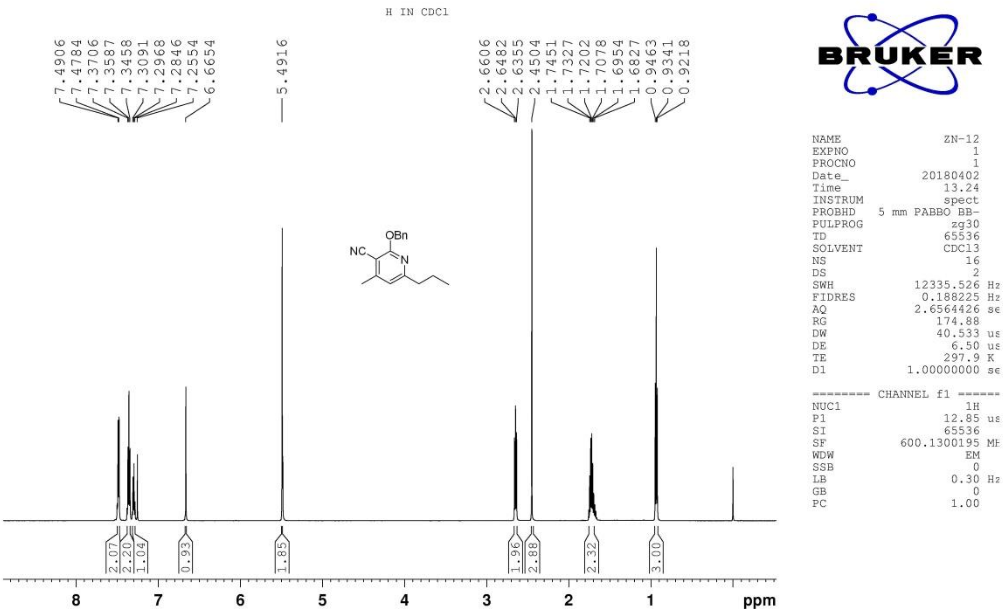

^{1}H NMR spectra of **3k**

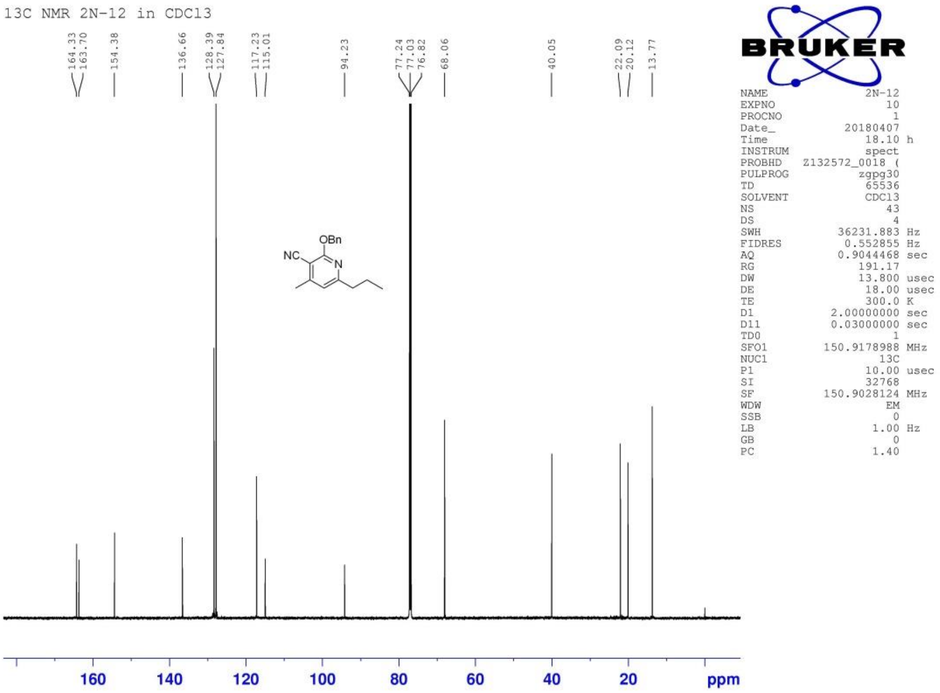

^{13}C NMR spectra of **3k**

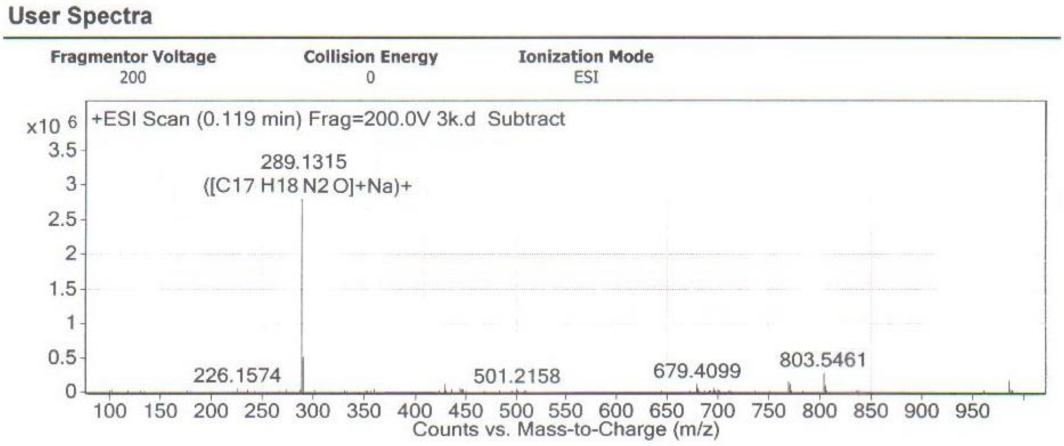

HRMS spectra of **3k**

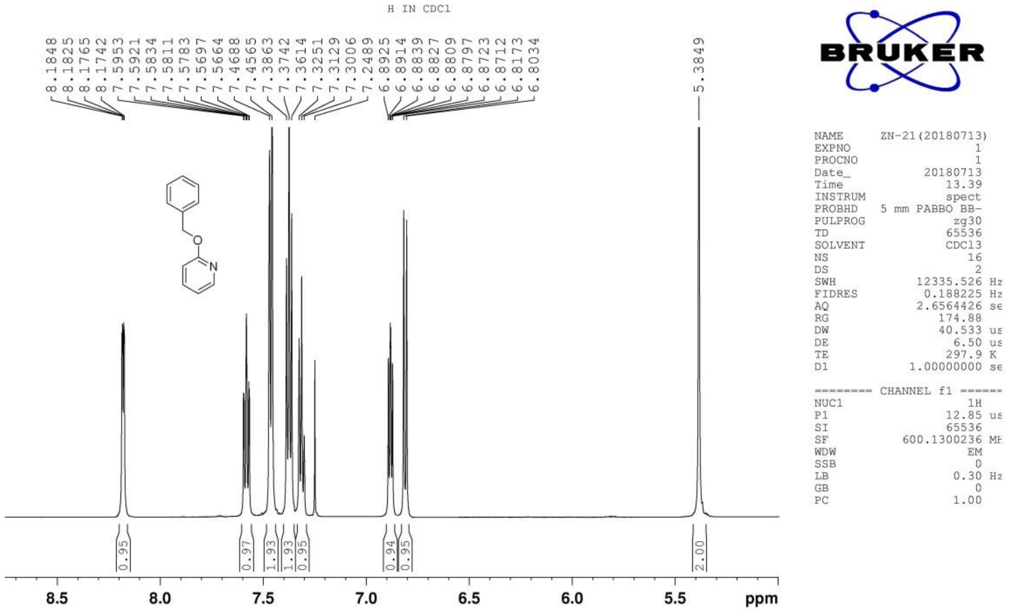

^{1}H NMR spectra of **3l**

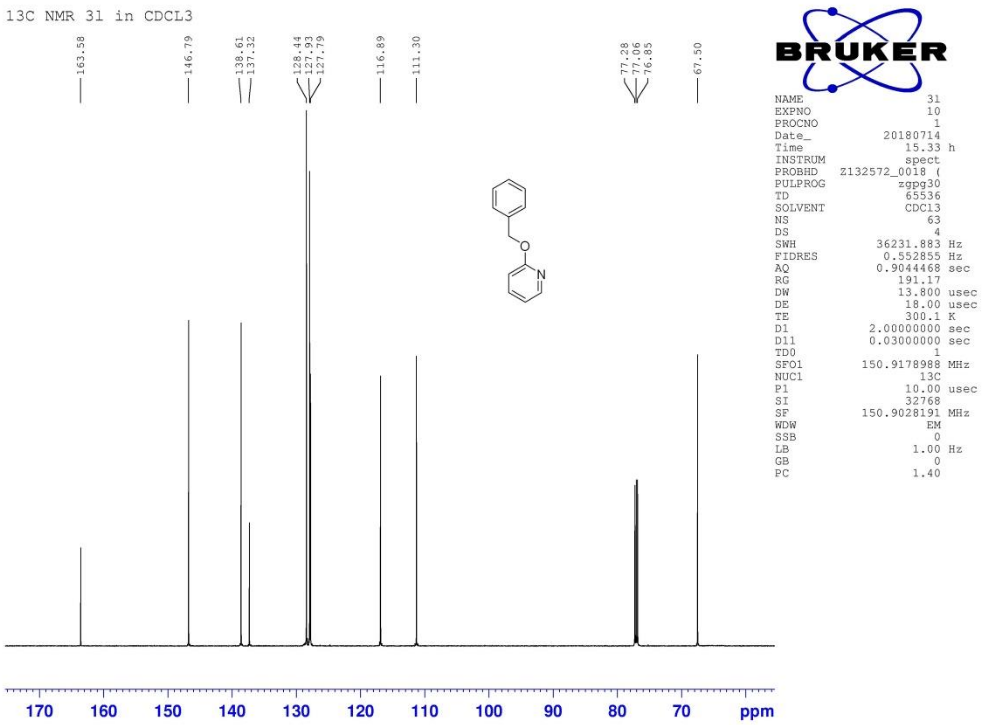

^{13}C NMR spectra of **3l**

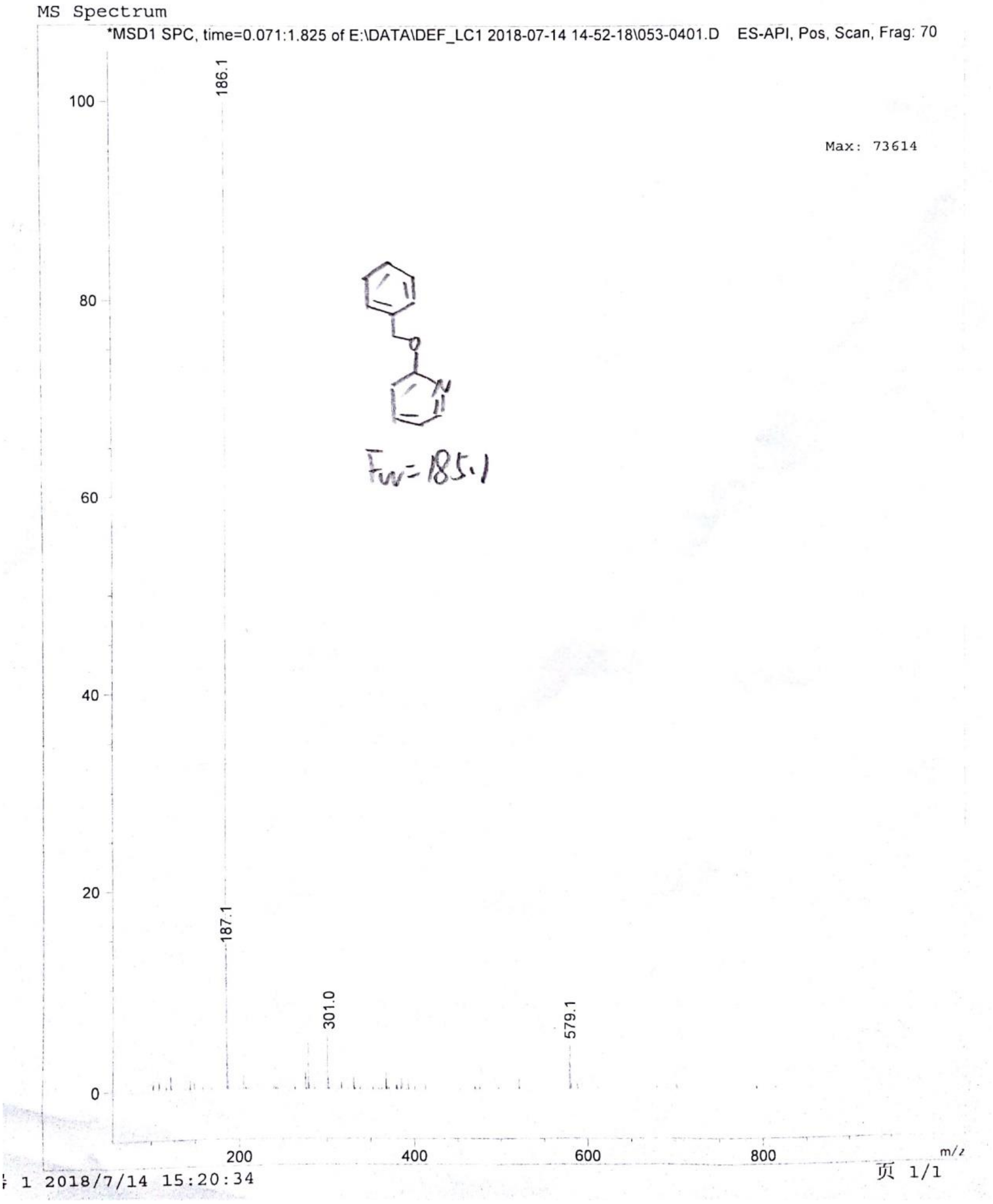

MS spectra of **3l**

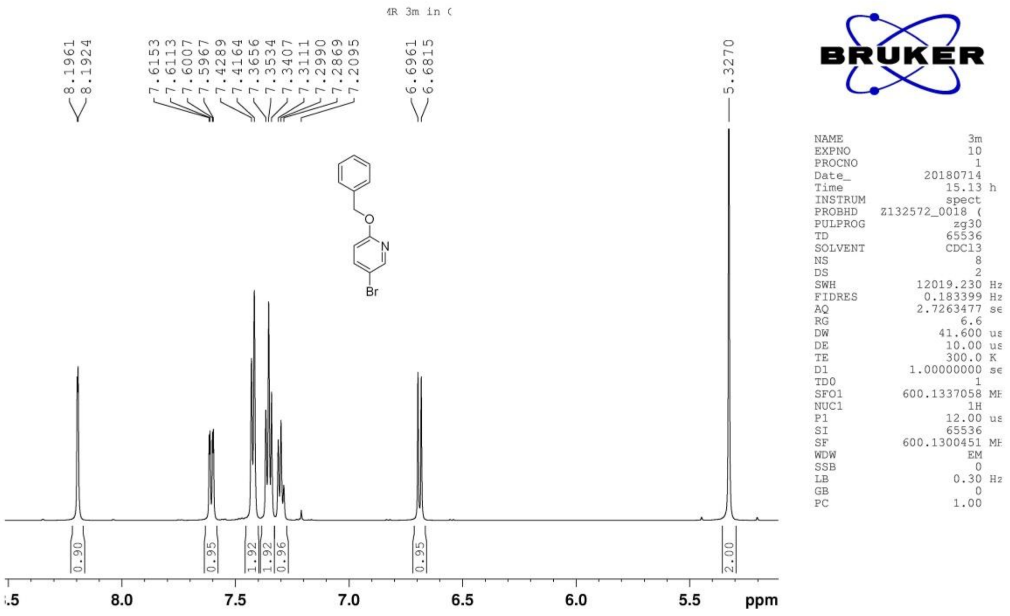

^{1}H NMR spectra of **3m**

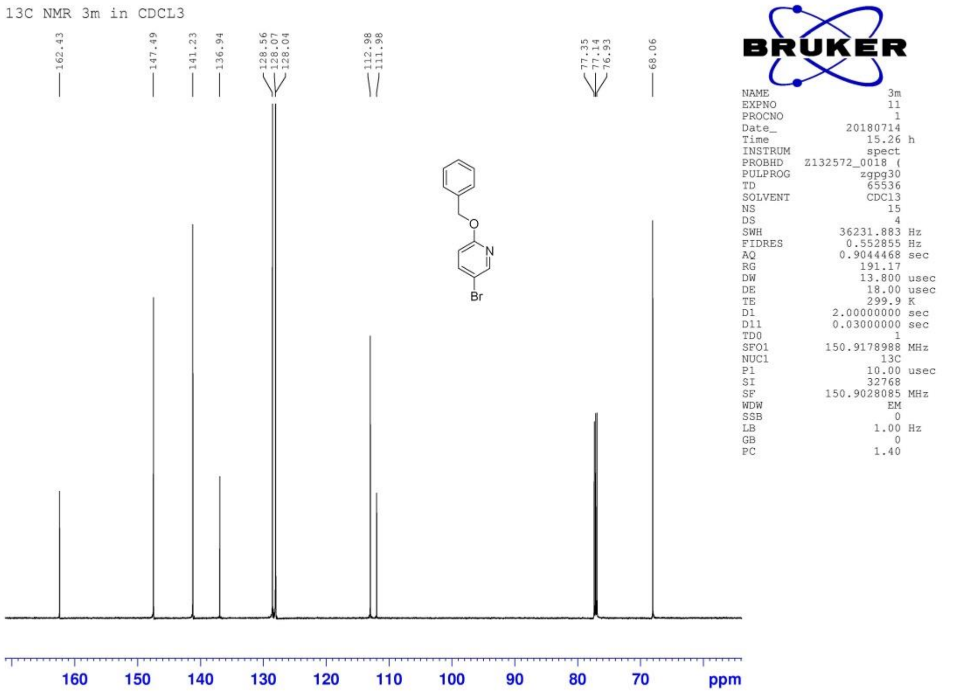

^{13}C NMR spectra of **3m**

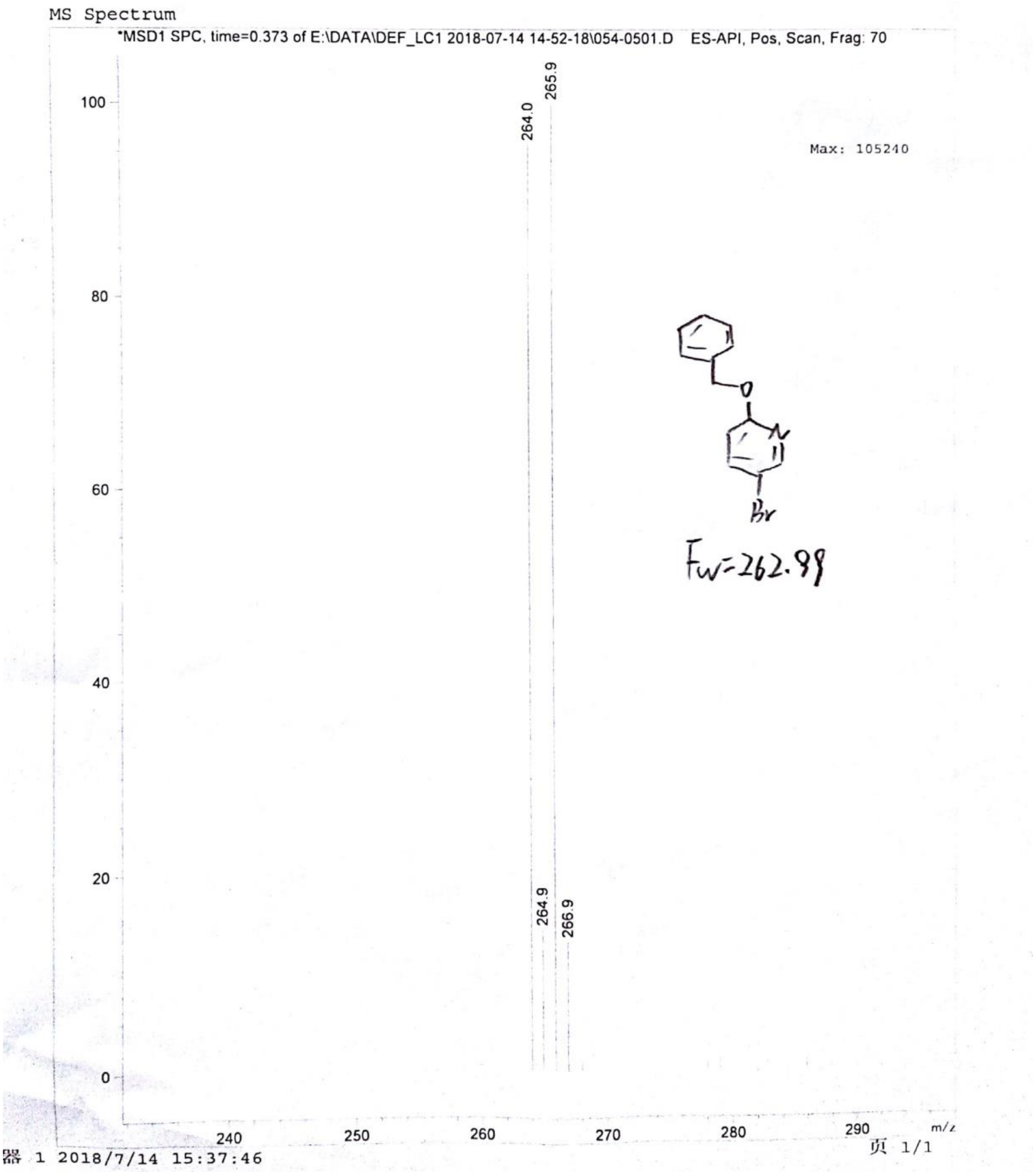

MS spectra of **3l**

8. ^{1}H and ^{13}C NMR spectra and HRMS spectra of 4a-4k

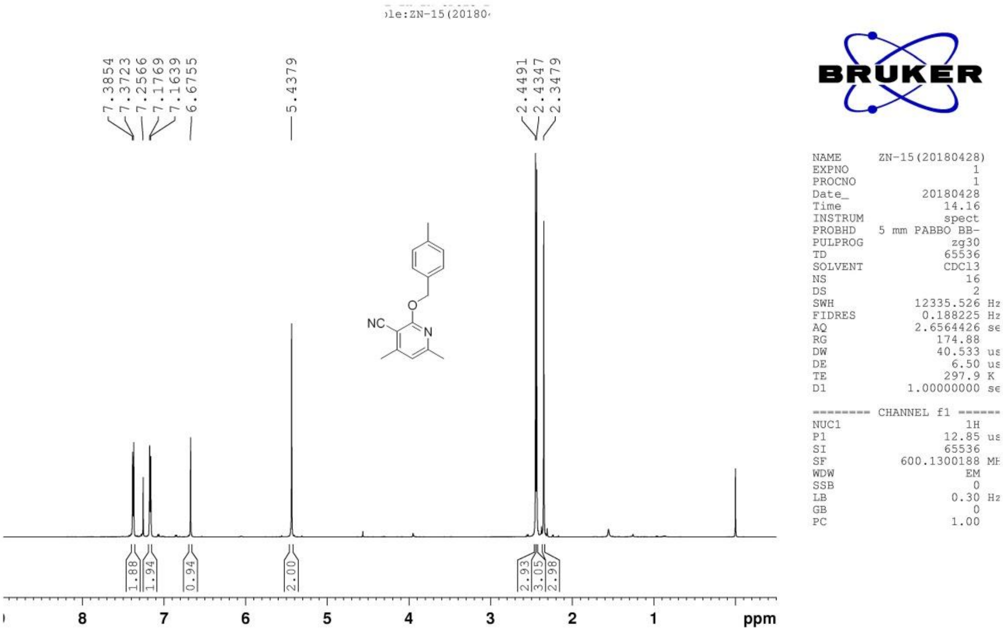

^{1}H NMR spectra of **4a**

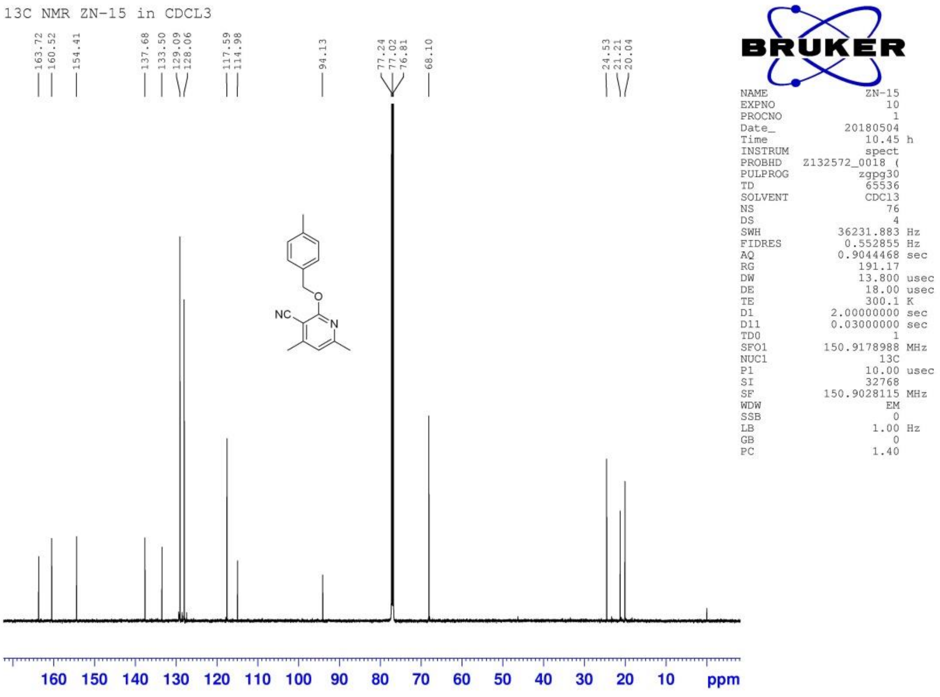

^{13}C NMR spectra of **4a**

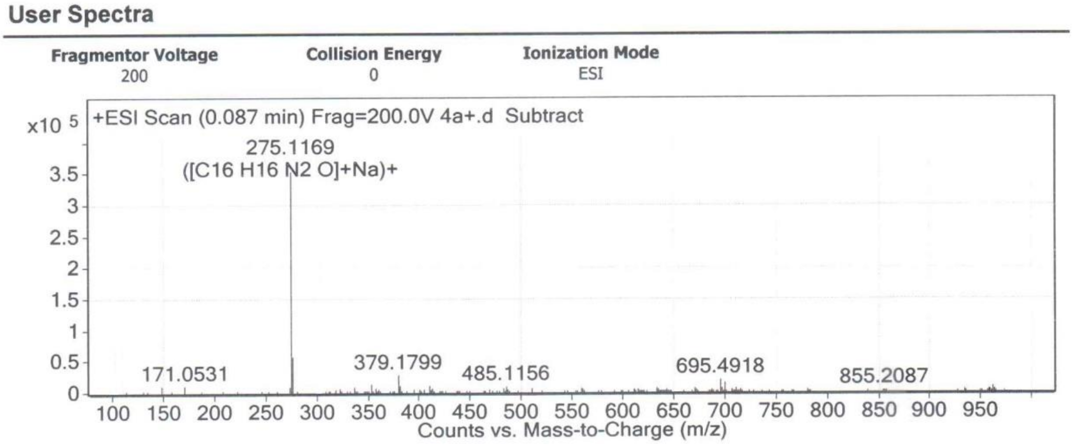

HRMS spectra of **4a**

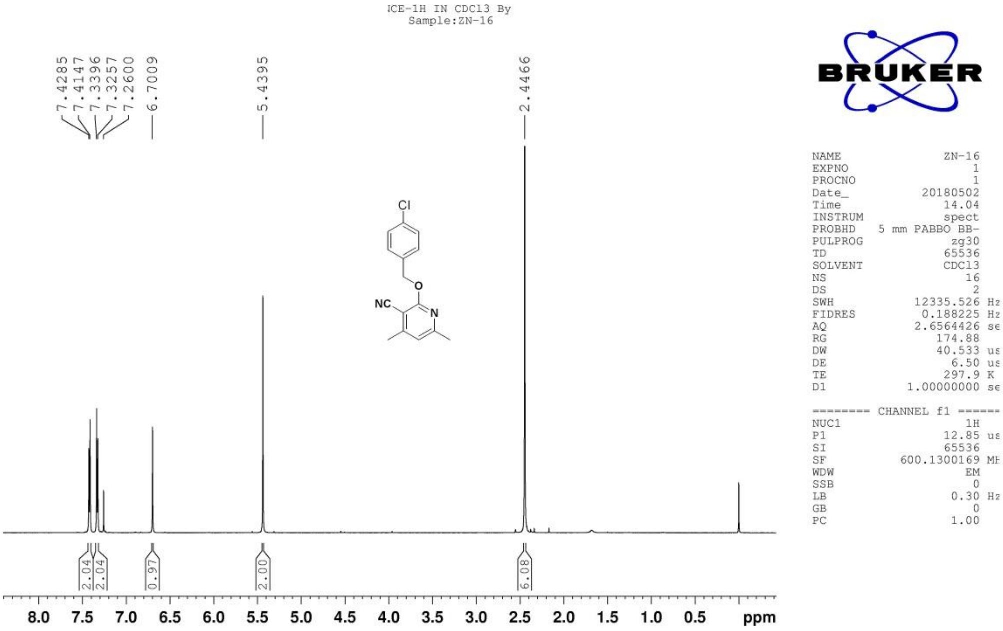

^{1}H NMR spectra of **4b**

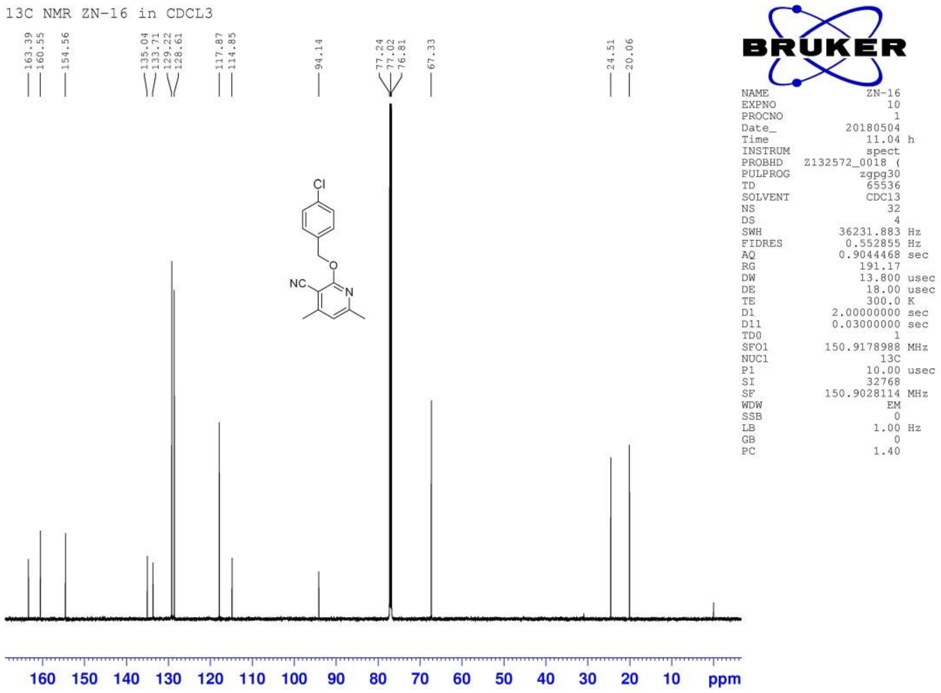

^{13}C NMR spectra of **4b**

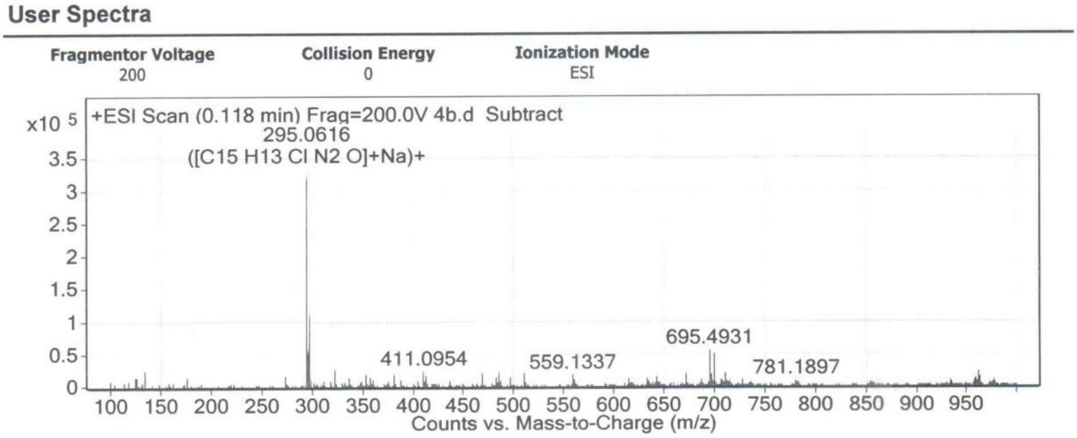

HRMS spectra of **4b**

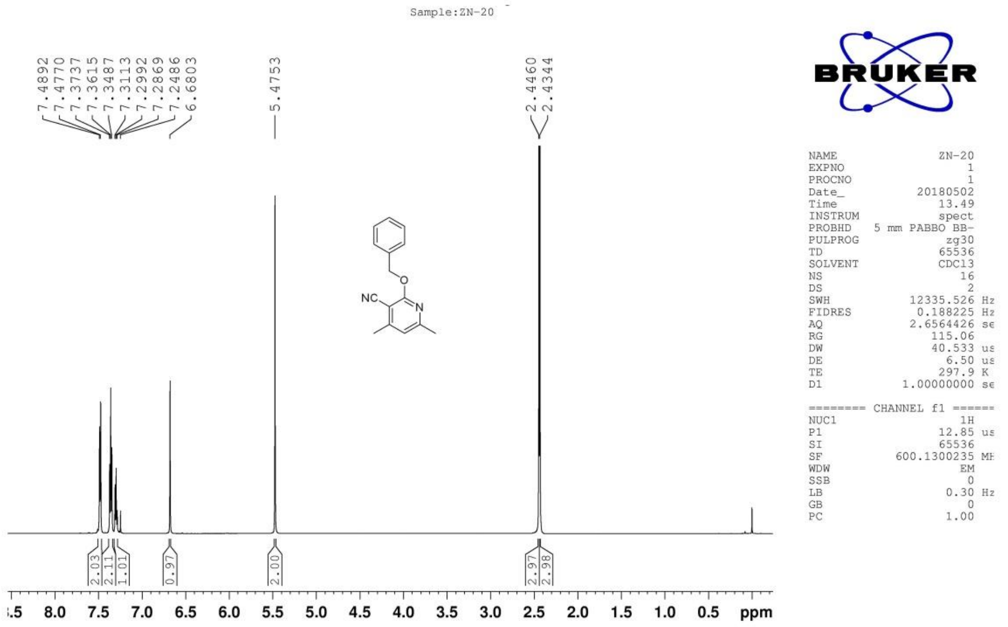

^{1}H NMR spectra of **4c**

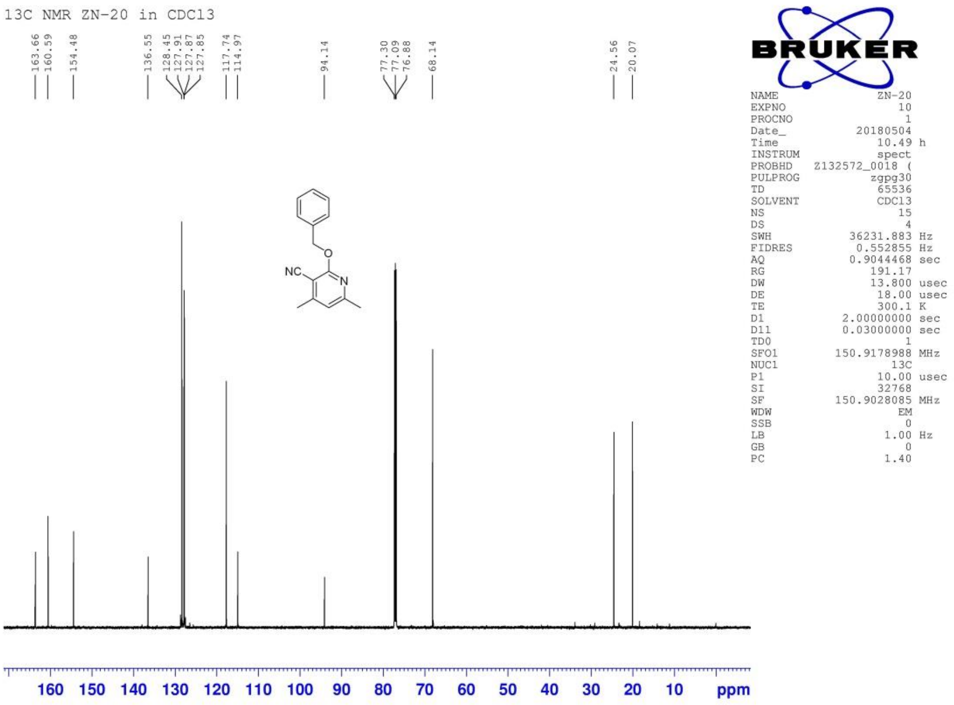

^{13}C NMR spectra of **4c**

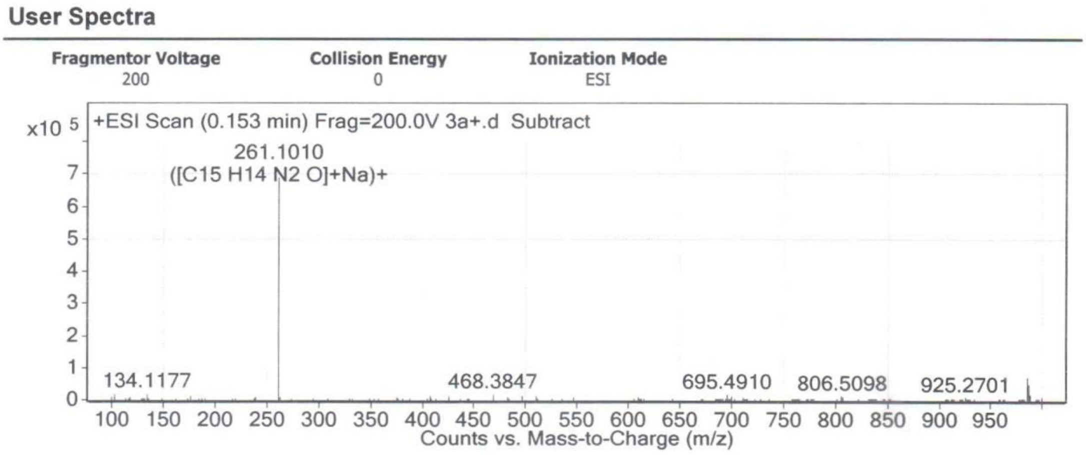

HRMS spectra of **4c**

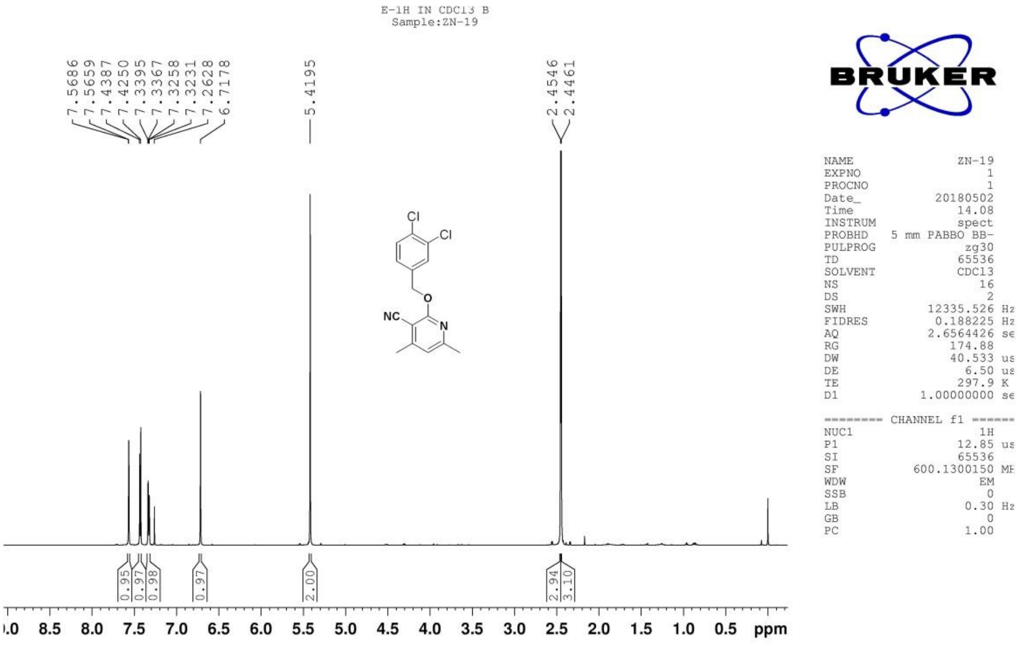

^{1}H NMR spectra of **4d**

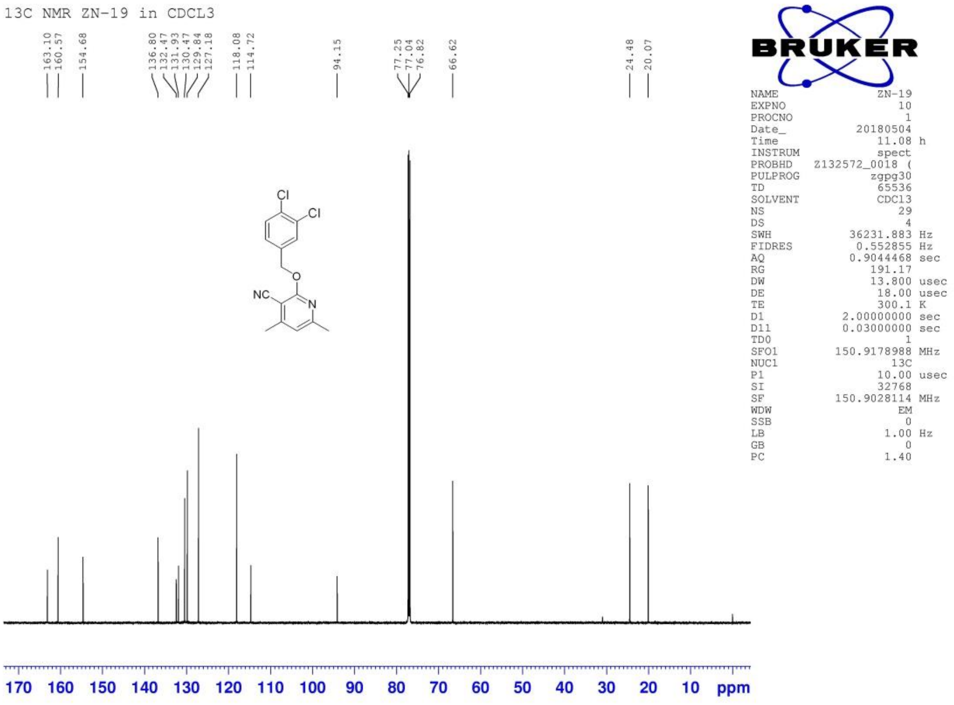

^{13}C NMR spectra of **4d**

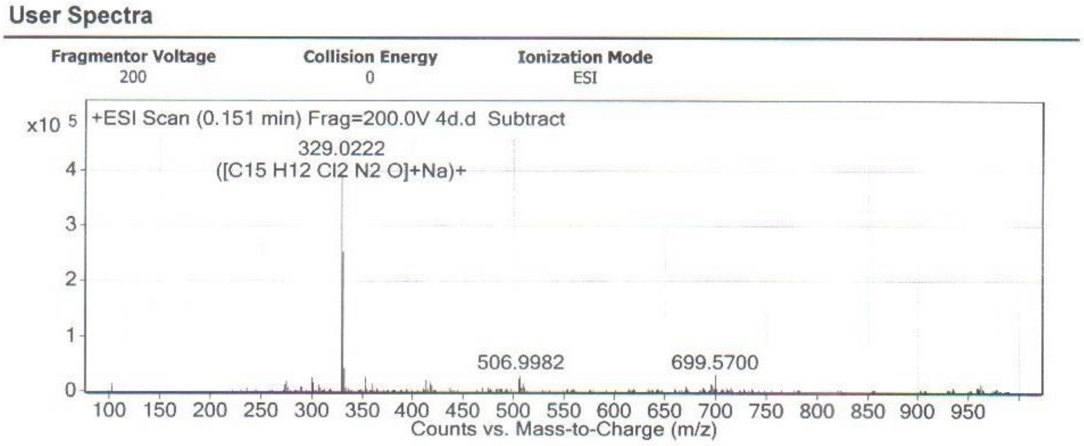

HRMS spectra of **4d**

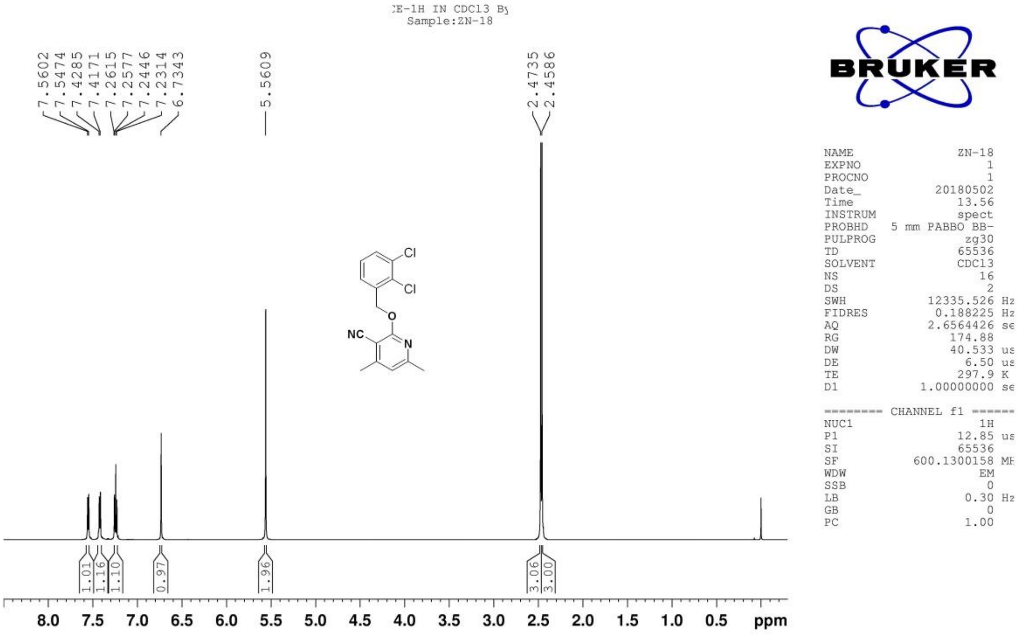

^{1}H NMR spectra of **4e**

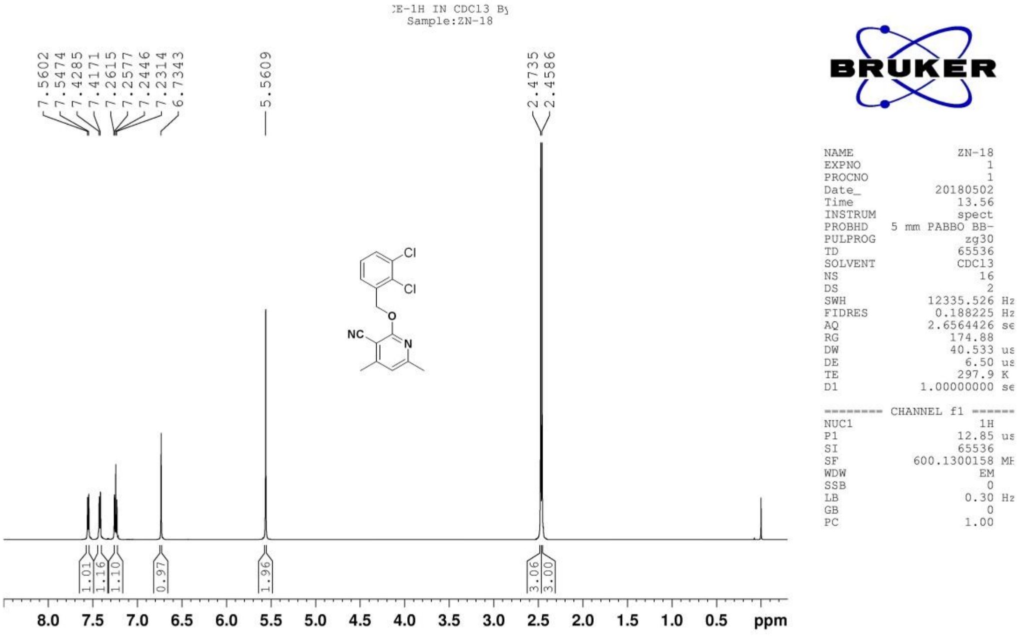

^{13}C NMR spectra of **4e**

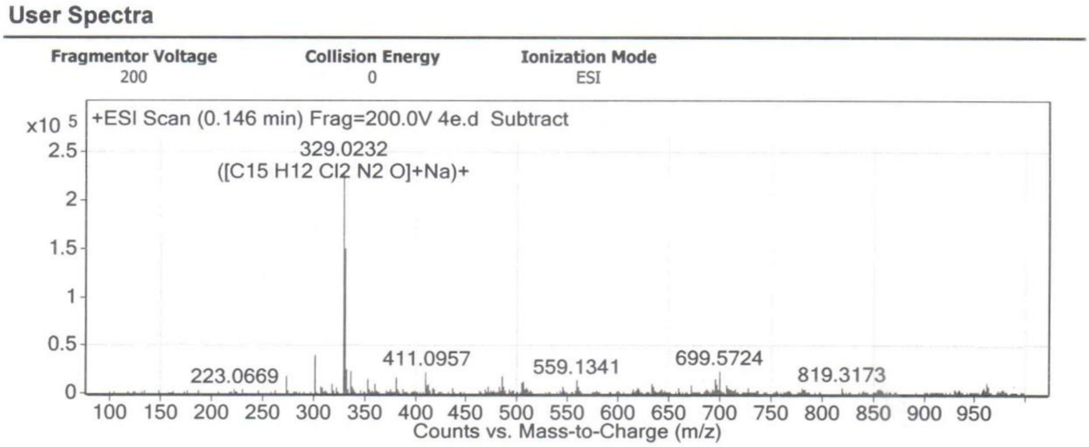

HRMS spectra of **4e**

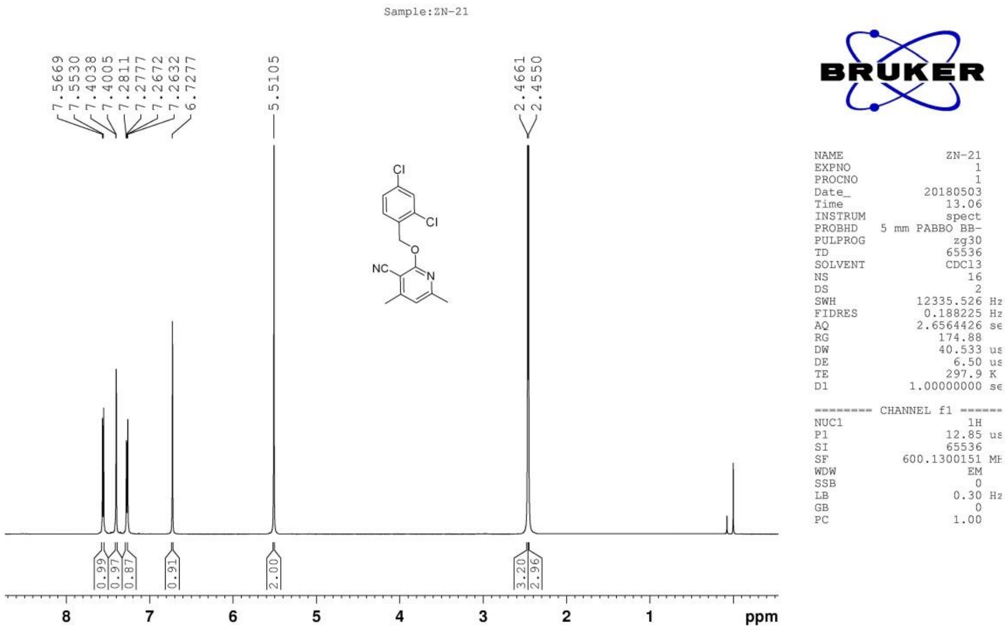

^{1}H NMR spectra of **4f**

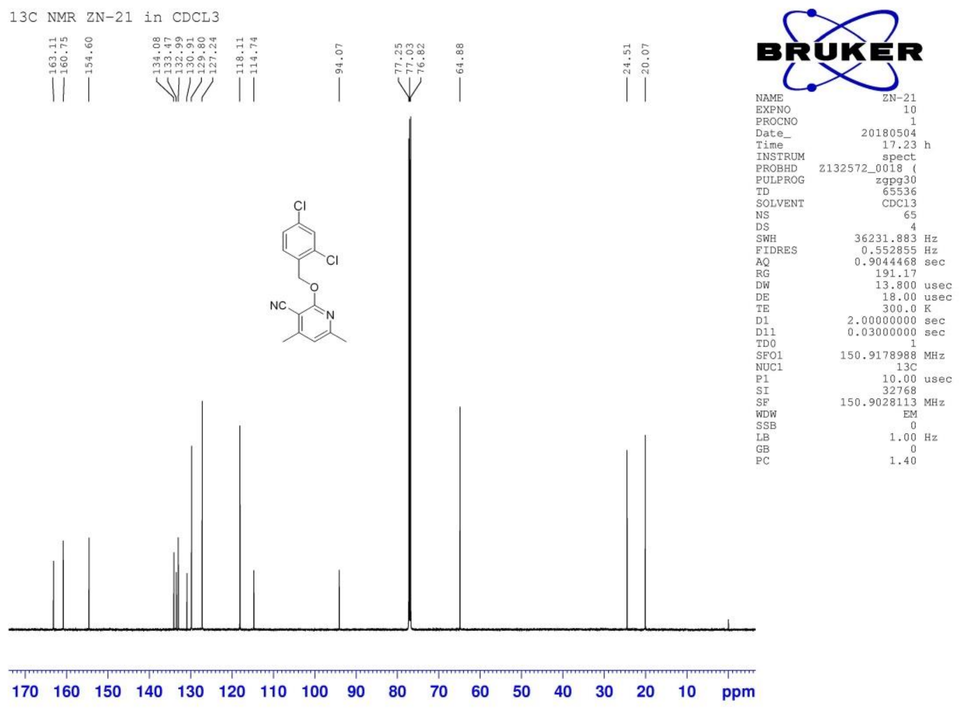

^{13}C NMR spectra of **4f**

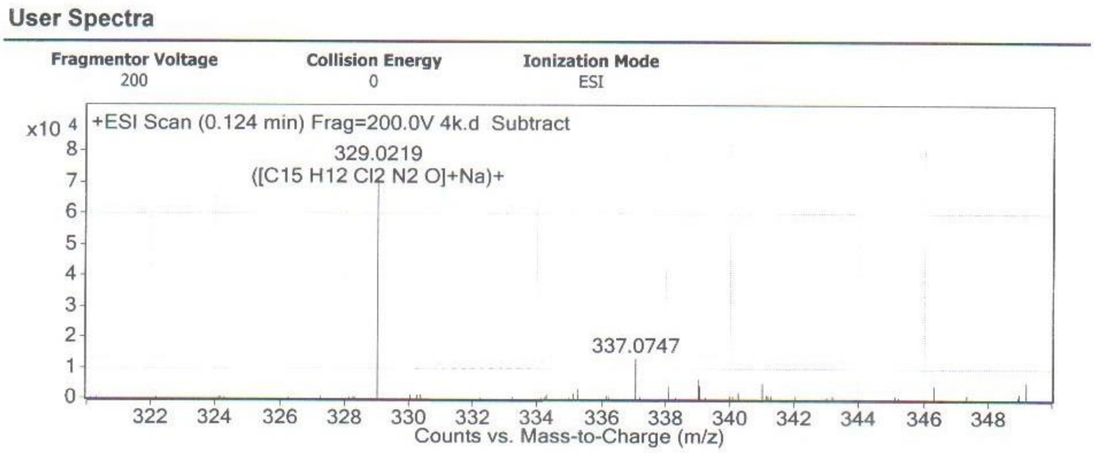

HRMS spectra of **4f**

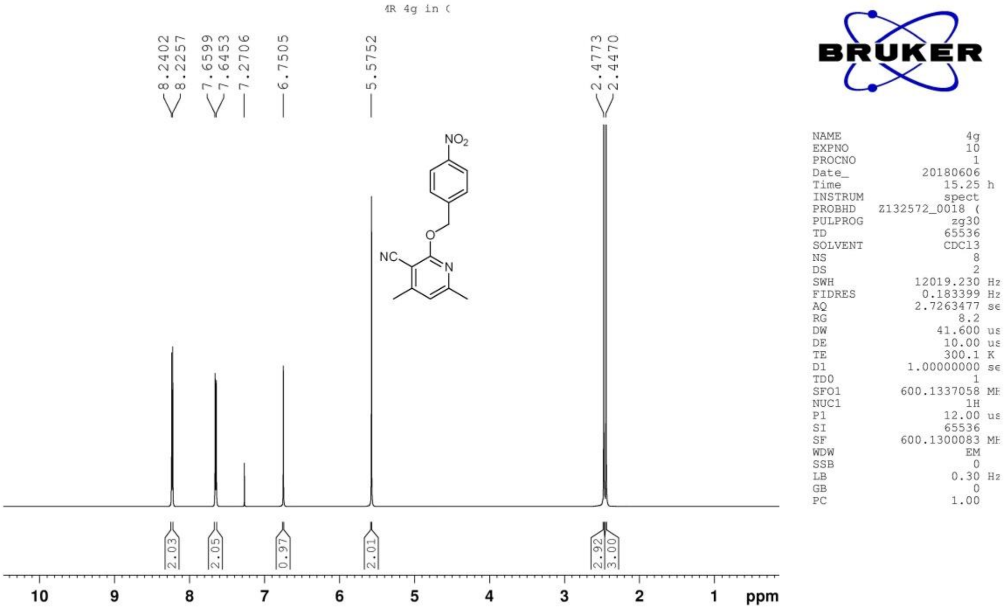

^{1}H NMR spectra of **4g**

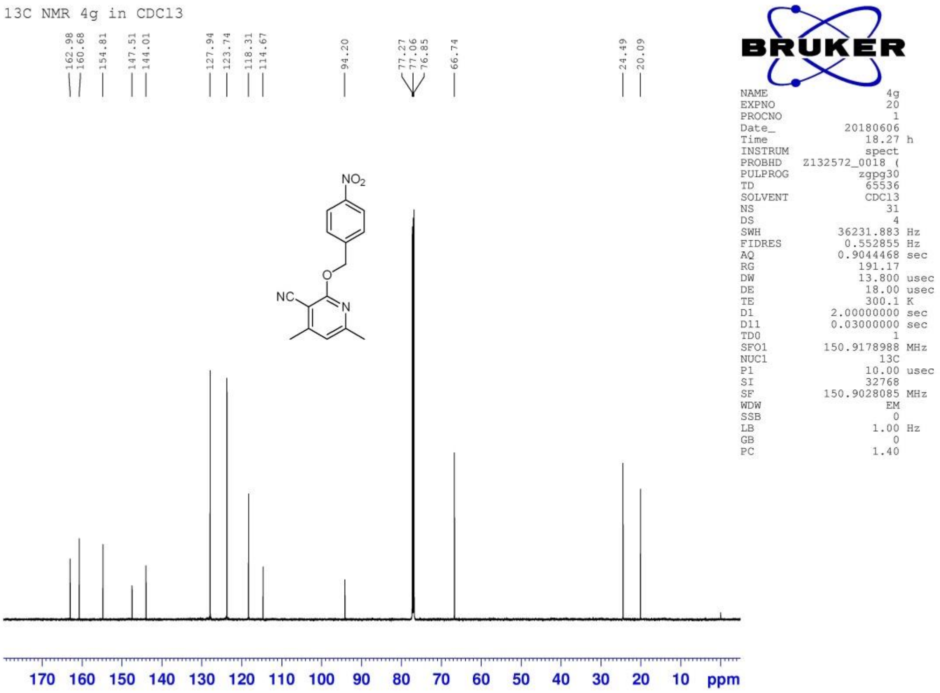

^{13}C NMR spectra of **4g**

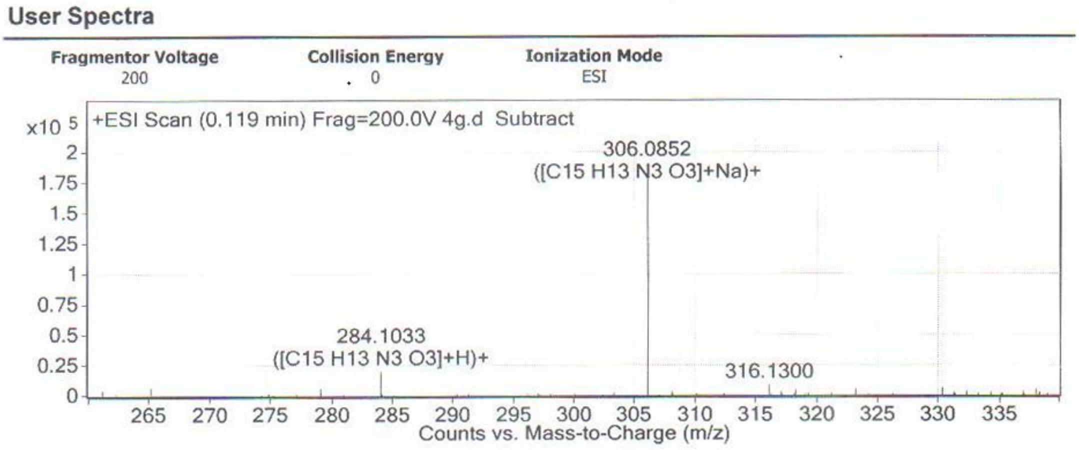

HRMS spectra of **4g**

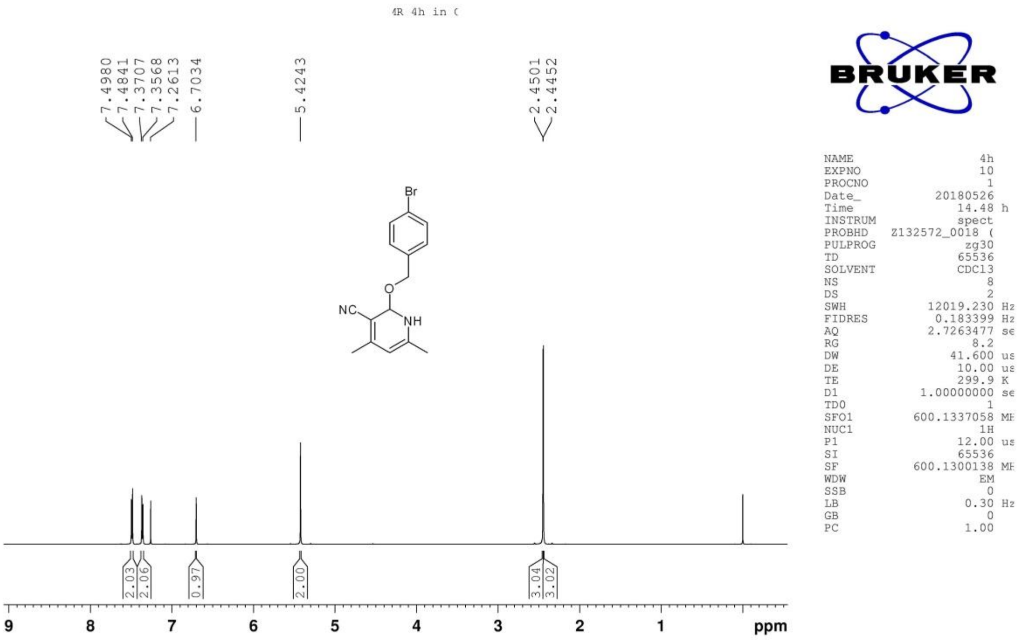

^{1}H NMR spectra of **4h**

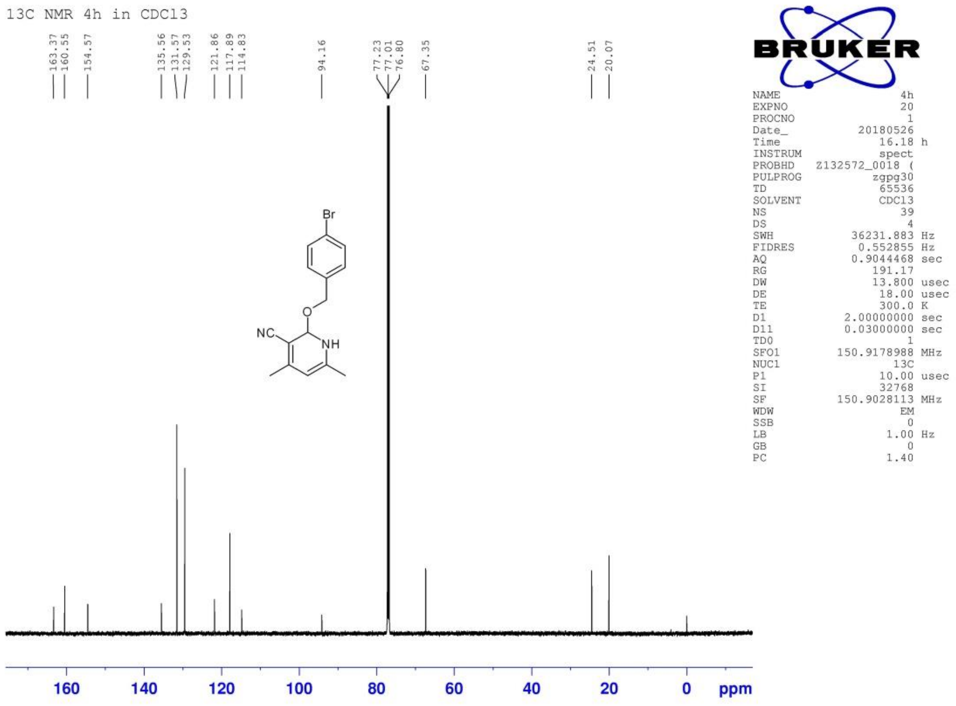

^{13}C NMR spectra of **4h**

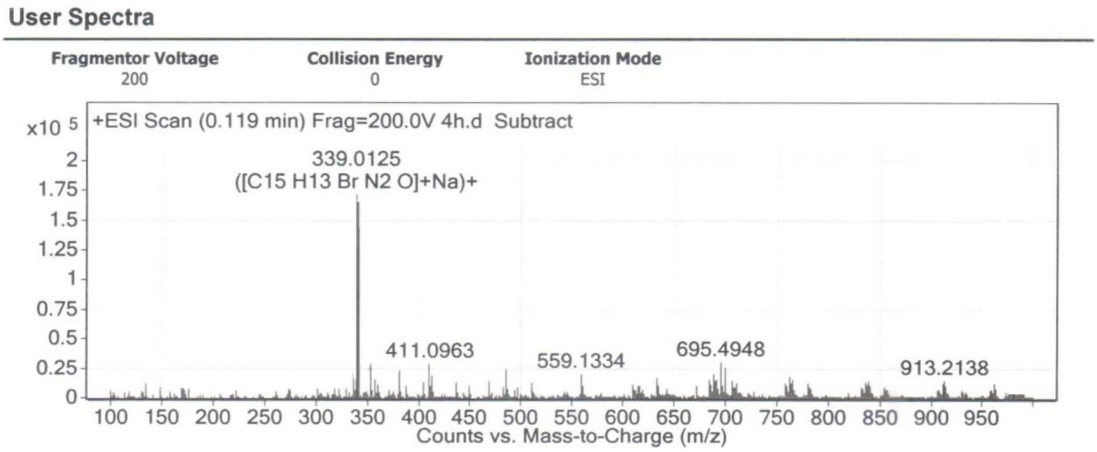

HRMS spectra of **4h**

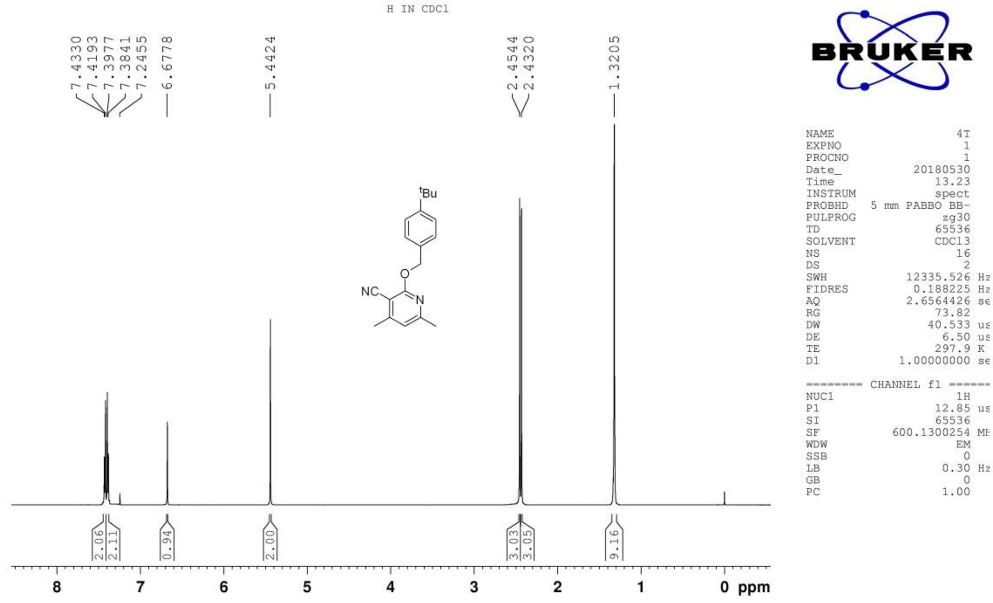

^{1}H NMR spectra of **4i**

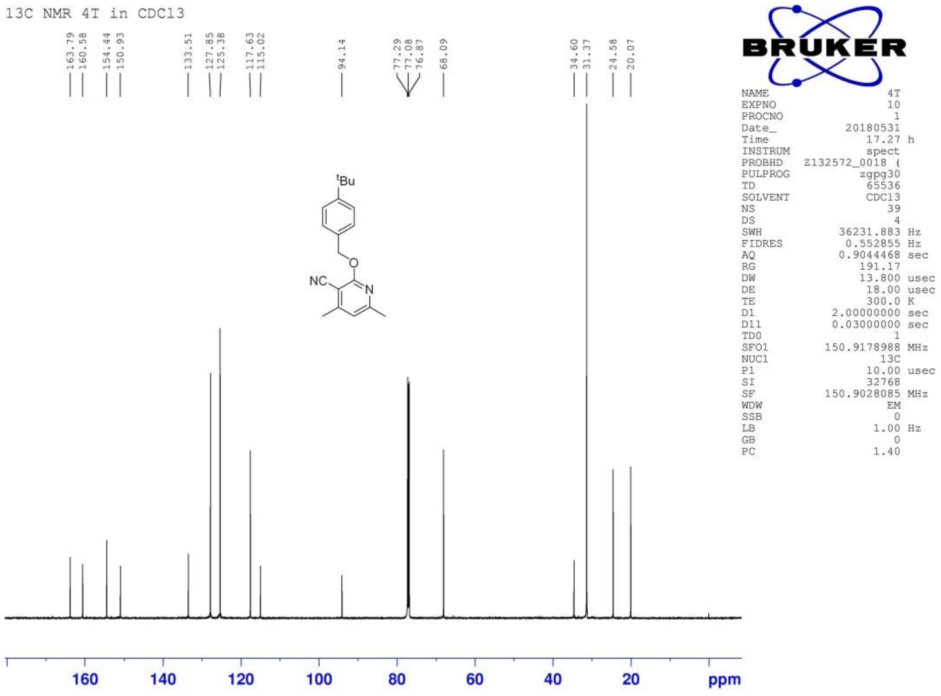

^{13}C NMR spectra of **4i**

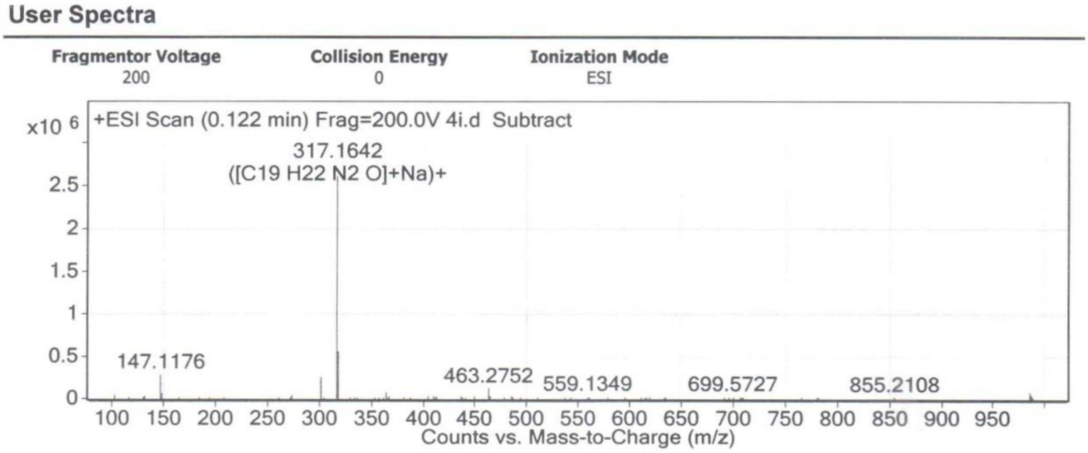

HRMS spectra of **4i**

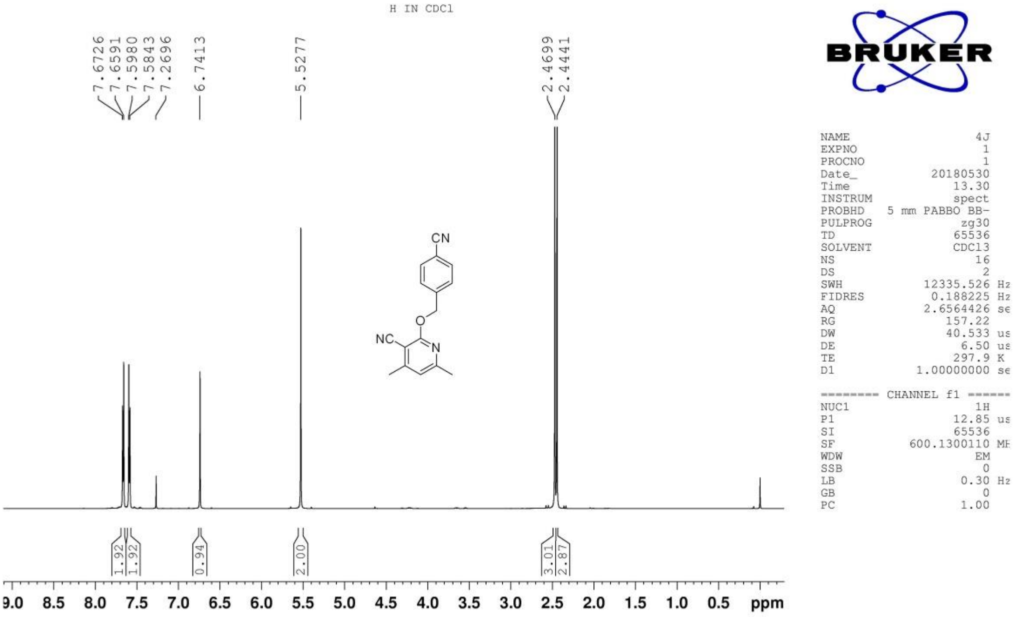

^{1}H NMR spectra of **4j**

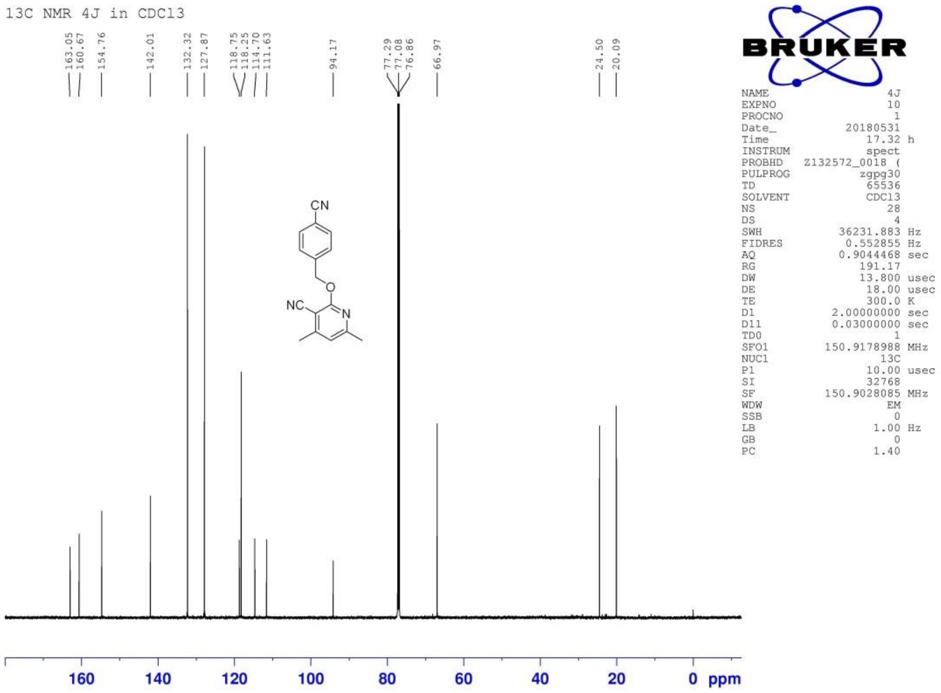

^{13}C NMR spectra of **4j**

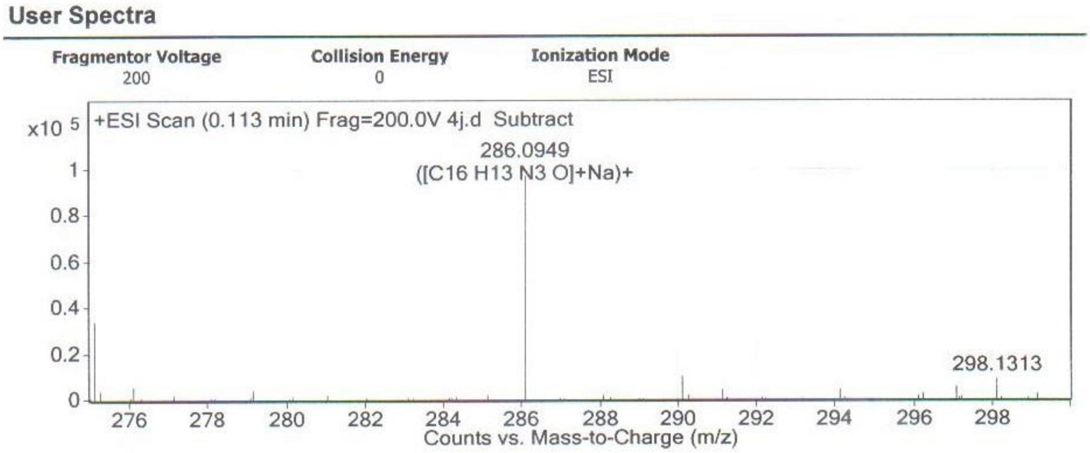

HRMS spectra of **4j**

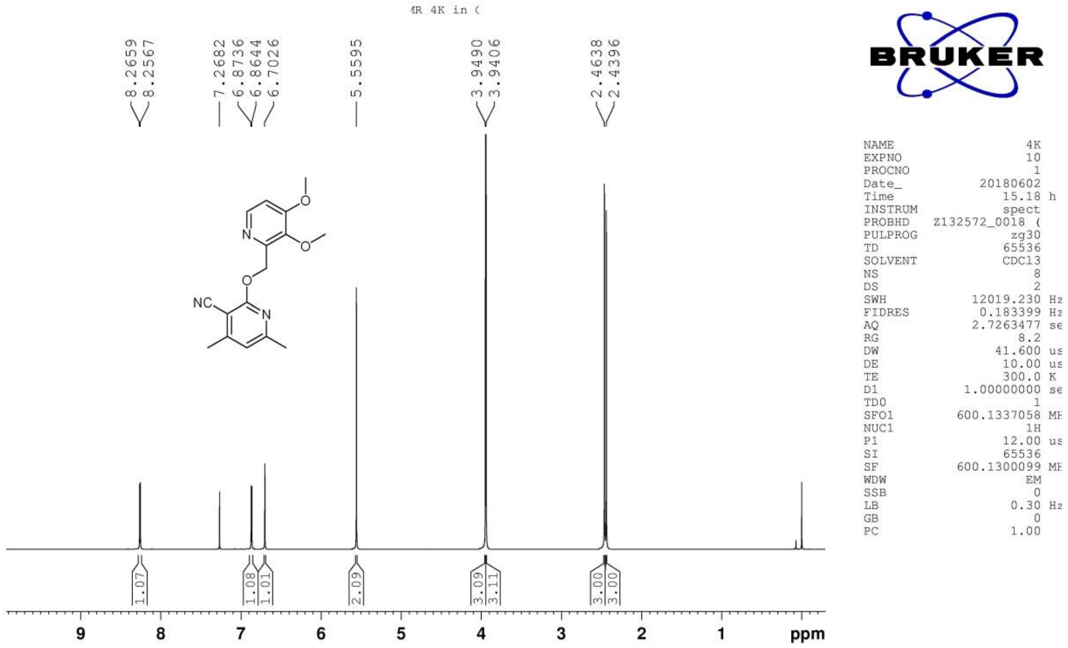

^{1}H NMR spectra of **4k**

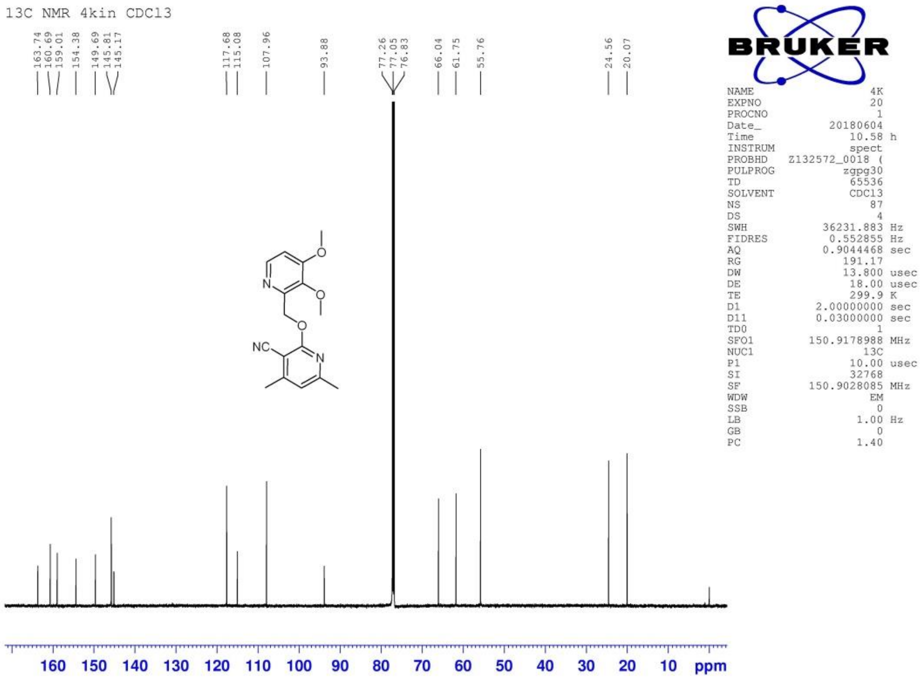

^{13}C NMR spectra of **4k**

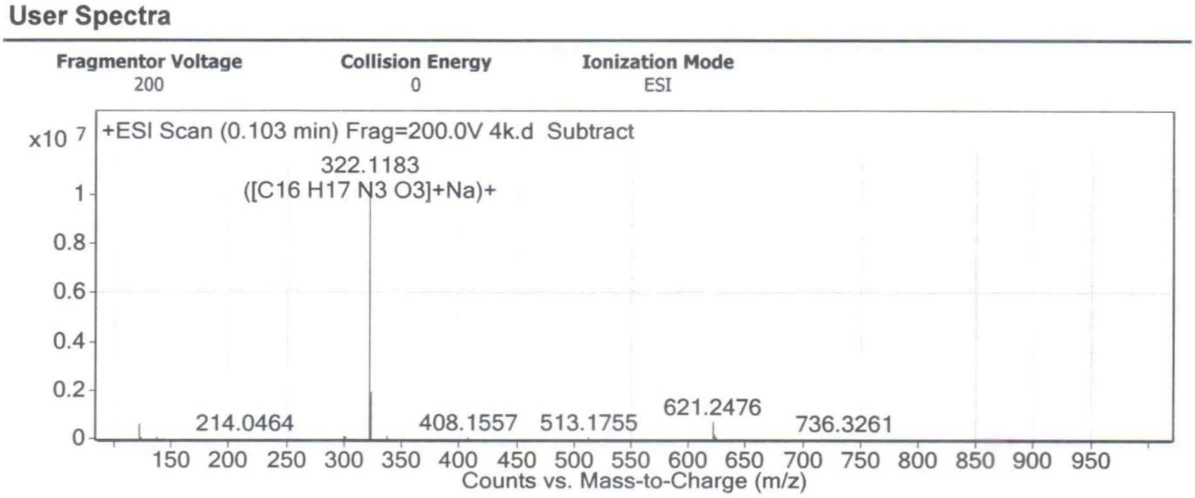

HRMS spectra of **4k**

9. ^{1}H NMR spectra of *N*-benzylation product 5

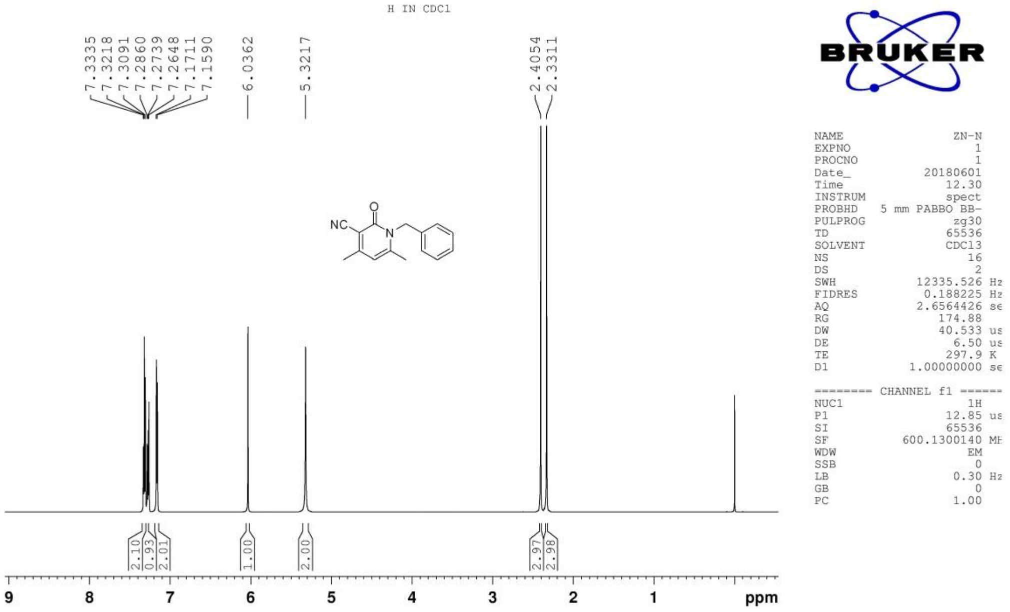

Supplement: Supplementary file 1 [file molecules-23-01784-s001.pdf]
